# Phase 2b study of evocalcet (KHK7580), a novel calcimimetic, in Japanese patients with secondary hyperparathyroidism undergoing hemodialysis: A randomized, double-blind, placebo-controlled, dose-finding study

**DOI:** 10.1371/journal.pone.0204896

**Published:** 2018-10-31

**Authors:** Tadao Akizawa, Ryutaro Shimazaki, Masafumi Fukagawa, Evocalcet Study Group

**Affiliations:** 1 Division of Nephrology, Department of Medicine, Showa University School of Medicine, Namics 301, Takanawa, Minato-ku, Tokyo, Japan; 2 R&D Division, Kyowa Hakko Kirin Co., Ltd., Tokyo, Japan; 3 Division of Nephrology, Endocrinology and Metabolism, Department of Internal Medicine, Tokai University School of Medicine, Kanagawa, Japan; University of Milan, ITALY

## Abstract

**Background:**

Evocalcet has been developed as a new calcimimetic agent for hemodialysis (HD) patients with secondary hyperparathyroidism (HDSHPT), eliciting fewer gastrointestinal symptoms and drug interactions. We evaluated the efficacy, safety, and optimal starting dose of evocalcet in HDSHPT.

**Methods:**

In this 3-week, Phase 2b, randomized, double-blind, placebo-controlled, multicenter, parallel-group, dose-finding study, Japanese HDSHPT with intact parathyroid hormone (iPTH) ≥240 pg/mL and serum calcium level corrected for albumin ≥8.4 mg/dL were randomized to evocalcet 0.5, 1, 2 mg/day administered orally or placebo under double-blind conditions, and cinacalcet 25 mg/day (open-label conditions).

**Results:**

In total, 152 HDSHPT were randomized. The mean ± standard deviation (median, interquartile range) of percent changes in iPTH from baseline to end of treatment were −8.40±25.43% (−12.16, 39.60), −10.56±22.86% (−14.24, 27.85), and −20.16±34.23% (−23.83, 39.05) in the evocalcet 0.5, 1, and 2 mg/day groups and 5.44±25.85% (3.52, 35.39) and −25.86±27.76% (−29.79, 34.15) in the placebo and cinacalcet groups, respectively. The dose-response profile for each evocalcet group vs placebo showed statistically significant differences for all contrast patterns. Whole PTH, corrected calcium, ionized calcium, phosphorus, and intact fibroblast growth factor 23 decreased after treatment initiation in the evocalcet and cinacalcet groups. Adverse events were observed in 30%–50% of patients (all groups). Incidence of adverse events was similar among all groups except for decreased calcium, which occurred more frequently in the evocalcet 2 mg and cinacalcet groups.

**Conclusions:**

The dose response and safety of all administered doses of evocalcet were confirmed, as well as the efficacy of evocalcet ≥1 mg in a strictly Japanese sample of HDSHPT. Therefore, evocalcet 1 mg was considered appropriate as an initial dose for HDSHPT.

## Introduction

Secondary hyperparathyroidism (SHPT) is a common complication of chronic kidney disease-mineral and bone disorder (CKD-MBD), often occurring in patients with end-stage renal disease [1]. Cinacalcet hydrochloride is an oral calcimimetic agent proven to be effective in SHPT, as it decreases serum intact parathyroid hormone (iPTH), calcium and phosphorus levels in hemodialysis (HD) patients with SHPT (HDSHPT) [2–6]. This effect may contribute to improved prognosis among patients with CKD-MBD. However, cinacalcet occasionally induces gastrointestinal (GI) adverse events, particularly nausea and vomiting. Additionally, cinacalcet has been associated with several drug–drug interactions, mainly attributed to its strong inhibitory effect on cytochrome P450 (CYP) 2D6 and its involvement in the CYP3A4-mediated metabolic pathway [7–10]. In particular, such GI adverse events may result in lower adherence and insufficient dosages [7], [10].

KHK7580 (hereafter, evocalcet) is expected to be a novel oral calcimimetic agent and an allosteric modulator that could show similar efficacy to cinacalcet for suppressing iPTH secretion with fewer GI adverse events [11]. Therefore, evocalcet may be a promising calcimimetic agent to effectively control HDSHPT with a wide safety margin, especially for patients who cannot be adequately treated with cinacalcet because of adverse drug reactions or drug–drug interactions. This Phase 2b clinical study aimed to compare the efficacy and safety of three different evocalcet doses (0.5, 1, and 2 mg/day), with placebo and cinacalcet 25 mg/day in Japanese HDSHPT. We also aimed to identify the optimal starting dose of evocalcet for the subsequent Phase 3 study on HDSHPT.

## Materials and methods

### Ethics

This study was performed in accordance with the principles of the Declaration of Helsinki, Pharmaceutical Affairs Law, Good Clinical Practice, and associated Japanese regulations. Each study center received institutional review board (IRB) approval; 12 institutions obtained approval from their own internal IRBs, and the remaining 28 institutions obtained approval from 11 externally contracted IRBs: Medical Corporation Bokoi Nikko Memorial Hospital Institutional Review Board (IRB), Koyasu Neurosurgical Clinic IRB, Review Board of Human Rights and Ethics for Clinical Studies, Koukeikai Sugiura Clinic IRB, Medical Corporation Showakai IRB, Kaikoukai Healthcare Corporation Nagoya Kyoritsu Hospital IRB, Fukui General Hospital IRB, Rakuwakai Otowa Hospital IRB, Medical Corporation Akane Tsuchiya General Hospital IRB, Medical Corporation Houmankai Umezu Clinic IRB, and Kouseikai Hospital IRB. Further details are listed in S1 Table. All patients provided written informed consent.

This study was registered in ClinicalTrials.gov under the identifier NCT02216656 and in the Japan Pharmaceutical Information Center, Clinical Trials Information (JapicCTI), under the identifier JapicCTI-142631.

### Patients

**Fig 1:**
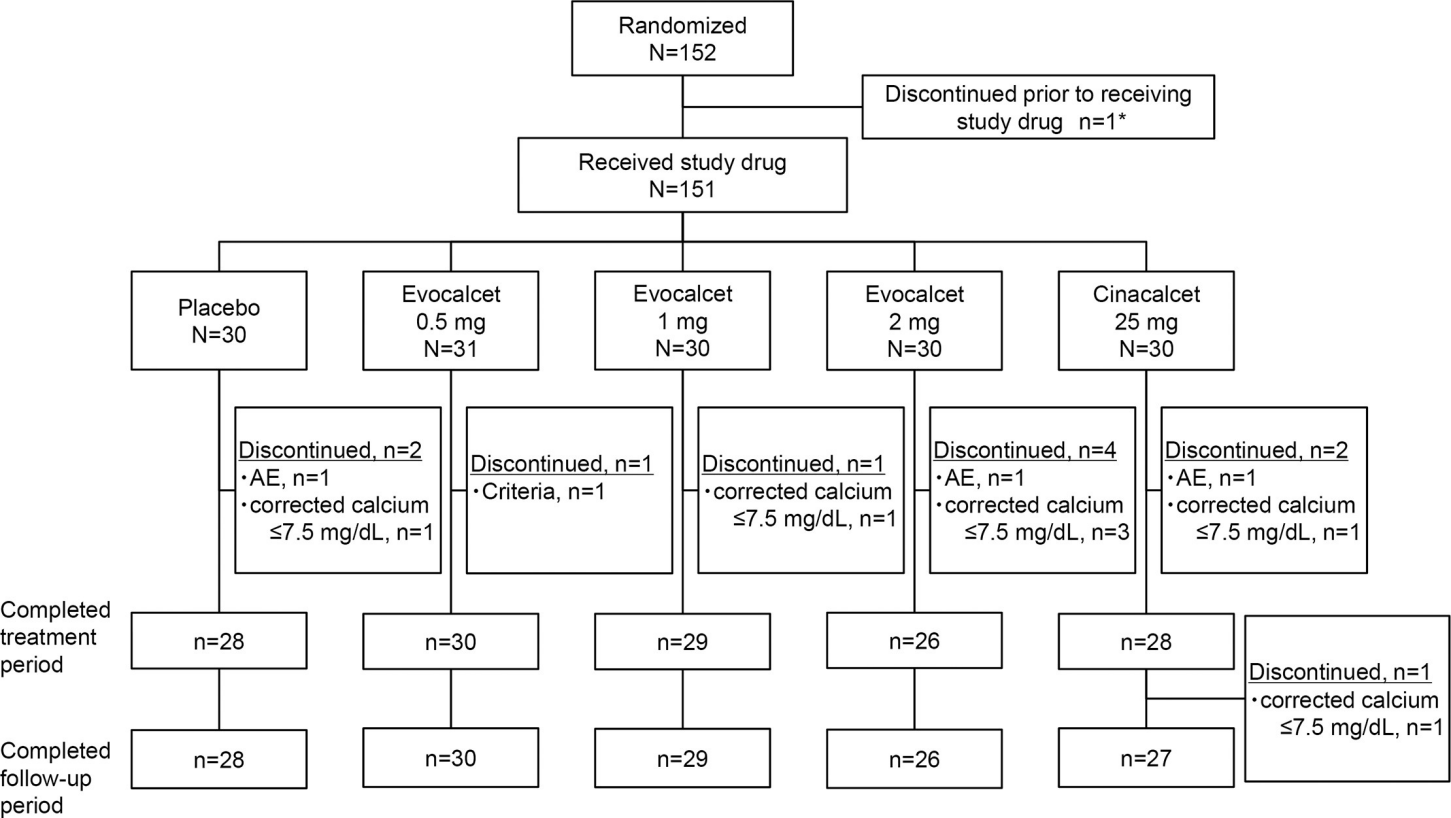
Patient disposition. *Tumor was identified by cervical ultrasonogram. AE, adverse event.

The inclusion criteria were 20–75 years of age at the time of consent, stable CKD treated with HD three times weekly for at least 12 weeks before screening, iPTH level ≥240 pg/mL and serum calcium level corrected for albumin ≥8.4 mg/dL at screening according to the guideline of the Japanese Society for Dialysis Therapy [12]. Detailed exclusion criteria are listed in the supporting information (S1 Text). A patient flow diagram is shown in Fig 1.

### Study design and treatments

**Fig 2:**
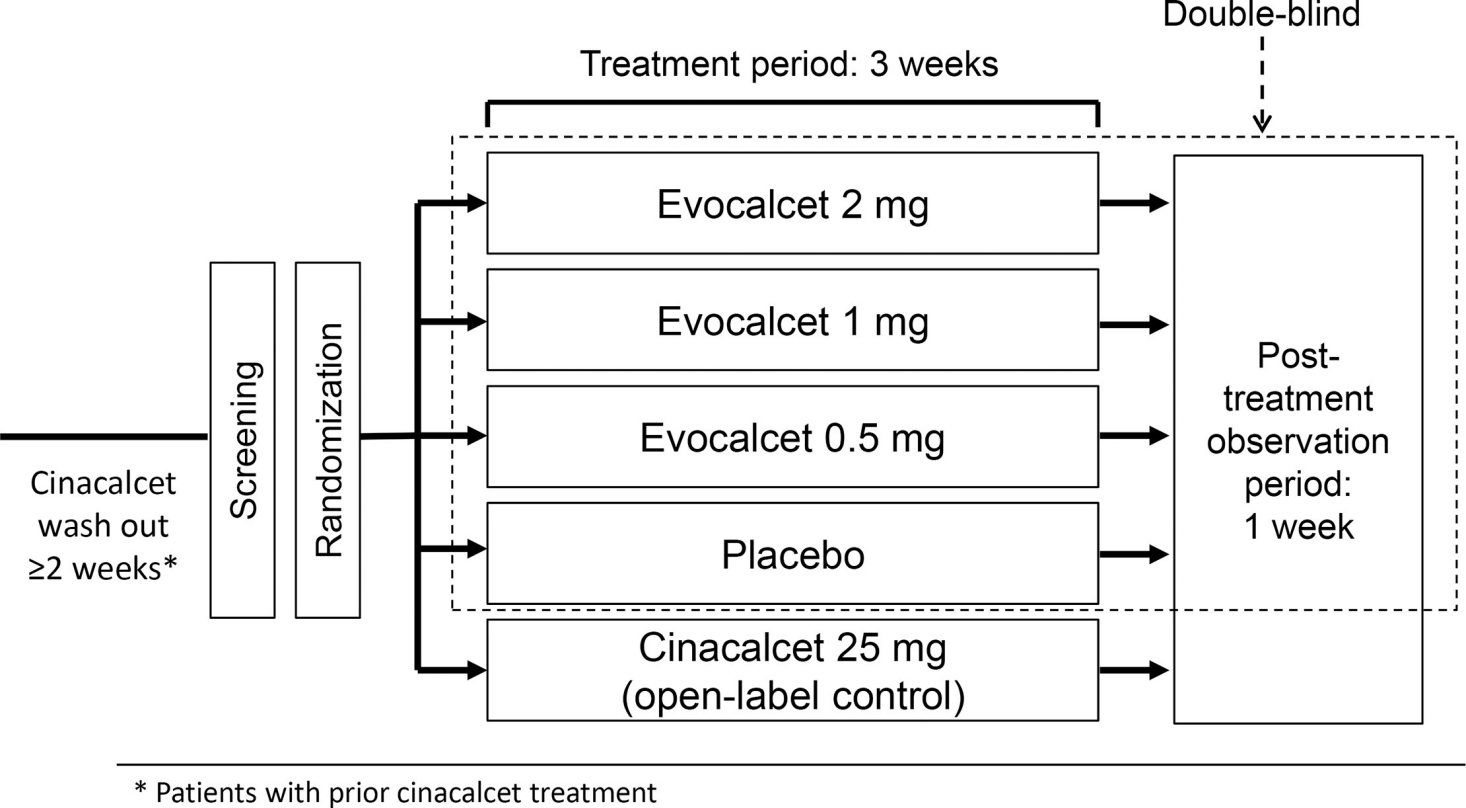
Study design. The area enclosed by the dotted line indicates the double-blind treatment phase. *Patients with prior cinacalcet treatment.

This was a Phase 2b, randomized, double-blind, placebo-controlled, multicenter, parallel-group, dose-finding, five-group study (including one open-label control group, Fig 2) with a 3-week treatment period and a 1-week post-treatment observation period. This study was conducted in 40 facilities in Japan (S1 Table). For an equal enrollment of patients at each facility, the number of contract cases for each facility was set to five cases.

Patients were randomized to the evocalcet 0.5, 1, or 2 mg; placebo; or cinacalcet group using a dynamic allocation procedure. Stratification factors were clinical trial site/institute, cinacalcet treatment history, corrected calcium at screening (<9.0 mg/dL or ≥9.0 mg/dL), and iPTH level at screening (<500 pg/mL or ≥500 pg/mL). Details of procedures applied to ensure blinding are summarized in the supporting information (S2 Text).

Blood samples were obtained and clinical laboratory tests were performed before the start of study treatment and starting dialysis. The study treatment began on Day 1, just before starting dialysis and after the maximum dialysis interval. Patients received the study drugs orally, once daily for 3 weeks. Patients who were assigned to the evocalcet 0.5, 1, and 2 mg and placebo groups received the study drug under double-blind conditions; those who were assigned to the cinacalcet group received the study drug under open-label conditions. End-of-treatment (EOT) assessments were performed on Day 22, followed by final assessments at the end of the follow-up period (Day 29). Cinacalcet was washed out from 2 weeks before the screening period. Other treatments, including active vitamin D, phosphate binders, calcium preparation and dialysis conditions, including dialysate calcium level, were not changed.

### Biochemical and other determinations

All biochemical analyses were conducted at a central clinical laboratory. iPTH level in serum was analyzed by modular analytics using the ECLIA method (reagent: ECLusys PTH; Roche Diagnostics K.K., Tokyo, Japan). Intact fibroblast growth factor (FGF) 23 level was measured using the FGF-23 ELISA Kit (Kainos Laboratories Inc., Tokyo, Japan), a microplate reader (Tecan Infinite M200, Tecan Japan Co., Ltd., Tokyo, Japan), and software (i-control, Tecan Japan Co., Ltd., Tokyo Japan). To detect any significant electrocardiographic findings, subjects underwent a resting 12-lead electrocardiography.

### Endpoints

The primary efficacy endpoint was the percent change in iPTH level at EOT. The secondary efficacy endpoints were the number and percentage (%) of patients who achieved an iPTH level of ≤240 pg/mL at EOT and the number (%) of patients who achieved a decrease in iPTH level of ≥30% from baseline at EOT. Other endpoints included the time-course change of iPTH, whole PTH, corrected calcium, ionized calcium, phosphorus, intact FGF23, and serum corrected calcium-phosphorus product at each time point and at EOT. Treatment safety was based on the reporting of adverse events (AEs). All AEs that occurred after the initiation of study treatment were tabulated by type using System Organ Class and categorized according to Preferred Term of MedDRA/J version 17.1.

### Statistical analyses

For the primary efficacy endpoint (percent change in the iPTH level from Day 1 to EOT) a dose-response profile was examined using seven types of contrast patterns involving the respective mean values in the evocalcet 0.5, 1, and 2 mg and placebo groups. The level of significance was set at 5%, and all tests were two-sided. All statistical analyses were performed using SAS version 9.2 (SAS Institute, Inc., Cary, NC, USA).

For the primary and secondary endpoints, missing data at EOT were imputed until Day 22 using the last observation carried forward method (LOCF). Categorical data were summarized as frequency and percentage, and continuous data were summarized using descriptive statistics, including number of patients (%), mean (standard deviation [SD]), and minimum, median, and maximum. The mean for each group and the corresponding 95% confidence interval (CI) were calculated by analysis of variance. The mean differences from placebo and the 95% CI for the mean were also calculated.

The target sample size per group was set at 30 patients (150 patients in total). The target number of patients in evocalcet and placebo groups (28 patients for each group) provided 90% power to detect at least one of the contrast patterns under multiplicity adjustment using a permutation method, assuming that the mean percent change in iPTH level at EOT of evocalcet 0.5, 1, and 2 mg and placebo groups were −6.71%, −13.42%, −19.80%, and 0%, respectively, and that the SD of all groups was 22.47%, with a two-sided significance level of 5% [13]. In the cinacalcet group, the target number of patients (28 patients) was calculated considering a previous study [7]. Given that some patients could drop out or be excluded from the per protocol set, the target number of patients receiving study treatment was set at 30 per group. The data analysis sets are described in detail in the supporting information (S3 Text).

## Results

### Patient disposition and baseline demographic characteristics

In total, 152 patients were recruited and were distributed evenly across the 40 facilities. All 152 patients were randomized, and one patient discontinued the study before initiating treatment. Thus, 151 patients received treatment (Fig 1). Each of the five groups included 30 patients, except for the evocalcet 0.5 mg group, which included 31 patients. In total, 11 patients (1, 1, 4, 2, and 3 in the evocalcet 0.5, 1, and 2 mg, placebo, and cinacalcet groups, respectively) discontinued the study during the treatment period. More than 90% of the patients (n = 140) completed the treatment period and follow-up. A total of 144 patients (30, 30, 28, 28, and 28, respectively) were included in the per protocol set, and 151 patients (31, 30, 30, 30, and 30, respectively) were included in the safety analysis set.

The background characteristics of the patients in each group (safety analysis set) are shown in Table 1. At least half of the patients in each group had been previously treated with cinacalcet. Specifically, the number of patients previously treated with cinacalcet at baseline in each group was 16 (51.6%) in the evocalcet 0.5 mg group, 16 (53.3%) in the evocalcet 1 mg group, 15 (50.0%) in the evocalcet 2 mg group, 17 (56.7%) in the placebo group, and 16 (53.3%) in the cinacalcet 25 mg group. The mean±SD iPTH levels at baseline were 405.0±159.2, 360.0±134.1, 400.3±159.4, 409.3±146.0, and 444.5±161.3 pg/mL in the per protocol set of each group, respectively.

**Table 1:** Baseline demographic and clinical characteristics of patients in the five groups (Safety analysis set).

	Placebo	Evocalcet 0.5 mg	Evocalcet 1 mg	Evocalcet 2 mg	Cinacalcet 25 mg
	N = 30	N = 31	N = 30	N = 30	N = 30
Sex, male	25 (83.3)	19 (61.3)	21 (70.0)	18 (60.0)	20 (66.7)
Age, years (mean±SD)	58.1±10.2	58.4±9.5	59.8±10.7	56.6±10.2	58.2±10.4
≥65	8 (26.7)	9 (29.0)	15 (50.0)	5 (16.7)	10 (33.3)
Dry weight, kg (mean±SD)	65.31±13.81	61.29±15.83	59.87±11.55	58.16±12.45	64.61±12.88
Body mass index, kg/m^2^ (mean±SD)	24.78±4.46	24.17±4.41	23.70±3.08	23.22±3.73	25.50±3.85
Duration of dialysis, months (mean±SD)	136.9±114.5	123.7±98.2	112.0±78.3	160.9±110.7	122.7±104.7
Previous use of cinacalcet	17 (56.7)	16 (51.6)	16 (53.3)	15 (50.0)	16 (53.3)
Primary disease					
Diabetic nephropathy	8 (26.7)	8 (25.8)	10 (33.3)	7 (23.3)	6 (20.0)
Chronic glomerulonephritis	10 (33.3)	10 (32.3)	11 (36.7)	15 (50.0)	13 (43.3)
Nephrosclerosis	4 (13.3)	4 (12.9)	2 (6.7)	1 (3.3)	4 (13.3)
Polycystic kidney disease	2 (6.7)	1 (3.2)	1 (3.3)	1 (3.3)	2 (6.7)
Chronic pyelonephritis	0	0	0	1 (3.3)	0
Other	6 (20.0)	8 (25.8)	6 (20.0%)	5 (16.7)	5 (16.7)
Complications					
Diabetes	10 (33.3)	11 (35.5)	10 (33.3)	7 (23.3)	9 (30.0)
Congestive heart failure	1 (3.3)	0	1 (3.3)	0	1 (3.3)
Long QT syndrome	0	0	0	0	1 (3.3)
Type of dialysis					
Hemodialysis	20 (66.7)	26 (83.9)	24 (80.0)	22 (73.3)	20 (66.7)
Hemodiafiltration	9 (30.0)	5 (16.1)	6 (20.0)	7 (23.3)	7 (23.3)
Other	1 (3.3)	0	0	1 (3.3)	3 (10.0)
Vitamin D					
Intravenous	19 (63.3)	15 (48.4)	16 (53.3)	20 (66.7)	15 (50.0)
Oral	9 (30.0)	7 (22.6)	5 (16.7)	3 (10.0)	7 (23.3)
Phosphate binder					
Calcium-based	18 (60.0)	18 (58.1)	17 (56.7)	15 (50.0)	19 (63.3)
Non-calcium-based	21 (70.0)	23 (74.2)	22 (73.3)	24 (80.0)	23 (76.7)
iPTH, pg/mL (mean±SD)	411.2±143.7	404.4±156.6	360.0±134.1	397.0±157.5	441.3±156.3
Corrected calcium, mg/dL (mean±SD)	9.45±0.70	9.41±0.74	9.68±0.74	9.46±0.87	9.37±0.57
Phosphorus, mg/dL (mean±SD)	5.49±1.36	6.06±1.53	5.33±1.25	5.81±1.35	5.43±1.26

Data in the table are presented as number of patients (%), unless otherwise indicated. iPTH, intact parathyroid hormone; SD, standard deviation

### Efficacy endpoints

#### Primary endpoint

**Fig 3:**
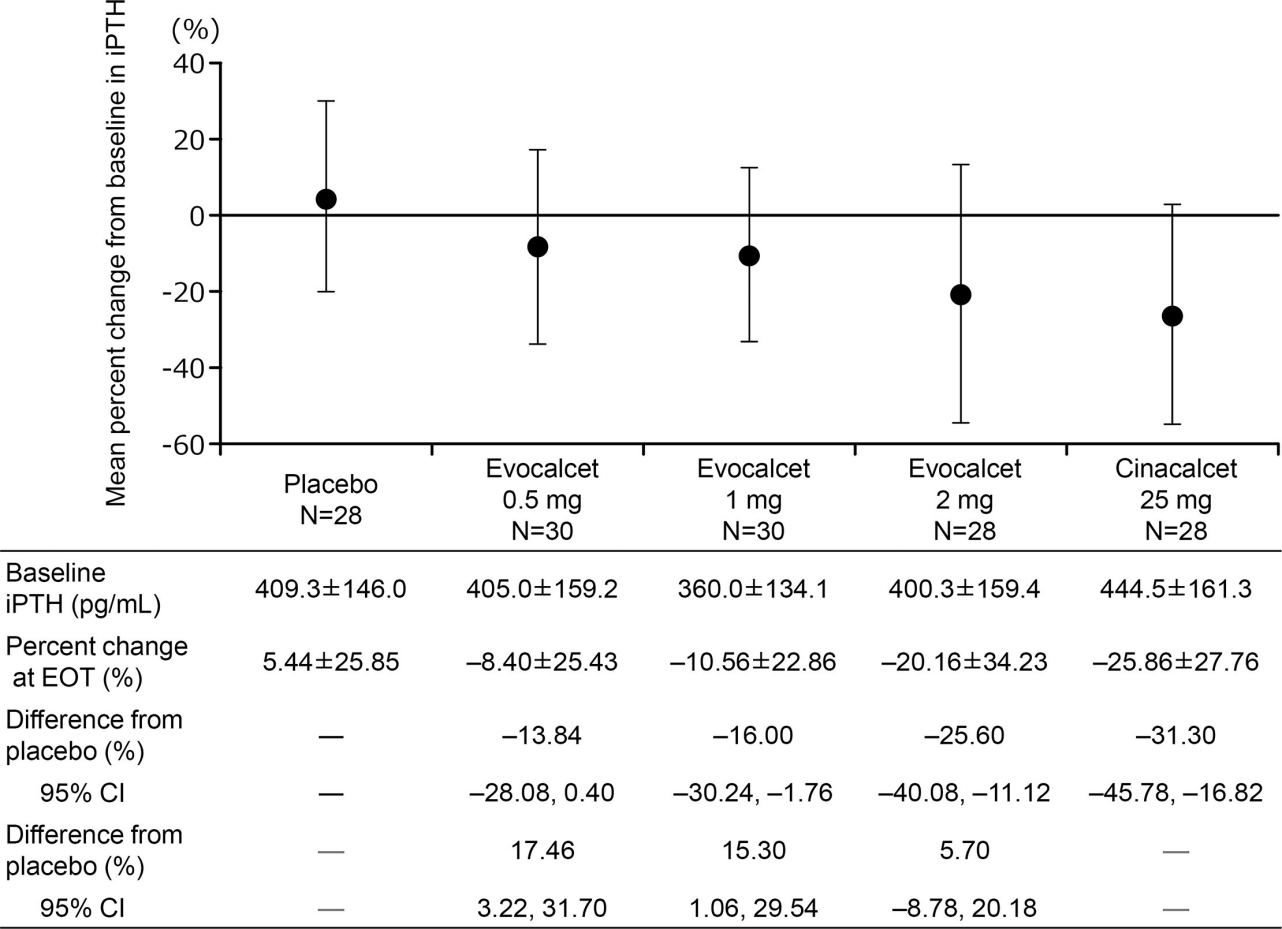
Point estimate of percent changes in iPTH at EOT. The error bars indicate standard deviation. iPTH, intact parathyroid hormone; EOT, end of treatment; CI, confidence interval.

**Fig 4:**
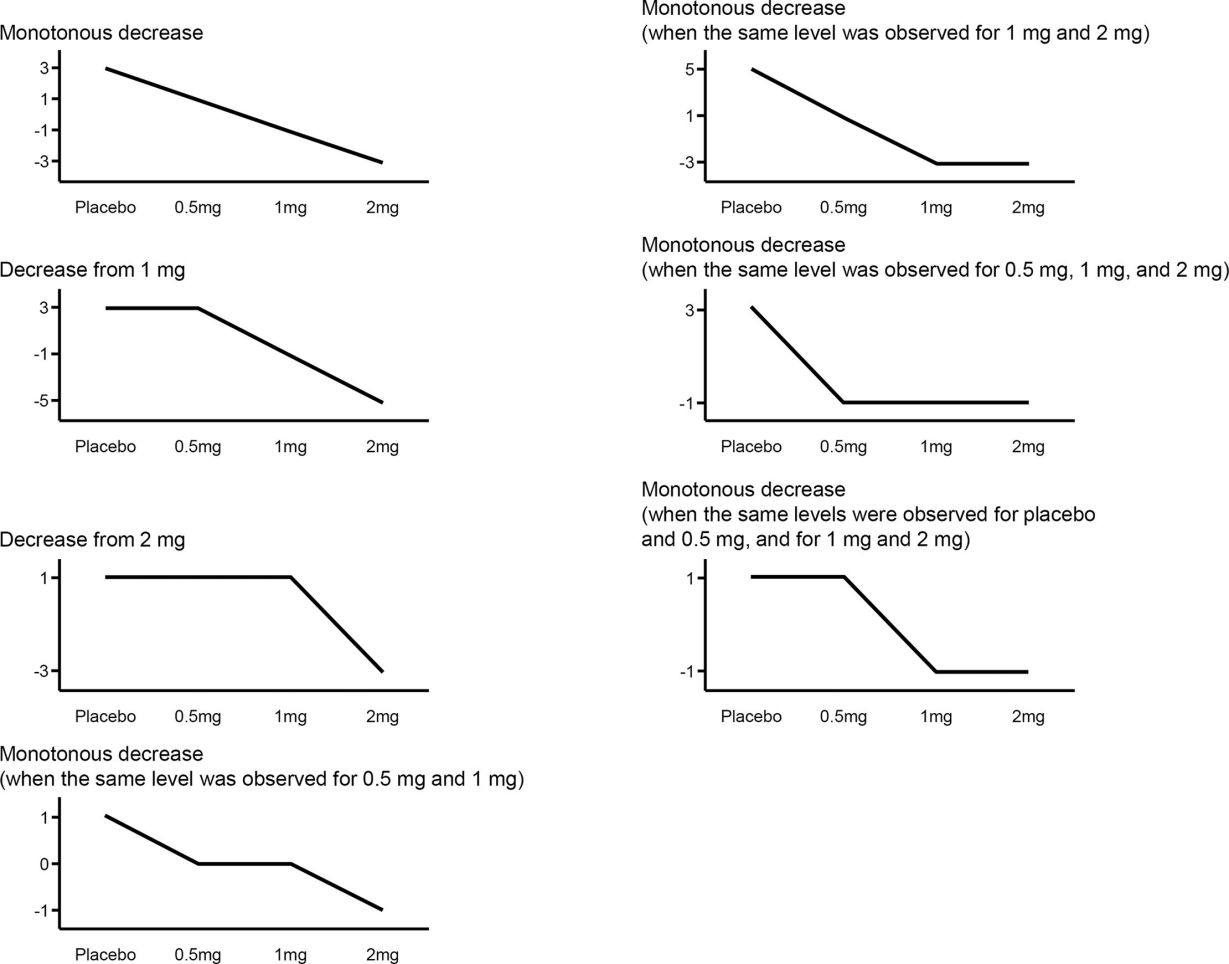
Contrast patterns used for the dose-response profile of evocalcet. The dose-response profile was examined using seven types of contrast patterns involving the respective mean values in the evocalcet 0.5, 1, and 2 mg and placebo groups. The contrast coefficients are displayed on the y-axis of each graph.

Fig 3 shows the point estimate of the percent change in iPTH at EOT. The mean±SD (median, interquartile range) percent changes in iPTH level from baseline to EOT were −8.40±25.43% (−12.16, 39.60), −10.56±22.86% (−14.24, 27.85), −20.16±34.23% (−23.83, 39.05), 5.44±25.85% (3.52, 35.39) and −25.86±27.76% (−29.79, 34.15) in the evocalcet 0.5, 1, and 2 mg, placebo, and cinacalcet groups, respectively. The missing data at EOT of four patients, one in the evocalcet 1 mg group and three in the evocalcet 2 mg group, were imputed by LOCF. The dose-response profile was examined using seven types of contrast patterns involving the respective mean percent changes in iPTH level from baseline to EOT in each group (Table 2 and Fig 4). Statistically significant differences were observed between placebo and each evocalcet groups for all contrast patterns. As the 95% CIs for the difference of the percent change in iPTH level in the evocalcet 1 and 2 mg groups from the placebo did not cross 0, the efficacy of evocalcet was verified at 1 mg or more.

**Table 2:** Dose-response profile (contrast patterns).

Contrast pattern	*P*-value
Monotonous decrease	0.002
Decrease from 1 mg	0.009
Decrease from 2 mg	0.029
Monotonous decrease (when the same level was observed for 0.5 mg and 1 mg)	0.002
Monotonous decrease (when the same level was observed for 1 mg and 2 mg)	0.004
Monotonous decrease (when the same level was observed for 0.5 mg, 1 mg, and 2 mg)	0.007
Monotonous decrease (when the same levels were observed for placebo and 0.5 mg, and for 1 mg and 2 mg)	0.022

The end of treatment visit occurred on Day 22 or at the early discontinuation visit. The *P*-value is adjusted for the multiplicity of tests by the permutation method.

#### Secondary endpoints

**Fig 5:**
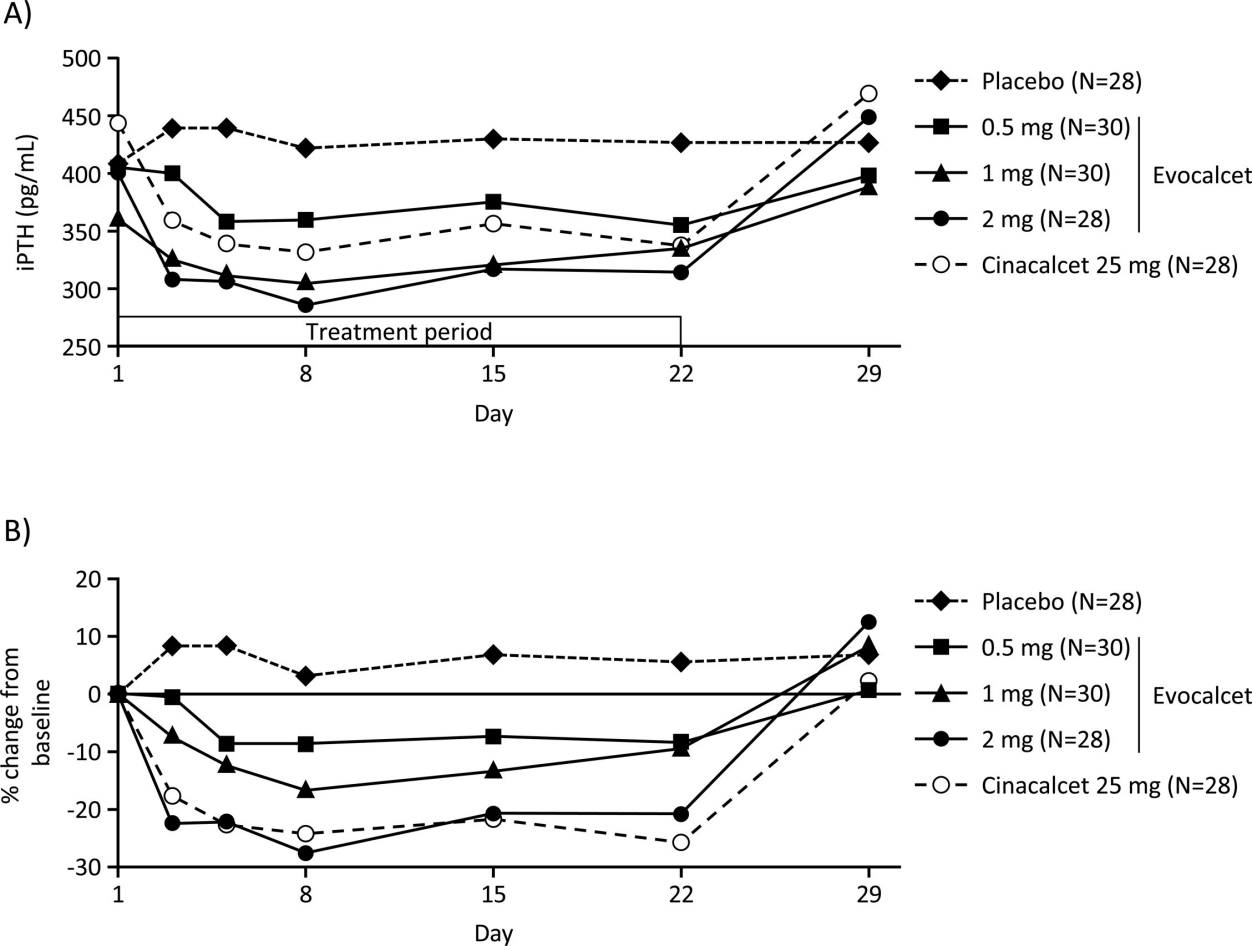
**Time-course profile of iPTH (A) and percent change from baseline (B).** iPTH, intact parathyroid hormone.

**Fig 6:**
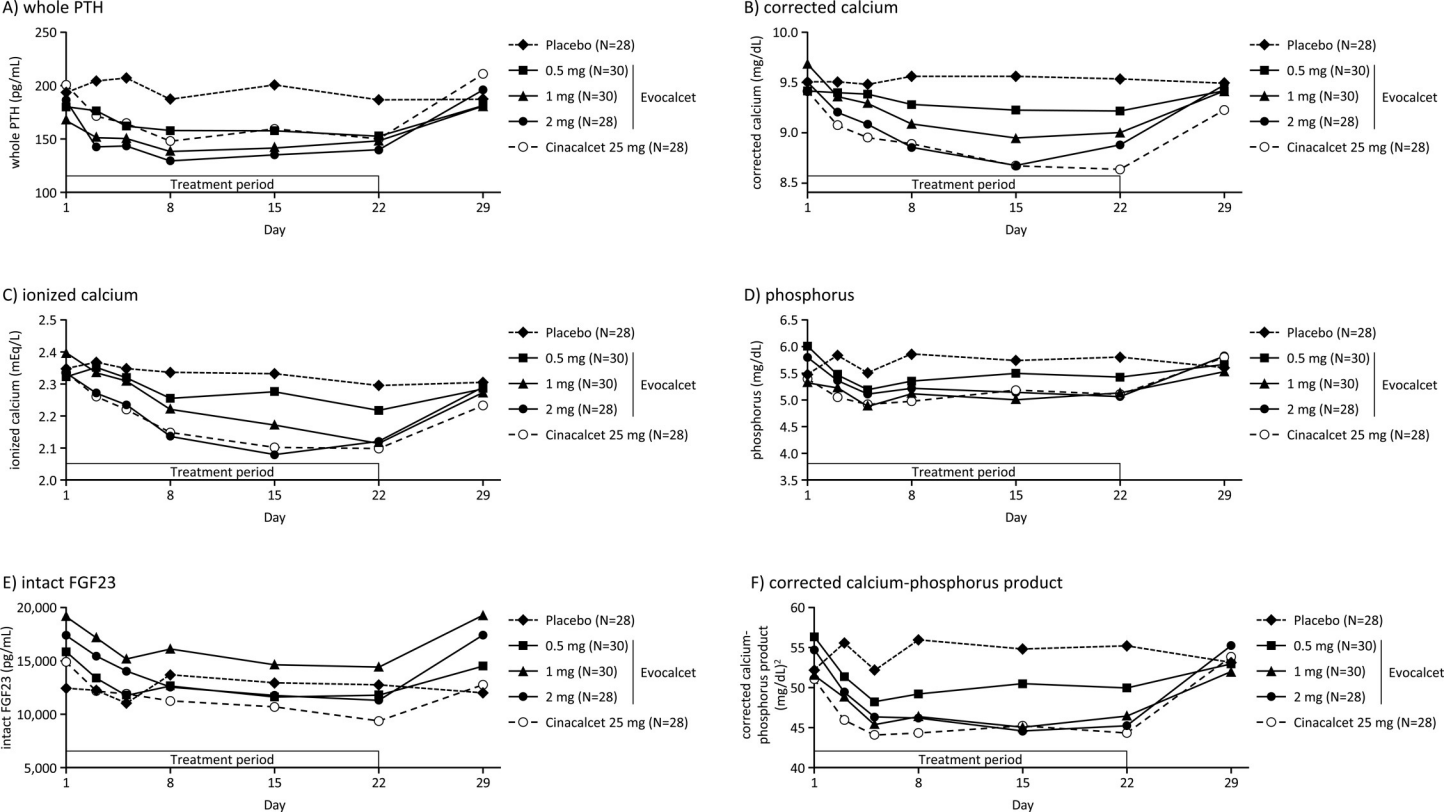
Time-course profiles of clinical laboratory variables (secondary endpoints). (A) whole PTH; (B) corrected calcium; (C) ionized calcium; (D) phosphorus; (E) intact FGF23; and (F) corrected calcium-phosphorus product. PTH, parathyroid hormone; FGF23, fibroblast growth factor 23.

The number (%) of patients who achieved the iPTH level of ≤240 pg/mL at EOT was 4 (13.3%), 11 (36.7%), 10 (35.7%), 2 (7.1%), and 11 (39.3%) in the evocalcet 0.5, 1, and 2 mg, placebo, and cinacalcet groups, respectively. The number (%) of patients who achieved a ≥30% decrease in iPTH level from baseline at EOT was 7 (23.3%), 6 (20.0%), 11 (39.3%), 2 (7.1%), and 14 (50.0%) in the evocalcet 0.5, 1, and 2 mg, placebo, and cinacalcet groups, respectively. iPTH (Fig 5), whole PTH, corrected calcium, ionized calcium, phosphorus, and intact FGF23, as well as corrected calcium-phosphorus product (Fig 6A–6F and supporting information Figures A-F in S1 Fig), decreased after initiation of treatment period in the evocalcet and cinacalcet groups. All of these parameters increased almost to the baseline values by Day 29.

### Safety

Among the 151 patients in the safety analysis set, AEs occurred in 11 (36%), 14 (47%), 9 (30%), 14 (47%), and 15 (50%) patients in the evocalcet 0.5, 1, and 2 mg, placebo, and cinacalcet groups, respectively (Table 3 and S2 Table).

**Table 3:** Summary of gastrointestinal adverse events and calcium decrease-related events.

	Placebo (N = 30)	Evocalcet 0.5 mg (N = 31)	Evocalcet 1 mg (N = 30)	Evocalcet 2 mg (N = 30)	Cinacalcet 25 mg (N = 30)
**Patients with any AE**	14 (46.7)	11 (35.5)	14 (46.7)	9 (30.0)	15 (50.0)
Gastrointestinal adverse events	0	1 (3.2)	1 (3.3)	2 (6.7)	1 (3.3)
nausea	0	1 (3.2)	1 (3.3)	1 (3.3)	1 (3.3)
vomiting	0	0	0	1 (3.3)	0
Calcium decrease-related events	2 (6.7)	0	0	3 (10.0)	3 (10.0)
blood calcium decreased	0	0	0	0	1 (3.3)
corrected calcium decreased	2 (6.7)	0	0	3 (10.0)	1 (3.3)
hypocalcemia	0	0	0	0	1 (3.3)

Data in the table are presented as number of patients with AEs (%). Abbreviation: AE, adverse event

No AEs led to death in this study. Serious AEs occurred in 3 patients: 2 (6.5%) in the evocalcet 0.5 mg group (cholelithiasis and shunt occlusion) and 1 (3.3%) in the cinacalcet group (thrombotic cerebral infarction). These AEs were considered moderate and unrelated to the study drug.

The number (%) of the patients who experienced GI adverse events (nausea and vomiting) and calcium decrease-related events (classed as MedDRA preferred terms “blood calcium decreased”, “corrected calcium decreased”, and “hypocalcemia”) are shown in Table 3. No differences were observed in the incidences of GI adverse events in the evocalcet 0.5, 1, and 2 mg, placebo, and cinacalcet groups. Calcium decrease-related events occurred in 3 (10.0%), 2 (6.7%), and 3 (10.0%) patients in the evocalcet 2 mg, placebo, and cinacalcet groups, respectively. All of these events were judged as mild or moderate and were resolved without any intervention.

A total of 11 patients discontinued the study, including seven patients who discontinued for corrected calcium ≤7.5 mg/dL (1, 3, 1, and 2 in the evocalcet 1 mg, 2 mg, placebo, and cinacalcet groups, respectively). The incidence of discontinuation for calcium decrease was relatively higher in evocalcet 2 mg and cinacalcet groups (Fig 1).

Regarding the results of the electrocardiographic evaluations, no clinically problematic fluctuations were observed after administration of the investigational drugs.

## Discussion

This Phase 2b study was conducted to compare the efficacy and safety of three different doses (0.5, 1, and 2 mg) of evocalcet, with placebo and cinacalcet 25 mg in Japanese HDSHPT. The dose-response profile of evocalcet was confirmed based on the mean percent changes of iPTH levels from baseline in the evocalcet 0.5, 1, 2 mg, and placebo groups based on seven types of contrast patterns, all of which showed statistically significant differences. The dose dependency of evocalcet was confirmed as well as the advantage of evocalcet over placebo, starting at an evocalcet dose of 1 mg. The present results (shown in Fig 3) also suggest that the efficacy of evocalcet at a dose of 2 mg was nearly equivalent to that of cinacalcet 25 mg.

Increased levels of FGF23 are associated with CKD progression [14]. Some observational studies of HD patients have also linked increased levels of FGF23 with early mortality and cardiovascular events [15], [16]. FGF23 decreased in all evocalcet groups and in the cinacalcet group in this study, which is consistent with previous findings of cinacalcet [17], [18] and etelcalcetide [19].

The most relevant AEs in this study were calcium decrease-related events, which occurred more frequently in the evocalcet 2 mg (n = 3 [10.0%]) and cinacalcet groups (n = 3 [10.0%]). Similar results were reported in previous studies of SHPT patients who were treated with cinacalcet [3], [7], [20].

Although no between-group differences were observed for GI AEs in this study, in the Japanese Phase 2 study of cinacalcet, the incidences of GI AEs were 10.0% (n = 3) in the 25 mg group and 32.3% (n = 10) in the 50 mg group, whereas no GI AEs occurred in the placebo group [7]. Similarly, in the Phase 3 study of cinacalcet [4], cinacalcet induced GI AEs in a dose-dependent manner. In this study, however, the development of GI AEs was considered to be suppressed by the fixed dose of cinacalcet 25 mg administered during the 3-week treatment.

Based on these findings, evocalcet 1 mg was considered appropriate as the initial dose for HDSHPT. The progression of SHPT leads to elevation of serum PTH levels and nodular hyperplasia of the parathyroid glands. HD patients with severe SHPT require additional treatment; a high level of PTH (iPTH >500 pg/mL) is one of the indications for parathyroidectomy [21]. Cinacalcet has the potential to suppress serum PTH levels even in severe HDSHPT [3], [22]. Therefore, evocalcet 2 mg may be more beneficial for these severely affected patients in terms of rapidly improving their PTH levels.

Possible limitations of this study were the relatively short treatment duration (3 weeks) and use of fixed doses of evocalcet and cinacalcet, which may have precluded us from obtaining appropriate comparative results regarding the incidences of GI AEs. We aim to evaluate this effect in the subsequent Phase 3 study. Additionally, it is possible that some patients, who developed GI symptoms while receiving treatment with cinacalcet before this study were purposely omitted from enrollment. As the initial dose of cinacalcet approved in Japan is 25 mg, we established this as the fixed dose in the control group. However, this may be considered a low cinacalcet dose given that the approved initial dose in the US and Europe is 30 mg, which may have precluded us from obtaining appropriate comparative results regarding efficacy. Further, some demographic and clinical characteristics, including male sex, age greater than 65 years, dialysis vintage, use of vitamin D, use of phosphate binders, and phosphate levels, were unevenly distributed at baseline in the five study protocol arms. This may have led to biased results. Another limitation of this study is the inclusion of Japanese patients only, which limits the generalizability of the present results. The strength of this study was its design, by which different doses of evocalcet were compared not only with placebo, but also with cinacalcet as the comparator (open-label). Although the cinacalcet group was set as an open-label control group in the present study, any potential biases arising from the open-label administration of cinacalcet treatment were limited because iPTH and corrected calcium are objective markers.

In conclusion, we confirmed the dose response and the safety of all administered doses of evocalcet, as well as the efficacy of ≥1 mg evocalcet. Therefore, evocalcet at a dose of 1 mg is considered appropriate as an initial dose for treating HDSHPT. Based on the present efficacy results, evocalcet at a dose of 2 mg may be beneficial for severe HDSHPT, but this requires confirmation in a future study. Furthermore, additional head-to-head comparative studies are needed to further compare evocalcet and cinacalcet in terms of GI symptoms.

## Supporting information

S1 Checklist CONSORT checklist. (DOCX)

S1 Text Detailed exclusion criteria. (DOCX)

S2 Text Details of procedures applied to ensure blinding. (DOCX)

S3 Text Data set definitions. (DOCX)

S1 File Programming code for statistical analysis. (TXT)

S1 Fig Time-course profiles of percent changes from baseline in clinical laboratory variables (secondary endpoints). (A) whole PTH; (B) corrected calcium; (C) ionized calcium; (D) phosphorus; (E) intact FGF23; and (F) corrected calcium-phosphorus product.
(DOCX)

S1 Table List of participating institutions. (DOCX)

S2 Table Adverse events by System Organ Class and Preferred Term. (DOCX)

S1 Protocol Trial protocol. (DOCX)
