# Supplementary material for: Phase 2b study of evocalcet (KHK7580), a novel calcimimetic, in Japanese patients with secondary hyperparathyroidism undergoing hemodialysis: A randomized, double-blind, placebo-controlled, dose-finding study
Source: PLoS One. 2018 Oct 31;13(10):e0204896. doi: 10.1371/journal.pone.0204896 (PMC6209414; doi:10.1371/journal.pone.0204896)
Supplement: S1 Text — (DOCX) [file pone.0204896.s002.docx]

## S1 Text. Detailed exclusion criteria

The complete exclusion criteria were as follows:

1. Patients who used cinacalcet hydrochloride within 2 weeks before screening
2. Patients with changes in the dose and mode of administration of or newly receiving treatment with active vitamin D and its derivatives, phosphate binders, and calcium preparations within 2 weeks before screening
3. Patients with changes in the dialysis condition (dialysate calcium level, dialyzer, prescribed dialysis time, prescribed frequency of dialysis per week) within 2 weeks before screening
4. Patients who used bisphosphonates, denosumab, or teriparatide preparations within 24 weeks before screening
5. Patients who underwent parathyroidectomy or parathyroid intervention within 24 weeks before screening
6. Patients with concurrent severe heart disease (e.g., Class III or IV on the New York Heart Association Functional Classification)
7. Patients with severe hepatic function disorder (e.g., aspartate aminotransferase or alanine aminotransferase of ≥100 IU/L during screening)
8. Patients with poorly controlled hypertension or diabetes mellitus
9. Pregnant or lactating women or patients who may possibly be pregnant (i.e., those who tested positive on a pregnancy test or those who have not received a pregnancy test and have not used contraception), or patients who were unwilling to use appropriate contraception under a physician’s guidance (however, patients who did not present menstruation for ≥12 months since the last menstruation without any medical reason were considered as having non-childbearing potential)
10. Patients with a history of serious drug allergy or patients with a history of or concurrent drug addiction or alcoholism
11. Patients with a history of drug allergy to cinacalcet hydrochloride
12. Patients with a history of diagnosis and treatment of malignant tumor within 5 years before screening (excluding patients with basal cell carcinoma or surgically resected cervical cancer in situ)
13. Patients participated in a study of a drug or medical device or a similar study and have received treatment with an investigational product or an unapproved medical device within 12 weeks before screening
14. Patients who previously received evocalcet
15. Patients with primary hyperparathyroidism
16. Other than the above criteria, patients whose participation in this study was judged as unacceptable by the investigators.
