# Supplementary material for: Phase 2b study of evocalcet (KHK7580), a novel calcimimetic, in Japanese patients with secondary hyperparathyroidism undergoing hemodialysis: A randomized, double-blind, placebo-controlled, dose-finding study
Source: PLoS One. 2018 Oct 31;13(10):e0204896. doi: 10.1371/journal.pone.0204896 (PMC6209414; doi:10.1371/journal.pone.0204896)
Supplement: S2 Text — (DOCX) [file pone.0204896.s003.docx]

**S2 Text.** **Details of procedures applied to ensure blinding**

After individual subjects completed treatment, the investigational drug administrator of the clinical trial or medical institution confirmed the residual investigational drug quantity. The remaining investigational drugs were sealed and collected by the sponsor or investigator. To ensure the blinding after sealing by the sponsors, the recovered investigational drugs were stored and managed until the data were unlocked.

Upon opening the sealed original layout book, the investigation allocation manager confirmed that the investigator’s emergency key and that of the investigation allocation manager were both unopened. Additionally, after opening the allocation register, the concentration of evocalcet in plasma was measured, and the sponsor received the results corresponding to the plasma drug concentration measurement result from the corresponding laboratory. Thus, during the trial period, the blinding of the investigational product was secured.
