# Supplementary material for: Phase 2b study of evocalcet (KHK7580), a novel calcimimetic, in Japanese patients with secondary hyperparathyroidism undergoing hemodialysis: A randomized, double-blind, placebo-controlled, dose-finding study
Source: PLoS One. 2018 Oct 31;13(10):e0204896. doi: 10.1371/journal.pone.0204896 (PMC6209414; doi:10.1371/journal.pone.0204896)
Supplement: S3 Text — (DOCX) [file pone.0204896.s004.docx]

**S3 Text.** Data set definitions

The full analysis set (FAS) was defined as all enrolled patients, except those who were not randomized, did not receive any study treatment dose, or had no intact parathyroid hormone level measurement at baseline or after the start of study treatment. The per protocol set (PPS) was defined as all patients in the FAS, except those who did not fulfill the inclusion criteria or met any of the exclusion criteria, had drug compliance <70% at the end of study treatment, or had an important protocol deviation that might affect the efficacy evaluation. The PPS was used for the main efficacy analyses. The safety analysis set was defined as all enrolled patients, except those who did not receive any study treatment dose.
