# Supplementary material for: Phase 2b study of evocalcet (KHK7580), a novel calcimimetic, in Japanese patients with secondary hyperparathyroidism undergoing hemodialysis: A randomized, double-blind, placebo-controlled, dose-finding study
Source: PLoS One. 2018 Oct 31;13(10):e0204896. doi: 10.1371/journal.pone.0204896 (PMC6209414; doi:10.1371/journal.pone.0204896)

**S1 Fig. Time-course profiles of percent changes from baseline in clinical laboratory variables (secondary endpoints).**

(A) whole PTH; (B) corrected calcium; (C) ionized calcium; (D) phosphorus; (E) intact FGF23; and (F) corrected calcium-phosphorus product

PTH, parathyroid hormone; FGF23, fibroblast growth factor 23


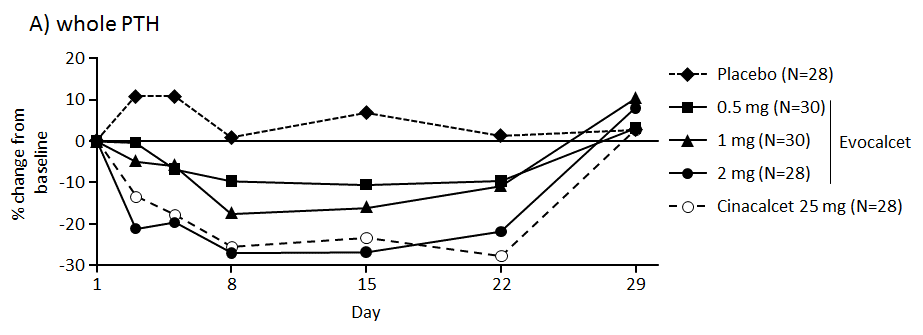


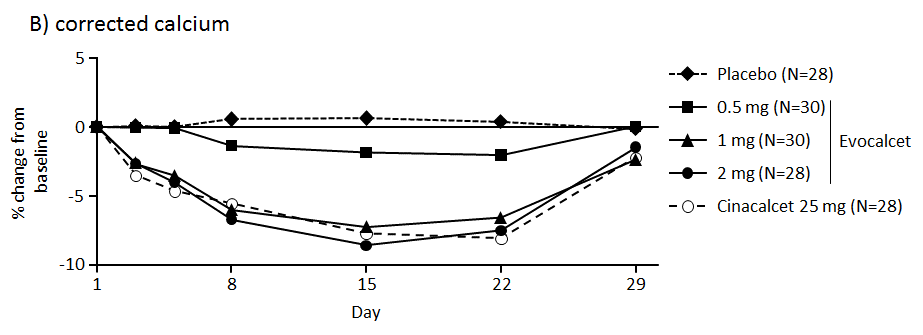


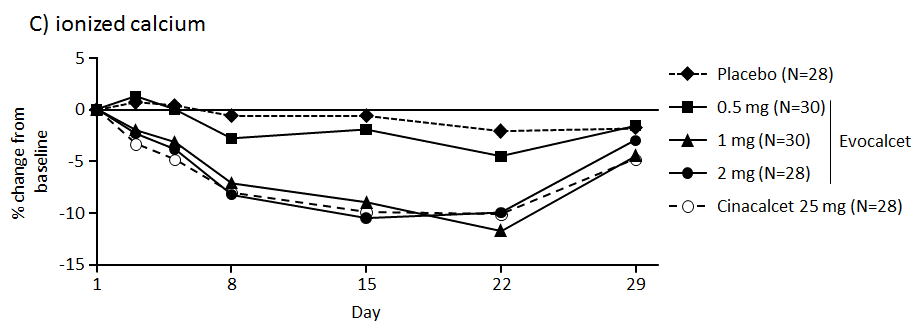


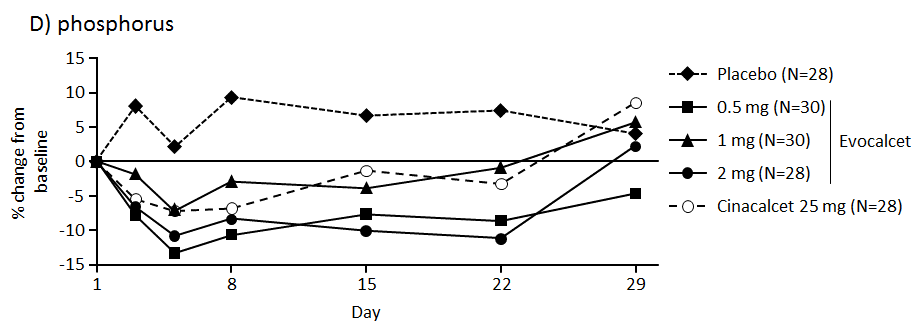


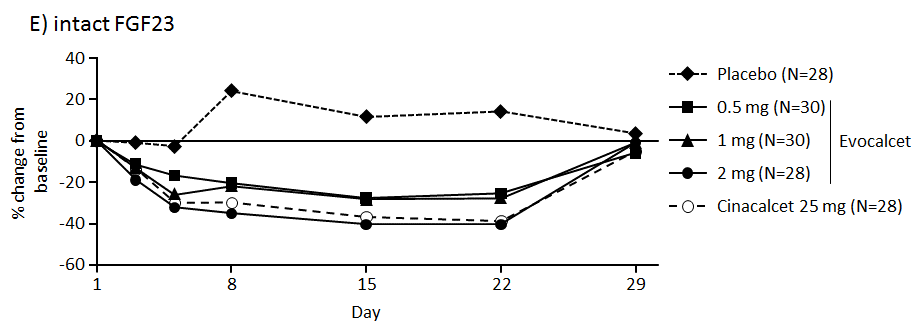


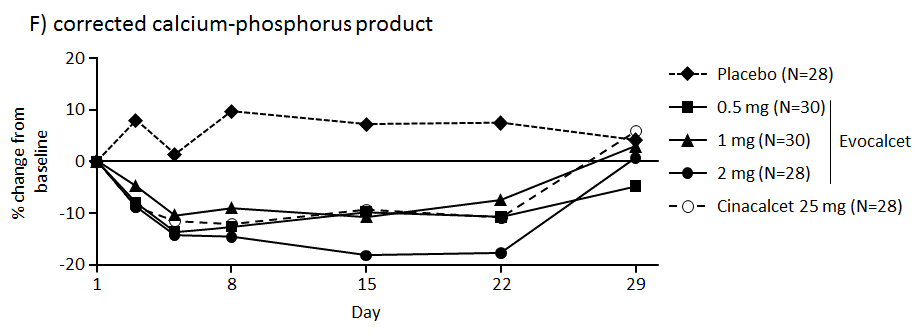

Supplement: S1 Fig — (A) whole PTH; (B) corrected calcium; (C) ionized calcium; (D) phosphorus; (E) intact FGF23; and (F) corrected calcium-phosphorus product. (DOCX) [file pone.0204896.s006.docx]
