# Supplementary material for: Phase 2b study of evocalcet (KHK7580), a novel calcimimetic, in Japanese patients with secondary hyperparathyroidism undergoing hemodialysis: A randomized, double-blind, placebo-controlled, dose-finding study
Source: PLoS One. 2018 Oct 31;13(10):e0204896. doi: 10.1371/journal.pone.0204896 (PMC6209414; doi:10.1371/journal.pone.0204896)
Supplement: S1 Table — (DOCX) [file pone.0204896.s007.docx]

**S1 Table. List of participating institutions**

| **Principal Investigator** | **Institution** | **Type of**  **Review Board** | **Name of External Review Board** |
| --- | --- | --- | --- |
| Noritomo Itami | Higashimuroran Satellite Clinic | External | Medical Corporation Bokoi Nikko Memorial Hospital IRB |
| Toshinobu Sato | Japan Community Health Care Organization Sendai Hospital | Internal |  |
| Itaru Ebihara | Mito Saiseikai General Hospital | External | Review Board of Human Rights and Ethics for Clinical Studies |
| Jun Niwayama | Sumiyoshi Clinic Hospital | External | Review Board of Human Rights and Ethics for Clinical Studies |
| Takayuki Toda | Tsuchiura Kyodo General Hospital | Internal |  |
| Yoshitaka Maeda | JA Toride Medical Center | External | Review Board of Human Rights and Ethics for Clinical Studies |
| Tadashi Iitsuka | Ibaraki Seinan Medical Center Hospital | External | Review Board of Human Rights and Ethics for Clinical Studies |
| Haruyuki Ogura | Kurosawa Hospital | External | Review Board of Human Rights and Ethics for Clinical Studies |
| Kazue Ueki | Toho Hospital | External | Review Board of Human Rights and Ethics for Clinical Studies |
| Johji Ohshima | Kubojima Clinic | External | Koukeikai Sugiura Clinic IRB |
| Yoshihiro Miyauchi | Asahi General Hospital | Internal |  |
| Hiroyuki Tamura | Toshima Chuou Hospital | External | Kaikoukai Healthcare Corporation Nagoya Kyoritsu Hospital IRB |
| Kenichi Oguchi | Ikegami General Hospital | External | Medical Corporation Showakai IRB |
| Eriko Kinugasa | Showa University Northern Yokohama Hospital | Internal |  |
| Shigeru Miyazaki | Shinrakuen Hospital | Internal |  |
| Ryoichi Miyazaki | Fujita Memorial Hospital | External | Fukui General Hospital IRB |
| Yutaka Kanno | Kanno Dialysis & Vascular Access Clinic | External | Review Board of Human Rights and Ethics for Clinical Studies |
| Hiroaki Shimosaka | Tajimi Clinic | External | Review Board of Human Rights and Ethics for Clinical Studies |
| Hisanori Azekura | Sanaru Sun-Clinic | External | Review Board of Human Rights and Ethics for Clinical Studies |
| Masanobu Horie | Daiyukaidaiichi Hospital | Internal |  |
| Yoshinari Tsuruta | Meiyo Clinic Hemodialysis Center | External | Review Board of Human Rights and Ethics for Clinical Studies |
| Hirotake Kasuga | Kaikoukai Central Clinic | External | Kaikoukai Healthcare Corporation Nagoya Kyoritsu Hospital IRB |
| Hajime Inoue | Ama Kyoritsu Clinic | External | Kaikoukai Healthcare Corporation Nagoya Kyoritsu Hospital IRB |
| Hiroshi Ogawa | Sinseikai Dai-Ichi Hospital | External | Review Board of Human Rights and Ethics for Clinical Studies |
| Morihiro Kondo | Rakuwakai Otowa Memorial Hospital | External | Rakuwakai Otowa Hospital IRB |
| Yoshihiro Tsujimoto | Inoue Hospital | Internal |  |
| Shigeichi Shoji | Shirasagi Hospital | External | Review Board of Human Rights and Ethics for Clinical Studies |
| Mitsuru Yoshimoto | Ohno Memorial Hospital | Internal |  |
| Akira Fujimori | Konan Hospital | Internal |  |
| Masaki Fukushima | Shigei Medical Research Hospital | Internal |  |
| Misaki Moriishi | Nakajima Tsuchiya Clinic | External | Medical Corporation Akane Tsuchiya General Hospital IRB |
| Zenzou Fujii | St. Hill Hospital | External | Koukeikai Sugiura Clinic IRB |
| Jun Minakuchi | Kawashima Hospital | Internal |  |
| Seiichi Obayashi | Kinashi Obayashi Hospital | External | Koukeikai Sugiura Clinic IRB |
| Yuzuru Sato | Sato Cardiologic Clinic | External | Koukeikai Sugiura Clinic IRB |
| Kenji Yuasa | Kochi Takasu Hospital | External | Koukeikai Sugiura Clinic IRB |
| Hiroshi Kimura | Fukuoka Renal Clinic | External | Medical Corporation Houmankai Umezu Clinic IRB |
| Satoshi Funakoshi | Nagasaki Kidney Center | External | Kouseikai Hospital IRB |
| Manei Oku | Seijinkai Ikeda Hospital | Internal |  |
| Masayuki Okazaki | Jyoban Hospital | External | Koyasu Neurosurgical Clinic IRB |

Abbreviation: IRB, institutional review board
