# Supplementary material for: Phase 2b study of evocalcet (KHK7580), a novel calcimimetic, in Japanese patients with secondary hyperparathyroidism undergoing hemodialysis: A randomized, double-blind, placebo-controlled, dose-finding study
Source: PLoS One. 2018 Oct 31;13(10):e0204896. doi: 10.1371/journal.pone.0204896 (PMC6209414; doi:10.1371/journal.pone.0204896)
Supplement: S2 Table — (DOCX) [file pone.0204896.s008.docx]

**S2 Table. Adverse events by System Organ Class and Preferred Term**

| **[System Organ Class]**  **Preferred Term** | **Placebo**  **(N=30)** | **Evocalcet**  **0.5 mg**  **(N=31)** | **Evocalcet**  **1 mg**  **(N=30)** | **Evocalcet**  **2 mg**  **(N=30)** | **Cinacalcet 25 mg**  **(N=30)** |
| --- | --- | --- | --- | --- | --- |
| **Patients with any AE** | 14 (46.7) | 11 (35.5) | 14 (46.7) | 9 (30.0) | 15 (50.0) |
| [Blood and lymphatic system disorders] | 1 (3.3) | 0 | 0 | 0 | 0 |
| Renal anemia | 1 (3.3) | 0 | 0 | 0 | 0 |
| [Cardiac disorders] | 1 (3.3) | 0 | 0 | 0 | 0 |
| Palpitations | 1 (3.3) | 0 | 0 | 0 | 0 |
| [Eye disorders] | 0 | 1 (3.2) | 0 | 0 | 1 (3.3) |
| Conjunctival deposit | 0 | 0 | 0 | 0 | 1 (3.3) |
| Conjunctivitis allergic | 0 | 0 | 0 | 0 | 1 (3.3) |
| Scintillating scotoma | 0 | 1 (3.2) | 0 | 0 | 0 |
| [Gastrointestinal disorders] | 0 | 1 (3.2) | 1 (3.3) | 3 (10.0) | 3 (10.0) |
| Nausea | 0 | 1 (3.2) | 1 (3.3) | 1 (3.3) | 1 (3.3) |
| Constipation | 0 | 0 | 0 | 0 | 1 (3.3) |
| Periodontal disease | 0 | 0 | 0 | 1 (3.3) | 0 |
| Toothache | 0 | 0 | 0 | 0 | 1 (3.3) |
| Vomiting | 0 | 0 | 0 | 1 (3.3) | 0 |
| [General disorders and administration site conditions] | 0 | 0 | 0 | 1 (3.3) | 1 (3.3) |
| Malaise | 0 | 0 | 0 | 1 (3.3) | 1 (3.3) |
| [Hepatobiliary disorders] | 0 | 1 (3.2) | 0 | 0 | 0 |
| Cholelithiasis | 0 | 1 (3.2) | 0 | 0 | 0 |
| [Infections and infestations] | 5 (16.7) | 5 (16.1) | 5 (16.7) | 2 (6.7) | 6 (20.0) |
| Nasopharyngitis | 4 (13.3) | 5 (16.1) | 5 (16.7) | 2 (6.7) | 5 (16.7) |
| Acute sinusitis | 0 | 0 | 0 | 0 | 1 (3.3) |
| Otitis media chronic | 0 | 0 | 0 | 0 | 1 (3.3) |
| Shunt infection | 1 (3.3) | 0 | 0 | 0 | 0 |
| Infected dermal cyst | 0 | 0 | 1 (3.3) | 0 | 0 |
| [Injury, poisoning and procedural complications] | 1 (3.3) | 1 (3.2) | 2 (6.7) | 0 | 1 (3.3) |
| Shunt occlusion | 0 | 1 (3.2) | 0 | 0 | 0 |
| Contusion | 1 (3.3) | 0 | 0 | 0 | 0 |
| Wound | 0 | 0 | 1 (3.3) | 0 | 0 |
| Shunt stenosis | 0 | 0 | 1 (3.3) | 0 | 0 |
| Procedural hypotension | 0 | 0 | 0 | 0 | 1 (3.3) |
| [Investigations] | 3 (10.0) | 0 | 2 (6.7) | 3 (10.0) | 2 (6.7) |
| Corrected calcium decreased | 2 (6.7) | 0 | 0 | 3 (10.0) | 1 (3.3) |
| Blood calcium decreased | 0 | 0 | 0 | 0 | 1 (3.3) |
| Blood triglycerides increased | 1 (3.3) | 0 | 0 | 0 | 0 |
| White blood cell count increased | 0 | 0 | 1 (3.3) | 0 | 0 |
| Electrocardiogram ST-T change | 0 | 0 | 1 (3.3) | 0 | 0 |
| [Metabolism and nutrition disorders] | 1 (3.3) | 0 | 0 | 0 | 1 (3.3) |
| Hyperkalaemia | 1 (3.3) | 0 | 0 | 0 | 0 |
| Hypocalcaemia | 0 | 0 | 0 | 0 | 1 (3.3) |
| [Musculoskeletal and connective tissue disorders] | 1 (3.3) | 1 (3.2) | 0 | 0 | 1 (3.3) |
| Arthralgia | 1 (3.3) | 0 | 0 | 0 | 0 |
| Muscle spasms | 0 | 1 (3.2) | 0 | 0 | 0 |
| Pain in extremity | 0 | 0 | 0 | 0 | 1 (3.3) |
| [Nervous system disorders] | 0 | 0 | 0 | 0 | 1 (3.3) |
| Thrombotic cerebral infarction | 0 | 0 | 0 | 0 | 1 (3.3) |
| [Renal and urinary disorders] | 1 (3.3) | 0 | 0 | 0 | 0 |
| Haematuria | 1 (3.3) | 0 | 0 | 0 | 0 |
| [Respiratory, thoracic and mediastinal disorders] | 0 | 1 (3.2) | 1 (3.3) | 0 | 1 (3.3) |
| Cough | 0 | 0 | 0 | 0 | 1 (3.3) |
| Upper respiratory tract inflammation | 0 | 0 | 1 (3.3) | 0 | 0 |
| Oropharyngeal pain | 0 | 1 (3.2) | 0 | 0 | 0 |
| [Skin and subcutaneous tissue  disorders] | 2 (6.7) | 0 | 4 (13.3) | 0 | 3 (10.0) |
| Eczema | 0 | 0 | 1 (3.3) | 0 | 1 (3.3) |
| Dermal cyst | 0 | 0 | 1 (3.3) | 0 | 0 |
| Dermatitis | 0 | 0 | 0 | 0 | 1 (3.3) |
| Eczema asteatotic | 0 | 0 | 1 (3.3) | 0 | 0 |
| Erythema | 0 | 0 | 0 | 0 | 1 (3.3) |
| Pruritus | 1 (3.3) | 0 | 0 | 0 | 0 |
| Skin exfoliation | 1 (3.3) | 0 | 0 | 0 | 0 |
| Pruritus generalised | 0 | 0 | 1 (3.3) | 0 | 0 |

Data in the table are presented as number of patients with AEs (%). Abbreviation: AE, adverse event
