# Supplementary material for: Phase 2b study of evocalcet (KHK7580), a novel calcimimetic, in Japanese patients with secondary hyperparathyroidism undergoing hemodialysis: A randomized, double-blind, placebo-controlled, dose-finding study
Source: PLoS One. 2018 Oct 31;13(10):e0204896. doi: 10.1371/journal.pone.0204896 (PMC6209414; doi:10.1371/journal.pone.0204896)
Supplement: S1 Protocol — (DOCX) [file pone.0204896.s009.docx]

**S1 Protocol. Trial protocol**

**A Phase 2 Study of KHK7580**

**(Double-blind, Parallel-Group, Dose-Response Study of KHK7580 in Patients with Secondary Hyperparathyroidism Receiving Hemodialysis)**

**Protocol**

**Kyowa Hakko Kirin Co., Ltd.**

Protocol Number: 7580-005

Version Number: 1.0

Preparation date: July 10, 2014

HANDLING OF THIS PROTOCOL

The information in this protocol is the property of Kyowa Hakko Kirin Co., Ltd., the sponsor of this study, and is provided only to those who are directly involved in this study such as the investigator and subinvestigator (including staff such as the trial collaborator), head of the medical institution, study drug manager, clinical trial secretariat, and IRB.

Therefore, it is requested not to disclose or divulge the information in this protocol to a third party not involved in this study.

Please also acknowledge that the information in this protocol cannot be copied, quoted, or published without the permission of Kyowa Hakko Kirin Co., Ltd.

LIST OF ABBREVIATIONS AND DEFINITION OF TERMS

**Abbreviation**

| Abbreviation | Expanded Form |
| --- | --- |
| CKD | Chronic kidney disease |
| CKD-MBD | CKD-Mineral and Bone Disorder |
| CYP | Cytochrome P450 |
| DOPPS | Dialysis Outcomes and Practice Patterns Study |
| EDC | Electronic Data Capture |
| FAS | Full Analysis Set |
| FGF | Fibroblast Growth Factor |
| PPS | Per Protocol Set |
| PTH | Parathyroid hormone |
| PTx | Parathyroidectomy |
| SHPT | Secondary hyperparathyroidism |

**Definitions of Terms**

| Term | Term Definition and Explanation |
| --- | --- |
| Study 7580-001 | A phase I study of KHK7580 in healthy adult male subjects (Study Number: 7580-001) |
| Study 7580-002 | A phase I study of KHK7580 in healthy adult male subjects (multiple-dose study) (Study Number: 7580-002) |
| Study 7580-003 | A Phase 1/2 study of KHK7580 (single- and multiple-dose, intra-subject dose-escalation study of KHK7580 in subjects with secondary hyperparathyroidism receiving hemodialysis) (Study Number: 7580-003) |
| Alb | Albumin |
| ALP | Alkaline phosphatase |
| ALT | Alanine aminotransferase |
| AST | Aspartate aminotransferase |
| AUC_0-∞_ | Area under the plasma drug concentration-time curve from 0 to infinity |
| AUC_0-t_ | Area under the plasma drug concentration-time curve from 0 to time t after drug administration |
| BAP | Bone alkaline phosphatase |
| ChE | Cholinesterase |
| CK | Creatine kinase |
| C_max_ | Highest plasma drug concentration |
| DNA | Deoxyribonucleic acid |
| EDTA-2K | Ethylenediaminetetraacetic acid dipotassium salt |
| EDTA-2Na | Ethylenediaminetetraacetic acid disodium salt |
| F | Bioavailability |
| GCP | Good Clinical Practice |
| γ-GTP | Gamma-glutamyl transpeptidase |
| IRB | Institutional Review Board |
| Study KRN1493/03-A06 | A randomized, double-blind, parallel-group, dose-response study of KRN1493 in hemodialysis patients with secondary hyperparathyroidism (Phase 2) |
| LC/MS/MS | Liquid chromatography/tandem mass spectrometry |
| LDH | Lactate dehydrogenase |
| MedDRA/J | Medical Dictionary for Regulatory Activities/Japanese Edition |
| P1NP | Procollagen type 1 N-terminal propeptide |
| PR interval | The time elapsing between the beginning of the P wave and the beginning of the QRS complex in the electrocardiogram. |
| PT | MedDRA/J Preferred Term |
| QOL | Quality of Life |
| QRS interval | The time elapsing between the beginning of the Q wave and the end of the S wave in the electrocardiogram. |
| QT interval | The time elapsing between the beginning of the Q wave and the end of the T wave in the electrocardiogram. |
| QTc interval | QT interval corrected for heart rate using the Bazett formula (QTcB), or QT interval corrected for heart rate using the Fridericia formula (QTcF) |
| RNA | Ribonucleic acid |
| RR interval | The time elapsing between one R wave and the next R wave in the  electrocardiogram. |
| SOC | MedDRA/J System Organ Class |
| t1/2 | Plasma elimination half-life |
| TRACP-5b | Tartrate-resistant acid phosphatase 5b |
| Adverse Reaction | Any adverse event for which the causal relationship to the investigational product was assessed as “related” or “unknown.” |

Study period

The study period for each subject is defined as the period from the acquisition date of informed consent to the stipulated final assessments or assessments upon discontinuation.

Investigational product administration start date, time since administration

Investigational product administration start date was Day 1. The time elapsed after investigational product administration was described as X hours after investigational product administration.

End of investigational product administration

Day 22 or upon discontinuation prior to Day 22.

SUMMARY OF STUDY PLAN

I Objectives

The objective of this study was to evaluate the efficacy and safety of 0.5, 1, or 2 mg KHK7580 or placebo, or 25 mg KRN1493 (cinacalcet hydrochloride) orally administered to subjects with secondary hyperparathyroidism (SHPT) receiving hemodialysis (HD) once daily for 3 weeks in a multicenter, randomized, placebo-controlled, double-blind, parallel-group, dose-response study (including the KRN1493 group as an open-control group).

II Study Phase

Phase 2

III Criteria for Evaluation

1) Efficacy Evaluation

Primary Endpoint:

• Percent change in intact PTH level from baseline at the end of study treatment

Secondary Endpoints:

• Number and percentage of subjects achieving a percent decrease in intact PTH level of ≥30% (percent change ≤−30%) at the end of study treatment

• Number and percentage of subjects achieving an intact PTH level of ≤240 pg/mL at the end of study treatment

• Intact PTH level, whole PTH level, corrected serum Ca level, ionized Ca level, serum P level, intact fibroblast growth factor-23 (FGF23) level, and corrected serum Ca-P product at each time point and at the end of study treatment

Exploratory Endpoints:

• Bone metabolic markers at the end of study treatment and at the end of the follow-up period

• Parathyroid gland (volume and blood flow) at the end of study treatment

2) Safety Evaluation

• Treatment-emergent adverse events (TEAEs)

• Lowest corrected serum Ca level between the start of study treatment and the end of the follow-up period

• Laboratory values

• Vital signs

• 12-lead electrocardiogram

• Ophthalmological examination

3) Pharmacokinetic Evaluation

• Plasma KHK7580 concentration

IV Subjects (Disease)

SHPT patients receiving hemodialysis

V Inclusion Criteria

Subjects eligible for enrollment in the study had to meet all of the following criteria:

1) Personally submitted written voluntary informed consent to participate in the study

2) Aged ≥20 and <75 years old at the time of acquisition of consent

3) Stable chronic renal failure treated with hemodialysis 3 times weekly for at least 12 weeks before screening

4) Intact PTH level of ≥240 pg/mL at screening

5) Corrected serum Ca level of ≥8.4 mg/dL at screening

VI Exclusion Criteria

Subjects were excluded from participating in this study if they met any of the following criteria.

1) Use of cinacalcet hydrochloride within 2 weeks before screening

2) Change in dose or dosing regimen of an active vitamin D drug or its derivatives, phosphate binder, or calcium preparation within 2 weeks before screening; or start of treatment with such drugs within 2 weeks before screening

3) Change in prescribed conditions of dialysis (dialysate Ca concentration, dialyzer, prescribed dialysis time, and prescribed number of dialysis per week) within 2 weeks before screening

4) Treatment with bisphosphonates, denosumab, or teriparatide within 24 weeks before screening

5) Parathyroidectomy (PTx) and/or parathyroid intervention within 24 weeks before screening

6) Severe heart disease (e.g. ≥Class III based on New York Heart Association classification, protocol (supplement), Attachment 5)

7) Severe hepatic dysfunction (e.g. AST or ALT of ≥100 IU/L at screening)

8) Uncontrolled hypertension and/or diabetes mellitus

9) Pregnant, lactating, and possibly pregnant (positive pregnancy test or no pregnancy test performed and not using contraception), or unwilling to use an appropriate method of contraception as instructed by a physician; Amenorrhea for ≥12 months after the last menstrual period without an alternative medical cause is considered to be non-childbearing potential.

10) History of serious drug allergy; history or complications of drug or alcohol poisoning

11) History of drug allergy to cinacalcet hydrochloride

12) History of diagnosis and treatment of malignant tumor within 5 years before screening (excluding basal cell carcinoma or surgically resected intraepithelial carcinoma of uterine cervix)

13) Participation in a clinical study or equivalent study of a pharmaceutical product or medical device, and use of an investigational product or unapproved medical device within 12 weeks prior to screening

14) Prior treatment with KHK7580

15) Hyperparathyroidism primary

16) Other conditions unfit for participation in this study at discretion of the investigator or subinvestigator.

VII Target Number of Subjects

150 subjects (30 subjects in each group) as subjects for investigational product administration

VIII Investigational Product

1) Test Drug

Company code number: KHK7580

Generic name: Not set

Chemical name: (4-{(3S) -3-[(1R) -1-(Naphthalen-1-yl) ethylamino] pyrrolidin-1-yl} phenyl) acetic acid

Molecular formula: C_24_H_26_N_2_O_2_

Molecular weight: 374.48

Content and dosage form:

KHK7580 0.5 mg tablets: A pale orange, film-coated tablet containing 0.5 mg of KHK7580

KHK7580 1 mg tablet: A pale orange film-coated tablet containing 1 mg of KHK7580

KHK7580 2 mg tablet: A pale orange film-coated tablet containing 2 mg of KHK7580

2) Control drug-1: test drug (KHK7580) placebo

A tablet that is indistinguishable from KHK7580 0.5 mg tablets, 1mg tablets, and 2 mg tablets, and contains no KHK7580

3) Control drug-2: KRN1493

A pale yellow to yellowish-green film-coated tablet containing 27.55 mg of cinacalcet hydrochloride (25 mg as cinacalcet)

IX Study Design

Subjects who have consented to participation in the study will undergo screening prior to the start of dialysis on the dialysis date after the longest interval between dialysis sessions. Subjects who satisfy all the inclusion criteria will be enrolled prior to investigational product administration on Day 1 and then randomly and evenly assigned to the KHK7580 0.5, 1, or 2 mg, or placebo group, or the KRN1493 25 mg group using a dynamic allocation method. Administration of the investigational product will be started after assignment to each group on Day 1 and continued every day for 3 weeks. Assessments at the end of administration will be performed on Day 22, and there is then a follow-up period followed by final assessments on Day 29.


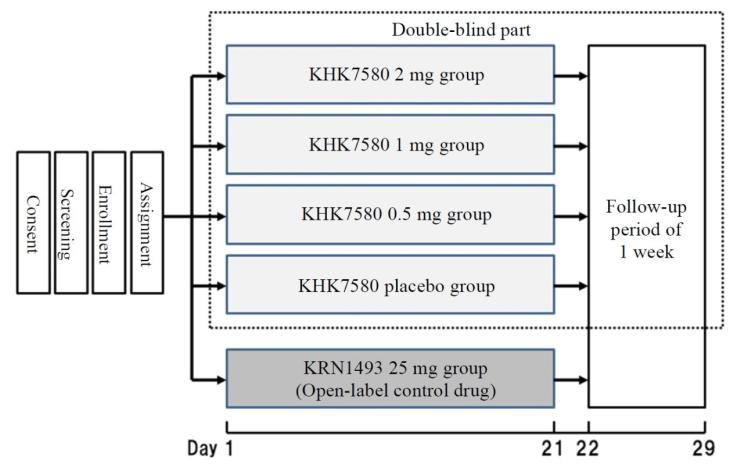


X Dosage, Dosing Regimen and Treatment Period

KHK7580 (0.5 mg, 1 mg, 2 mg, or placebo) or KRN1493 (25 mg) will be orally administered once daily for three weeks. Treatment with the study drug will be started immediately before dialysis after the longest interval between dialysis sessions. The time of administration of the study drug from the day after starting treatment will be approximately the same time (may be administered after the start of dialysis) on the day of starting administration.

XI Scheduled Study Period

July 2014 to June 2015

Tale of Contents

[HANDLING OF THIS PROTOCOL 2](#_Toc497833016)

[LIST OF ABBREVIATIONS AND DEFINITION OF TERMS 3](#_Toc497833017)

[SUMMARY OF STUDY PLAN 5](#_Toc497833018)

[Tale of Contents 9](#_Toc497833019)

[1 History and Background of Study Plan 15](#_Toc497833020)

[1.1 Background 15](#_Toc497833021)

[1.2 Explanation of the Study Drug 16](#_Toc497833022)

[1.3 Results of Non-Clinical Studies 16](#_Toc497833023)

[1.3.1 Pharmacological study 16](#_Toc497833024)

[1.3.2 Pharmacokinetics and drug metabolism 17](#_Toc497833025)

[1.3.3 Toxicity 18](#_Toc497833026)

[1.3.3.1 Multiple-dose toxicity studies 18](#_Toc497833027)

[1.3.3.2 Genotoxicity and reproductive/developmental toxicity studies 19](#_Toc497833028)

[1.4 Results of Clinical Studies 19](#_Toc497833029)

[1.4.1 Single-dose phase I study in healthy adult male subjects (Study 7580-001) 19](#_Toc497833030)

[1.4.2 Multiple-dose phase I study in healthy adult male subjects (Study 7580-002) 20](#_Toc497833031)

[1.4.3 Single-dose and multiple-dose phase I/II study in secondary hyperparathyroidism patients receiving chronic hemodialysis (Study 7580-003) 20](#_Toc497833032)

[1.5 Background of Preparation of This Protocol 21](#_Toc497833033)

[2 GCP Compliance Statement 21](#_Toc497833034)

[3 Objective of This Study 21](#_Toc497833035)

[4 Study Design 22](#_Toc497833036)

[4.1 Study Design 22](#_Toc497833037)

[4.2 Type of This Study 22](#_Toc497833038)

[4.3 Endpoints 22](#_Toc497833039)

[4.3.1 Efficacy evaluation 22](#_Toc497833040)

[4.3.1.1 Primary endpoint 22](#_Toc497833041)

[4.3.1.2 Secondary endpoints 23](#_Toc497833042)

[4.3.2 Safety evaluation 23](#_Toc497833043)

[4.3.3 Pharmacokinetic evaluation 23](#_Toc497833044)

[4.4 Target Number of Subjects 23](#_Toc497833045)

[4.5 Randomization and Blinding 23](#_Toc497833046)

[4.5.1 Randomization 23](#_Toc497833047)

[4.5.1.1 Randomization method 23](#_Toc497833048)

[4.5.1.2 Timing of randomization 23](#_Toc497833049)

[4.5.2 Blinding 24](#_Toc497833050)

[4.5.2.1 Blinding method 24](#_Toc497833051)

[4.5.2.2 Confirmation of indistinguishability of investigational product 24](#_Toc497833052)

[4.5.2.3 Preparation and storage of the master assignment list and emergency key 24](#_Toc497833053)

[5 Investigational Product 25](#_Toc497833054)

[5.1 Test Drug and Control Drug 25](#_Toc497833055)

[5.1.1 Test drug 25](#_Toc497833056)

[5.1.2 Control drug-1 (test drug [KHK7580] placebo) 25](#_Toc497833057)

[5.1.3 Control drug-2: KRN1493 25](#_Toc497833058)

[5.2 Packaging and Labeling 25](#_Toc497833059)

[5.2.1 Packaging form 25](#_Toc497833060)

[5.2.2 Labeling 25](#_Toc497833061)

[5.3 Storage Method 25](#_Toc497833062)

[5.4 Delivery, Storage, Accountability, and Return of Investigational Product 26](#_Toc497833063)

[6 Enrollment Criteria 27](#_Toc497833064)

[6.1 Subjects (Disease) 27](#_Toc497833065)

[6.2 Inclusion Criteria 27](#_Toc497833066)

[6.3 Exclusion Criteria 27](#_Toc497833067)

[7 Dosing plan and combination therapy 29](#_Toc497833068)

[7.1 Dose, Dosing Method and Period 29](#_Toc497833069)

[7.2 Concomitant Medications and Therapies 29](#_Toc497833070)

[7.2.1 Prohibited Concomitant Medications and Therapies 29](#_Toc497833071)

[7.2.2 Restricted concomitant medications and therapies 30](#_Toc497833072)

[7.2.3 Concomitant medications to be used with care 30](#_Toc497833073)

[8 Study Enrollment 31](#_Toc497833074)

[8.1 Procedure for Subject Enrollment 31](#_Toc497833075)

[8.1.1 Assignment of subject ID code 31](#_Toc497833076)

[8.2 Enrollment Procedures 31](#_Toc497833077)

[8.3 Contact Information for Registration 33](#_Toc497833078)

[9 Observation, Examination, Test Items and Implementation Schedule 34](#_Toc497833079)

[9.1 Implementation Schedule 34](#_Toc497833080)

[9.2 Subject Demographic Information 35](#_Toc497833081)

[9.3 Observation and Examination Items and Timing of Implementation 35](#_Toc497833082)

[9.3.1 Exposure Investigation 35](#_Toc497833083)

[9.3.2 Dialysis Status Investigation 35](#_Toc497833084)

[9.4 Test Parameters and Timing of Implementation 36](#_Toc497833085)

[9.4.1 Laboratory Tests 36](#_Toc497833086)

[9.4.1.1 Hematology 36](#_Toc497833087)

[9.4.1.2 Blood biochemical tests-1 36](#_Toc497833088)

[9.4.1.3 Blood biochemical tests-2 36](#_Toc497833089)

[9.4.1.4 Bone metabolic markers 37](#_Toc497833090)

[9.4.2 Vital Signs and other tests 37](#_Toc497833091)

[9.4.2.1 Vital sign 37](#_Toc497833092)

[9.4.2.2 Height 37](#_Toc497833093)

[9.4.2.3 Body weight 37](#_Toc497833094)

[9.4.2.4 Electrocardiography 38](#_Toc497833095)

[9.4.2.5 Ophthalmologic examination 38](#_Toc497833096)

[9.4.2.6 Carotid ultrasonography 39](#_Toc497833097)

[9.4.2.7 Pregnancy Test 39](#_Toc497833098)

[9.5 Drug Level Measurements 39](#_Toc497833099)

[9.5.1 Plasma KHK7580 level 39](#_Toc497833100)

[9.5.1.1 Blood Sampling and Treatment of Samples 39](#_Toc497833101)

[9.5.1.2 Timing of blood sampling 40](#_Toc497833102)

[9.5.2 Measurement methods and testing protocol 40](#_Toc497833103)

[9.5.3 Plasma sample transport method 40](#_Toc497833104)

[9.5.4 Measurement result reporting 40](#_Toc497833105)

[9.6 Storage and Use of Blood Samples for Pharmacogenomic Tests 40](#_Toc497833106)

[9.6.1 Purpose of storage of blood samples for pharmacogenomic tests 40](#_Toc497833107)

[9.6.2 Subjects whose blood samples for pharmacogenomic tests are stored 40](#_Toc497833108)

[9.6.3 Methods of storage of blood samples for pharmacogenomic tests 41](#_Toc497833109)

[9.6.3.1 Timing of sampling 41](#_Toc497833110)

[9.6.3.2 Methods for sampling 41](#_Toc497833111)

[9.6.3.3 Storage of blood samples 41](#_Toc497833112)

[9.6.4 Procedures for withdrawal of informed consent 41](#_Toc497833113)

[9.6.5 Timing of starting pharmacogenomic test using a stored sample and the method 41](#_Toc497833114)

[9.6.6 Disclosure of the results of pharmacogenomic tests to subjects 41](#_Toc497833115)

[9.7 Adverse Events 42](#_Toc497833116)

[9.7.1 Definition of Adverse Events and Adverse Reactions 42](#_Toc497833117)

[9.7.1.1 Definition of serious adverse even 42](#_Toc497833118)

[9.7.1.2 Definition of other significant adverse events 42](#_Toc497833119)

[9.7.2 Assessments 42](#_Toc497833120)

[9.8 Number and Amount of Blood Draw 44](#_Toc497833121)

[10 Management of Subjects 45](#_Toc497833122)

[10.1 Notification to Other Hospitals and Departments 45](#_Toc497833123)

[10.2 Drug Administration Guidance and Survey 45](#_Toc497833124)

[10.3 Guidance for Subjects 45](#_Toc497833125)

[10.3.1 Hospital visits 45](#_Toc497833126)

[10.3.2 Food and drink 45](#_Toc497833127)

[10.3.3 Contraception 46](#_Toc497833128)

[11 Report of Adverse Events 47](#_Toc497833129)

[11.1 Adverse Events to be Reported 47](#_Toc497833130)

[11.1.1 Definition of serious adverse events 47](#_Toc497833131)

[11.1.2 Actions to take upon the occurrence of an adverse event and follow-up investigation 47](#_Toc497833132)

[11.1.2.1 Actions for subjects 47](#_Toc497833133)

[11.1.2.2 Report to related parties 47](#_Toc497833134)

[11.1.2.3 Follow-up of adverse events 48](#_Toc497833135)

[11.1.2.4 Handling of pregnancy 48](#_Toc497833136)

[12 Discontinuation Criteria and Procedures 49](#_Toc497833137)

[12.1 Discontinuation of a Relevant Subject 49](#_Toc497833138)

[12.1.1 Discontinuation criteria 49](#_Toc497833139)

[12.1.2 Discontinuation Procedures 49](#_Toc497833140)

[12.2 Discontinuation or Suspension at the Investigative Site 49](#_Toc497833141)

[12.3 Discontinuation or Suspension of the Entire Study 50](#_Toc497833142)

[13 Endpoints 51](#_Toc497833143)

[13.1 Efficacy 51](#_Toc497833144)

[13.1.1 Primary endpoint 51](#_Toc497833145)

[13.1.2 Secondary endpoints 51](#_Toc497833146)

[13.1.3 Exploratory endpoints 51](#_Toc497833147)

[13.2 Safety endpoints 51](#_Toc497833148)

[13.3 Pharmacokinetic endpoint 51](#_Toc497833149)

[14 Statistical Analysis 52](#_Toc497833150)

[14.1 Statistical Methods 52](#_Toc497833151)

[14.1.1 Efficacy analysis 52](#_Toc497833152)

[14.1.1.1 Primary endpoint 52](#_Toc497833153)

[14.1.1.2 Secondary Endpoints 52](#_Toc497833154)

[14.1.2 Analysis of Safety 53](#_Toc497833155)

[14.2 Target number of subjects 53](#_Toc497833156)

[14.3 Significance Level Used 54](#_Toc497833157)

[14.4 Study Discontinuation Criteria 54](#_Toc497833158)

[14.5 Handling of Missing, Unused, and Abnormal Data 54](#_Toc497833159)

[14.6 Development of Statistical Analysis Plan and Procedure for Reporting Deviations from the Original Analysis Plan 54](#_Toc497833160)

[14.7 Selection of Subjects Included in Analysis Sets 54](#_Toc497833161)

[14.7.1 Full analysis set (FAS) 54](#_Toc497833162)

[14.7.2 Per protocol set (PPS) 55](#_Toc497833163)

[14.7.3 Safety analysis set 55](#_Toc497833164)

[14.7.4 Pharmacokinetic analysis set 55](#_Toc497833165)

[15 Ethics 56](#_Toc497833166)

[15.1 IRB 56](#_Toc497833167)

[15.1.1 Review of feasibility of study implementation 56](#_Toc497833168)

[15.1.2 Continued review 56](#_Toc497833169)

[15.2 Selection of a Prospective Subject and Assurance of Safety 56](#_Toc497833170)

[15.3 Protection of Personal Information and Privacy of Subjects 56](#_Toc497833171)

[15.4 Timing and Method of Obtaining Informed Consent 56](#_Toc497833172)

[15.4.1 Information for subjects and informed consent 56](#_Toc497833173)

[15.4.1.1 Information for subjects and informed consent 56](#_Toc497833174)

[15.4.2 Storage of informed consent form 57](#_Toc497833175)

[15.5 Information for Subjects/Informed Consent Form 57](#_Toc497833176)

[15.6 Supply of Information to Subjects, Revision of Information for Subjects/Informed Consent Form and Reacquisition of Consent 59](#_Toc497833177)

[16 Monetary Payment and Compensation for Health Injury and Liability 60](#_Toc497833178)

[16.1 Study-related expenses 60](#_Toc497833179)

[16.2 Expenses for reducing burden of subjects associated with study participation 60](#_Toc497833180)

[16.3 Compensation for health injury and liability 60](#_Toc497833181)

[17 Compliance with the Protocol and Deviation/Modification and Revision 61](#_Toc497833182)

[17.1 Compliance with the Protocol 61](#_Toc497833183)

[17.2 Protocol Deviation or Modification 61](#_Toc497833184)

[17.3 Protocol Amendments 61](#_Toc497833185)

[18 Precautions for Creation of Electronic Case Report Form 62](#_Toc497833186)

[18.1 Creation of Electronic Case Report Form and Reporting 62](#_Toc497833187)

[18.2 Change or Correction of Electronic Case Report Form 62](#_Toc497833188)

[18.3 Items Inputted in the Electronic Case Report Form That May be Handled as the Source Material (source data) 62](#_Toc497833189)

[19 Direct access to source materials 64](#_Toc497833190)

[20 Quality Control and Quality Assurance of Clinical Study 64](#_Toc497833191)

[21 Study Period 64](#_Toc497833192)

[22 Study Completion 64](#_Toc497833193)

[23 Storage of Records 64](#_Toc497833194)

[23.1 Storage at IRB 64](#_Toc497833195)

[23.2 Storage at the investigative site 65](#_Toc497833196)

[23.3 Storage by the Investigator 65](#_Toc497833197)

[23.4 Storage by the Sponsor 65](#_Toc497833198)

[23.5 Storage of Source Materials Related to Measurement of Plasma KHK7580 Level 66](#_Toc497833199)

[23.6 Storage of Source Materials Related to Laboratory Test, etc. 66](#_Toc497833200)

[23.7 Storage of Biological Samples 66](#_Toc497833201)

[23.8 Storage of Blood Samples for Use in Pharmacogenomic Tests 67](#_Toc497833202)

[24 Publication of Study Results 67](#_Toc497833203)

[25 Rationale 68](#_Toc497833204)

[25.1 Rationale for Establishing Study Design 68](#_Toc497833205)

[25.2 Rationale for Establishing Inclusion Criteria 68](#_Toc497833206)

[25.3 Rationale for Establishing Exclusion Criteria 68](#_Toc497833207)

[25.4 Rationale for Establishing Dose, Method and Duration of Administration 69](#_Toc497833208)

[25.5 Rationale for Establishing Prohibited Concomitant Medications and Therapies 69](#_Toc497833209)

[25.6 Rationale for Establishing Restricted Concomitant Medications and Therapies 69](#_Toc497833210)

[25.7 Rationale for Establishing Measurement of Plasma KHK7580 Level 69](#_Toc497833211)

[25.8 Rationale for Establishing Instructions to Subjects 70](#_Toc497833212)

[25.9 Rationale for Establishing Test Items 70](#_Toc497833213)

[25.10 Rationale for Establishing Discontinuation Criteria 70](#_Toc497833214)

[25.11 Rationale for Establishing Efficacy Endpoints 70](#_Toc497833215)

[25.12 Rationale for Establishing Target Number of Subjects 71](#_Toc497833216)

[26 Study Implementation System 73](#_Toc497833217)

[27 Major Responsibilities of the Investigator 73](#_Toc497833218)

[28 References 74](#_Toc497833219)

# 1 History and Background of Study Plan

## 1.1 Background

In Japan, the number of patients under maintenance dialysis therapy is increasing every year with over 5,000 patients increase per year, and total number of patients exceeds 300,000 at the end of 2011^1)^. Furthermore, the number of dialysis patients per 1,000,000 population is about 2,400, indicating that about one in 400 Japanese people receives dialysis. This rate is also increasing yearly^1)^. In addition, the number of long-term dialysis patients with at least a 10-year history of dialysis has reached about 80,000^1)^.

In patients with severe chronic kidney disease (CKD) in whom dialysis is indicated, the original renal function that controls Ca and P metabolism declines markedly, and consequently various disorders of bone and mineral metabolism occur. Such bone and mineral metabolism disorders in CKD influence not only bone lesions, but also life prognoses through the calcification of soft tissues such blood vessels in the long-term. Recently, this has led to the proposal of the new concept “CKD-Mineral and Bone Disorder (CKD-MBD)” with respect to systemic disease^2)^.

Of the CKD-MBD, secondary hyperparathyroidism (SHPT) occurs at a high incidence. Decreased renal function may cause a decrease in urinary excretion of P and result in accumulation of P in the body (hyperphosphatemia). In addition, decreased production of active vitamin D_3_ in the kidney as well as a failure in Ca resorption in the kidney may cause decreased absorption of Ca in the small intestine and result in hypocalcemia. In a biological reaction for improving these conditions, parathyroid hormone (PTH), which inhibits the resorption of P from the urine and stimulates productions of active vitamin D_3_, is secreted from the parathyroid gland. Severe renal disorders, however, can cause the action of PTH to remain inadequate, and consequently hyperphosphatemia and hypocalcemia further persist, thereby continuing to stimulate the secretion of PTH. As a result of the stimulation continued for a long time, the parathyroid gland becomes hypertrophied, and consequently, even when the blood concentration of Ca is elevated, insufficient inhibition of PTH secretion occurs, thereby falling into a state of excessive secretion. This condition is called SHPT. In SHPT, excessive action of PTH may cause bone metabolic turnover to grow, leading to loss of bone strength (osteitis fibrosis) and deposition of P and Ca crystals in organs other than bone (ectopic calcification), followed by symptoms such as pain and pruritus. In addition, vascular calcification is responsible for arteriosclerosis and adversely affects the QOL and life prognosis of patients. This requires aggressive correction for pathological conditions and the main goal of improving and controlling the balance among P, Ca, and PTH is established in SHPT. The Japanese Society for Dialysis Therapy (JSDT) has published a clinical practice guideline for the management of CKD-MBD including SHPT. In this guideline, target ranges for serum P, Ca, and PTH levels are specified^3)^.

Active vitamin D preparations and phosphate binders had been traditionally used in drug therapy for SHPT condition. In 2008, however, cinacalcet hydrochloride (Regpara^®^), a Ca receptor agonist, became available also in Japan. Cinacalcet hydrochloride is a compound (calcimimetic agent) that acts on Ca receptors on the surface of parathyroid cells to enhance their sensitivity to Ca, thereby inhibiting the secretion of PTH. This compound has become widely available because it exerts a strong inhibitory effect on the secretion of PTH without elevating the serum Ca level. According to the annual report of the Dialysis Outcomes and Practice Patterns Study (DOPPS), which is researching therapeutic approach and prognosis in hemodialysis patients, 40.4% of hemodialysis patients in Japan received treatment with cinacalcet hydrochloride and/or intravenous vitamin D preparations in 2011^4)^. Such drug therapy patients are considered to have been treated for SHPT condition; therefore, the number of domestic SHPT patients on therapy is estimated to be about 120,000, assuming that the number of maintenance dialysis patients is about 300,000.

Cinacalcet hydrochloride has a high beneficial effect, whereas it produces adverse drug reactions on the upper gastrointestinal tract at a certain incidence. Of the 573 patients treated with cinacalcet hydrochloride in clinical studies in SHPT patients receiving maintenance hemodialysis in Japan, 124 (21.6%) had nausea/vomiting, 107 (18.7%) stomach discomfort, 56 (9.8%) anorexia, and 34 (5.9%) abdominal distension^5)^. These symptoms cast a burden on patients and, at the same time, contribute to a failure to increase the dosage of cinacalcet hydrochloride to a thoroughly effective level. In addition, cinacalcet hydrochloride has a strong inhibitory effect on the cytochrome P450 (CYP) 2D6 and, therefore, is likely to increase the blood concentration of drugs, such as tricyclic antidepressants or butyrophenone antipsychotics. Caution must therefore be exercised in using cinacalcet hydrochloride in combination with any such drug. In view of this situation, the next-generation calcimimetic agent that provides relief of the relevant problems of cinacalcet hydrochloride is desired.

## 1.2 Explanation of the Study Drug

KHK7580 is a new calcimimetic agent resulting from a joint research project conducted at Mitsubishi Tanabe Pharma Corporation and Kirin Pharma Co., Ltd. (now Kyowa Hakko Kirin Co., Ltd.). It has been suggested that this compound, like cinacalcet hydrochloride, acts on Ca receptors on the surface of parathyroid cells to inhibit the secretion of PTH. While cinacalcet hydrochloride induces gastrointestinal adverse reactions such as nausea and vomiting, KHK7580 is a compound that might reduce the onset of these events as shown in Section [1.3.1](#_1.3.1_Pharmacological_study). This indicates that KHK7580 is useful even for patients receiving cinacalcet hydrochloride in whom the occurrence of gastrointestinal adverse reactions has made it impossible to use cinacalcet hydrochloride or to increase the dosage to a necessary level. KHK7580 thus holds promise in further contributing to the attainment of the target PTH control level. Furthermore, KHK7580 has no strong inhibitory effect on major CYP isoforms; therefore, KHK7580, as compared with cinacalcet hydrochloride, is considered likely to become an easy-to-use drug also from the viewpoint of drug interactions.

## 1.3 Results of Non-Clinical Studies

### 1.3.1 Pharmacological study

After multiple oral administration of KHK7580 (0.03-, 0.1-, 0.3- and 1-mg/kg) once daily for 2 weeks to 5/6 nephrectomized rats, serum PTH and Ca levels were significantly lowered after the first and 14^th^ dose at 0.1 mg/kg and higher.

Investigation of the effect of a single oral dose of KHK7580 and cinacalcet hydrochloride to rats on gastric emptying showed that KHK7580 had no impact on gastric emptying even at 100-fold of the dose (0.03 mg/kg) that significantly lowered the PTH level in normal rats. On the other hand, cinacalcet hydrochloride significantly decreased gastric emptying at 30-fold of the dose (1 mg/kg) that significantly lowered the PTH level in normal rats. The above results suggest that KHK7580 would have a small impact on gastric emptying in rats compared with cinacalcet hydrochloride.

The impact of single oral dose of KHK7580 (1-, 3- and 10-mg/kg) on blood pressure and heart rate was investigated without anesthesia in male rats implanted with a telemetry transmitter. As a result, heart rate increased at doses 3 mg/kg and higher and blood pressure (diastolic and mean blood pressure) temporarily increased at 10 mg/kg. The impact of a single oral dose of KHK7580 (1-, 3- and 10-mg/kg) on blood pressure, heart rate, and ECG was investigated in male cynomolgus monkeys implanted with a telemetry transmitter. As a result, heart rate increased at doses 3 mg/kg and higher. Blood pressure increased, and QT interval corrected by heart rate (QTc) was prolonged at 10 mg/kg.

### 1.3.2 Pharmacokinetics and drug metabolism

After a single oral dose of KHK7580 (0.1-, 0.3- and 1.0-mg/kg) to rats, plasma KHK7580 reached C_max_ between 0.25 and 0.81 hours after administration and t_1/2_ disappeared between 5.77 and 6.66 hours after administration. C_max_ and AUC_0-∞_ increased dose dependently, and a linearity was observed in the dose range between 0.1 and 1.0 mg/kg. F ranged between 81.6% and 85.6%. After administration of KHK7580 (0.03-, 0.1-, 0.3- and 1-mg/kg) once daily for two weeks to 5/6 nephrectomized rats, plasma KHK7580 levels increased dose dependently and the plasma KHK7580 level 24-hr post dose was comparable after doses 7 and 14. The plasma KHK7580 level 24-hr post dose in this study was ≥10-fold of that of normal rats, and systemic clearance of KHK7580 was possibly lowered in partially nephrectomized rats.

In the investigation using human hepatic cells, naphthyl ethylamine, KHK7580 conjugates (glucuronic acid conjugate, taurine conjugate, glycine conjugate) and α-oxidant of phenylacetic acid base were detected as the metabolites of KHK7580. All metabolites were detected in the examination of hepatic cells of rats, dogs or monkeys, and no human-specific metabolite was observed.

The effect of KHK7580 on the substrate metabolism specific to each of the molecular species (CYP1A2, CYP2A6, CYP2B6, CYP2C8, CYP2C9, CYP2C19, CYP2D6, CYP2E1 and CYP3A4/5) was investigated in human hepatic microsomes. KHK7580 showed concentration-dependent inhibitory action to metabolic activity specific to CYP2D6 and time dependent inhibitory action to metabolic activity specific to CYP3A4/5, but 50% inhibitory activity to all CYP molecular species was higher than 50 μmol/L, the upper limit of the examined concentration.

After a single oral dose of [^14^C]KHK7580 (1 mg/kg) to male rats, administered radioactivity was immediately excreted within 48 hr after administration, and administered radioactivity was mainly excreted in feces.

### 1.3.3 Toxicity

#### 1.3.3.1 Multiple-dose toxicity studies

The results of 2-week multiple oral administration study of KHK7580 (0.3-, 1-, 3- and 10-mg/kg) once daily (2-week study in rats), 4-week multiple oral administration of KHK7580 (0.1-, 0.3-, 1.5- and 6-mg/kg) once daily (4-week study in rats), and 13-week multiple oral administration of KHK7580 (male: 0.3-, 1.5- and 6-mg/kg, female: 0.1-, 0.6- and 3-mg/kg) once daily (13-week study in rats) in male and female rats revealed one death in the toxicokinetic group at 10 mg/kg in 2-week study in rats, but no other death was observed either during the treatment or withdrawal periods. Commonly observed toxicological findings include muscle spasms (2-week study in rats: females in the ≥3 mg/kg groups, 4-week study in rats: males and females in the 6 mg/kg group, 13-week study in rats: males in the 6 mg/kg group) and loss of fur (2-week study in rats: female in the ≥3 mg/kg groups). Ophthalmological examination showed lens opacity (2-week study in rats: males and females in the ≥1 mg/kg groups, 4-week study in rats: all groups, 13-week study in rats: all groups other than females in the 0.1 mg/kg group).

In a 2-week multiple oral administration study of KHK7580 (1-, 3- and 10-mg/kg) once daily (2-week study in monkeys), 4-week multiple oral administration study of KHK7580 (0.1-, 0.3-, 1- and 3-mg/kg) once daily (4-week study in monkeys), and 13-week multiple oral administration study of KHK7580 (0.3-, 1- and 3-mg/kg) once daily (13-week study in monkeys) in male and female cynomolgus monkeys, no deaths occurred either during treatment or withdrawal periods, but the general condition of one male in the 10 mg/kg group in the 2-week study in monkeys was aggravated (muscle spasms, flexed wrists, weakness, and prone position) and administration was discontinued on Day 5 onward. Observed toxicological finding was QTc prolongation by Holter ECG (2-week study in monkeys: females in the ≥1 mg/kg groups and males in the 10 mg/kg group, 4-week study in monkeys: males and females in the 1- and 3-mg/kg groups, 13-week study in monkeys: males in the ≥1 mg/kg groups and females in the 3 mg/kg group). One female monkey’s heart rate was increased in the 3 mg/kg group in the 13-week study in monkeys. Ophthalmological examination did not show any changes attributable to the administration of KHK7580.

To investigate the relationship between the lens opacity of the eye and the lowered blood calcium level observed in multiple-dose toxicity studies in rats, the impact on lens opacity was investigated by constructing a test system for suppressing reduction of blood calcium level induced by the administration of KHK7580 via continuous intravenous administration of calcium gluconate solution. Increase in occurrence and worsening of lens opacity in the anterior cortex of lens developed dose dependently in the KHK7580 at 6- and 10-mg/kg monotherapy groups, but severity of lens opacity was suppressed and the number of occurrences was decreased by supplementation of calcium gluconate solution at 150 mg/kg/h. Pathohistological examination revealed swelling of the lens fiber in the 10 mg/kg monotherapy group but no lesion was observed after supplementation of calcium gluconate solution. The result suggested that lens opacity induced by multiple administration of KHK7580 in rats was induced by a lowered blood calcium level.

#### 1.3.3.2 Genotoxicity and reproductive/developmental toxicity studies

Genotoxicity of KHK7580 was assessed by bacterial reverse mutation test using bacteria and chromosomal aberration test using mammalian culture cells. The results showed that KHK7580 did not induce bacterial reverse mutation or *in vitro* chromosomal aberration. *In vivo* chromosomal aberration test was conducted by the administration of KHK7580 (10-, 30- and 100-mg/kg) once daily twice at an interval of 24 hours in male rats (bone marrow micronucleus test). Immature red blood cells with micronucleus did not increase in any of the groups compared with the medium group, so KHK7580 was judged not to induce chromosomal aberration in rat bone marrow cells.

Reproductive and developmental toxicity was investigated by examining the effect of once daily multiple oral administration of KHK7580 (0.3-, 1- and 3-mg/kg) to pregnant rats and KHK7580 (0.1-, 0.25- and 0.6-mg/kg) to pregnant rabbits during fetal organogenesis on dams and embryo/fetal development. None of the dams aborted, showed an abnormal general condition or died due to administration of KHK7580. Suppressed weight gain or decrease in body weight associated with a decrease in feed intake was observed in pregnant rats in the ≥1 mg/kg groups and pregnant rabbits in the 0.6 mg/kg group, but no abnormal finding was observed by autopsy. Examination of embryo/fetal development showed a low fetal weight in pregnant rats in the ≥1 mg/kg groups. An increase in the incidence of 14 rib bones (short and excessive rib bones) and bipartite sternebrae and lowered ossified metatarsal bones were observed in the 3 mg/kg group. No impact of KHK7580 was observed in pregnant rabbits in terms of appearance, visceral, skeletal, and placental development of fetus.

## 1.4 Results of Clinical Studies

### 1.4.1 Single-dose phase I study in healthy adult male subjects (Study 7580-001)

A single dose of KHK7580 or placebo was orally administered under fasting (6 subjects each in the KHK7580 0.3-, 1-, 3-, 6-, 12-, 20-mg groups and 12 subjects in the placebo group) or after a meal (6 subjects in the KHK7580 6-mg and 2 subjects in the placebo group) to healthy Japanese adult male subjects in a single-blind manner.

In terms of safety, adverse reactions of “abdominal discomfort,” “nausea” and “vomiting” developed in one subject each (17%) in the 20-mg group but all of them were mild in severity. With respect to laboratory data, vital signs and ECG, 12-lead ECG showed a tendency of QTcF prolongation in proportion to increase of KHK7580 dose, but a negative correlation was observed with corrected serum calcium concentration and it was considered associated with lowered blood calcium concentration. No clinically problematic variation was observed after administration of the study drug on the whole, and safety and tolerability were confirmed under the examined doses and dosage regimen. The pharmacokinetics after administration of KHK7580 under fasting is considered linear within the dose range between 1 and 20 mg, and no pronounced difference was observed in the amount of exposure of KHK7580 irrespective of administration of KHK7580 6 mg under fasting or after a meal. The result of pharmacodynamic analysis showed dose dependent decreases in intact PTH concentration and corrected serum calcium concentration in the cohort given administration under fasting, and low levels tended to continue with dose increase.

### 1.4.2 Multiple-dose phase I study in healthy adult male subjects (Study 7580-002)

KHK7580 or placebo (6 subjects each in the KHK7580 6- and 12-mg groups, and 6 subjects in the placebo group) was orally administered after a meal once daily for 8 days in healthy adult male subjects under single-blind condition to investigate the safety, tolerability, pharmacokinetics and pharmacodynamics.

Adverse events did not develop in the placebo group or the KHK7580 6-mg group but “tetany” (moderate) developed in one of 6 subjects in the 12-mg group, and it was judged an adverse drug reaction. This event disappeared and recovered after discontinuation of the study drug and administration of calcium gluconate. This event was considered attributable to hypocalcemia and no clinically problematic changes were observed in other laboratory data and vital signs after administration of the study drug. The result of 12-lead ECG showed a tendency of QTcF prolongation with increase in dose but no clinically significant safety problems were observed, and the safety and tolerability were confirmed with the investigated doses and dosage regimen. With respect to the pharmacokinetics of KHK7580, the plasma concentration reached a steady state immediately after the start of multiple oral administration of 6- and 12-mg once daily for 8 days. Similar changes in plasma concentration were observed on Day 1 and Day 8 both in the 6 and 12 mg groups and no accumulation was observed. The result of pharmacodynamic analysis showed decreases in intact PTH level and corrected the serum calcium level after administration of KHK7580.

### 1.4.3 Single-dose and multiple-dose phase I/II study in secondary hyperparathyroidism patients receiving chronic hemodialysis (Study 7580-003)

The safety, pharmacokinetics, and pharmacodynamics of KHK7580 were investigated in patients with secondary hyperparathyroidism (SHPT) receiving chronic hemodialysis after single-dose (1-, 4-, 12-mg), multiple-dose (1- and 4-mg for 14 days each), and extended (8- and 12-mg for 7 days each) administration.

With respect to safety, adverse reactions of “vomiting” developed in 1 of 28 patients (4%) after a single-dose of 4-mg, “nausea” in 1 of 26 patients (4%) after a single-dose of 12-mg, “blood calcium decreased” in 8 of 24 patients (33%) and “hypoesthesia” in 1 of 24 patients (4%) after multiple-dose of 4-mg. Three of the patients who developed “blood calcium decreased” discontinued the study and the event either disappeared or was recovered by the administration of calcium gluconate or observation of the course. “Blood calcium decreased” observed in 8 patients after multiple-dose of 4 mg was the only adverse reaction related to laboratory data, vital signs and ECG. Of the items analyzed by 12-lead ECG, slight prolongation of QTc interval was observed. A negative correlation with corrected serum calcium concentration was observed, and the event was considered associated with lowered blood calcium concentration. No clinically significant safety problem was observed on the whole and the safety and tolerability were confirmed at the investigated doses and dosage regimen. With respect to the pharmacokinetics, C_max_, AUC_0-t_ and AUC_0-∞_ mostly increased in proportion to the dose after a single-dose of KHK7580 between 1 and 12 mg. The pre-dose plasma KHK7580 concentration during multiple-dose and extended administration of KHK7580 changed mostly at a constant rate from Day 3 up to completion of administration. The result of pharmacodynamic analysis showed dose-dependent decreases in intact PTH level and corrected serum calcium level, and low levels tended to continue with increase of dose by single-dose administration and a low level continued up to completion of administration during multiple-dose and extended administration. The serum phosphorus level also showed a tendency to decrease by the administration of KHK7580.

## 1.5 Background of Preparation of This Protocol

As explained in Section [1.4.3](#_1.4.3_Single-dose_and), safety and tolerability were confirmed by the investigated doses and dosage regimen in Study 7580-003 where single doses (1-, 4-, 12-mg), multiple doses (1- and 4-mg respectively for 14 days), and extended doses (8- and 12-mg respectively for 7 days) of KHK7580 were administered to SHPT patients receiving hemodialysis. Dose dependent decrease in intact PTH concentration was observed and KHK7580 1-mg was presumed to be equivalent to KRN1493 25-mg in the rate of change in intact PTH concentration. Based on the above results, this study was planned to evaluate the efficacy and safety and to investigate the dose response profile by oral administration of KHK7580 (0.5-mg, 1-mg, 2-mg or placebo) or KRN1493 25-mg once daily for 3 weeks in SHPT patients receiving hemodialysis.

# 2 GCP Compliance Statement

This study is conducted based on the principles that have their basis in the Declaration of Helsinki and in compliance with the Pharmaceutical Affairs Law, Good Clinical Practice (GCP) (the Ministry of Health and Welfare Ordinance No. 28 dated March 27, 1997), and the partial revision of the Ordinance.

# 3 Objective of This Study

The objective of this study is to evaluate the efficacy and safety of 0.5, 1, or 2 mg KHK7580 or placebo, or 25 mg KRN1493 (cinacalcet hydrochloride) orally administered to subjects with secondary hyperparathyroidism (SHPT) receiving hemodialysis (HD) once daily for 3 weeks in a multicenter, randomized, placebo-controlled, double-blind, parallel-group, dose-response study (including the KRN1493 group as an open-control group).

# 4 Study Design

## 4.1 Study Design

Subjects who have consented to participation in the study will undergo screening prior to the start of dialysis on the dialysis date after the longest interval between dialysis sessions. Subjects who satisfy all the inclusion criteria will be enrolled prior to investigational product administration on Day 1 and then randomly and evenly assigned to the KHK7580 0.5, 1, or 2 mg, or placebo group, or the KRN1493 25 mg group using a dynamic allocation method. Administration of the investigational product will be started after assignment to each group on Day 1 and continued every day for 3 weeks. Assessments at the end of administration will be performed on Day 22, and there is then a follow-up period followed by final assessments on Day 29.


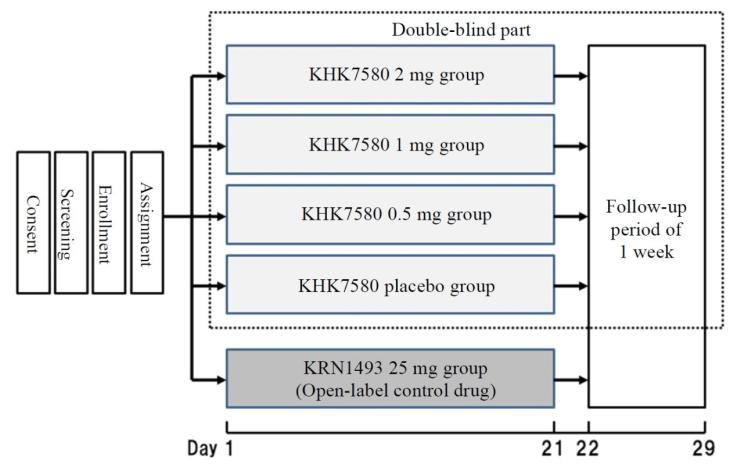


## 4.2 Type of This Study

A phase II clinical study (multicenter, randomized, placebo-controlled, parallel group, dose-response study [including KRN1493 group as an open-control group])

## 4.3 Endpoints

### 4.3.1 Efficacy evaluation

#### 4.3.1.1 Primary endpoint

• Percent change in intact PTH level from baseline at the end of study treatment

#### 4.3.1.2 Secondary endpoints

• Number and percentage of subjects achieving a percent decrease in intact PTH level of ≥30% (percent change ≤−30%) at the end of study treatment

• Number and percentage of subjects achieving an intact PTH level of ≤240 pg/mL at the end of study treatment

• Intact PTH level, whole PTH level, corrected serum Ca level, ionized Ca level, serum P level, intact fibroblast growth factor-23 (FGF23) level, and corrected serum Ca-P product at each time point and at the end of study treatment

Exploratory Endpoints:

• Bone metabolic markers at the end of study treatment and at the end of the follow-up period

• Parathyroid gland (volume and blood flow) at the end of study treatment

### 4.3.2 Safety evaluation

• Treatment-emergent adverse events (TEAEs)

• Lowest corrected serum Ca level between the start of study treatment and the end of the follow-up period

• Laboratory values

• Vital signs

• 12-lead electrocardiogram

• Ophthalmological examination

### 4.3.3 Pharmacokinetic evaluation

• Plasma KHK7580 level

## 4.4 Target Number of Subjects

150 subjects (30 subjects in each group) as subjects for investigational product administration

## 4.5 Randomization and Blinding

### 4.5.1 Randomization

#### 4.5.1.1 Randomization method

Subjects who satisfy all the inclusion criteria will be randomly and evenly assigned to the KHK7580 0.5, 1, or 2 mg, or placebo group, or the KRN1493 25 mg group using a dynamic allocation method. The assignment factors are investigative site, history of treatment with cinacalcet hydrochloride, corrected serum Ca level at screening (< 9.0 mg/dL, ≥ 9.0 mg/dL), and intact PTH level at screening (< 500 pg/mL, ≥ 500 pg/mL). Details of the subject assignment procedure are described in the separately specified procedure.

#### 4.5.1.2 Timing of randomization

After eligibility assessment of subjects, eligible subjects will be assigned to the KHK7580 0.5, 1, or 2 mg, or placebo group, or the KRN1493 25 mg group, and the subjects in KHK7580 0.5, 1, or 2 mg, or placebo group will be issued with drug numbers in the order of enrollment. Reuse of unused drugs from subjects who discontinued the study will be prohibited for any reason. In addition, discontinued subjects will not be permitted to re-enroll in the study.

### 4.5.2 Blinding

#### 4.5.2.1 Blinding method

Double-blinding (KHK7580 0.5 mg, 1 mg, 2 mg, or placebo group)

#### 4.5.2.2 Confirmation of indistinguishability of investigational product

Person Responsible for Investigational Product Assignment will check that the investigational products (KHK7580 0.5 mg, 1 mg, 2 mg, and placebo) are indistinguishable in appearance prior to allocation and upon unblinding of the master assignment list, and will report the results to the Sponsor.

#### 4.5.2.3 Preparation and storage of the master assignment list and emergency key

The Person Responsible for Investigational Product Assignment will prepare the drug master assignment list and seal it immediately after completion of assignment of investigational product. The assignment list will be stringently retained and managed until finalization of data and code breaking at the end of the study. In addition, the Person Responsible for Investigational Product Assignment will prepare and seal an emergency key to enable action in the case of an emergency such as occurrence of a serious adverse event.

The emergency key will be retained by the Person Responsible for Investigational Product Assignment, and will be opened in the event of an emergency in accordance with the separately defined procedure.

# 5 Investigational Product

## 5.1 Test Drug and Control Drug

### 5.1.1 Test drug

Company code number: KHK7580

Generic name: Not set

Chemical name: (4-{(3S) -3-[(1R) -1-(Naphthalen-1-yl) ethylamino]pyrrolidin-1-yl}phenyl) acetic acid

Molecular formula: C_24_H_26_N_2_O_2_

Molecular weight: 374.48

Content and dosage form:

KHK7580 0.5 mg tablets: A pale orange, film-coated tablet containing 0.5 mg of KHK7580

KHK7580 1 mg tablet: A orange yellow film-coated tablet containing 1 mg of KHK7580

KHK7580 2 mg tablet: A orange yellow film-coated tablet containing 2 mg of KHK7580

### 5.1.2 Control drug-1 (test drug [KHK7580] placebo)

A tablet that is indistinguishable from KHK7580 0.5 mg tablets, 1mg tablets, and 2 mg tablets, and contains no KHK7580

### 5.1.3 Control drug-2: KRN1493

A pale yellow to yellowish-green film-coated tablet containing 27.55 mg of cinacalcet hydrochloride (25 mg as cinacalcet)

## 5.2 Packaging and Labeling

### 5.2.1 Packaging form

Test drug: 10 tablets/PTP sheet/aluminum pillow packaging (containing oxygen absorber) /week, 3 sheets for 3 weeks are stored in an outer package.

Control drug-1: 10 tablets/PTP sheet/aluminum pillow packaging (containing oxygen absorber) /week, 3 sheets for 3 weeks are stored in an outer package.

Control drug-2: 10 tablets/PTP sheet, 3 sheets are stored in an outer package.

### 5.2.2 Labeling

The outer package is labeled with “for investigational use,” and the name and address of the Sponsor, company code number, contents, manufacturing number and storage method. The drug number is indicated for the test product and control drug-1.

## 5.3 Storage Method

To be stored at room temperature (1 to 30ºC).

## 5.4 Delivery, Storage, Accountability, and Return of Investigational Product

After executing a clinical study agreement, the Sponsor will dispense the investigational product to each investigative site. The Sponsor will develop a written procedure for controlling the investigational product and deliver it to each investigative site.

The investigational product manager at each investigative site will properly store and control the investigational product according to the procedure, and document the use of the investigational product, including inventory and collection. The investigational product manager will properly check unused and used supplies of the investigational product (including empty boxes) against the investigational product management record. The investigational product manager will seal the investigational product and return it to the Sponsor after the end of the study and upon reissue in association with a change of investigational product lot.

The investigational product manager will submit a copy of the investigational product management record to the Sponsor after the end of the study.

# 6 Enrollment Criteria

## 6.1 Subjects (Disease)

SHPT patients receiving hemodialysis

## 6.2 Inclusion Criteria

Subjects eligible for enrollment in the study had to meet all of the following criteria:

1) Personally submitted written voluntary informed consent to participate in the study

2) Aged ≥20 and <75 years old at the time of acquisition of consent

3) Stable chronic renal failure treated with hemodialysis 3 times weekly for at least 12 weeks before screening

4) Intact PTH level of ≥240 pg/mL at screening

5) Corrected serum Ca level of ≥8.4 mg/dL at screening

## 6.3 Exclusion Criteria

Subjects were excluded from participating in this study if they met any of the following criteria.

1) Use of cinacalcet hydrochloride within 2 weeks before screening

2) Change in dose or dosing regimen of an active vitamin D drug or its derivatives, phosphate binder, or calcium preparation within 2 weeks before screening; or start of treatment with such drugs within 2 weeks before screening

3) Change in prescribed conditions of dialysis (dialysate Ca concentration, dialyzer, prescribed dialysis time, and prescribed number of dialysis per week) within 2 weeks before screening

4) Treatment with bisphosphonates, denosumab, or teriparatide within 24 weeks before screening

5) Parathyroidectomy (PTx) and/or parathyroid intervention within 24 weeks before screening

6) Severe heart disease (e.g. ≥Class III based on New York Heart Association classification, protocol (supplement), Attachment 5)

7) Severe hepatic dysfunction (e.g. AST or ALT of ≥100 IU/L at screening)

8) Uncontrolled hypertension and/or diabetes mellitus

9) Pregnant, lactating, and possibly pregnant (positive pregnancy test or no pregnancy test performed and not using contraception), or unwilling to use an appropriate method of contraception as instructed by a physician; Amenorrhea for ≥12 months after the last menstrual period without an alternative medical cause is considered to be non-childbearing potential.

10) History of serious drug allergy; history or complications of drug or alcohol poisoning

11) History of drug allergy to cinacalcet hydrochloride

12) History of diagnosis and treatment of malignant tumor within 5 years before screening (excluding basal cell carcinoma or surgically resected intraepithelial carcinoma of uterine cervix)

13) Participation in a clinical study or equivalent study of a pharmaceutical product or medical device, and use of an investigational product or unapproved medical device within 12 weeks prior to screening

14) Prior treatment with KHK7580

15) Hyperparathyroidism primary

16) Other conditions unfit for participation in this study at discretion of the investigator or subinvestigator.

# 7 Dosing plan and combination therapy

## 7.1 Dose, Dosing Method and Period

KHK7580 (0.5 mg, 1 mg, 2 mg or placebo), or KRN1493 (25 mg) will be orally administered once daily for 3 weeks. Administration of the investigational product will be started from immediately before dialysis after the longest interval between dialysis sessions. From the day after the start of investigational product administration onwards, roughly the same timing will be used for administration as was used on the administration start date (administration immediately after the start of dialysis was also permitted).

## 7.2 Concomitant Medications and Therapies

When medications other than the investigational product are used after the date of starting investigational product administration up to study completion (discontinuation), the name of the medication, administration route, and treatment period will be inputted in the electronic case report form. However, active vitamin D preparations or derivatives (calcitriol, maxacalcitol, falecalcitriol, alfacalcidol, and eldecalcitol) P binders and Ca preparations (Ca preparations such as precipitated calcium carbonate, sevelamer hydrochloride, lanthanum carbonate, bixalomer, aluminum preparations, niceritrol, colestimide, cholestyramine, ferric citrate hydrate, etc.), and other food products with phosphate binding activity (calcium acetate, eggshell Ca, electricity-treated oyster shell powder, etc.) will be handled as the medications used after screening, and the name of the medication, dosage and administration, administration route, dosing intervals, treatment period, reason for use, and remarks concerning treatment noncompliance will be inputted in the electronic case report form. There is no need to input fluid replacement such as physiological saline or solution that is used for non-therapeutic purposes, local anesthesia for pain relief at the time of puncturing a needle, a contrasting agent for imaging diagnosis, and a mydriatic agent for mydriasis at the time of ophthalmological examination.

If any change is made to the dialysis conditions, the details of the change and the reason will be inputted in the electronic case report form.

### 7.2.1 Prohibited Concomitant Medications and Therapies

Concomitant use of the following medications and therapies is prohibited from the date of screening until the stipulated final assessments.

• Cinacalcet hydrochloride*

• Bisphosphonates

• Denosumab

• Teriparatide

• Calcitonin

• PTx and parathyroid intervention

• Peritoneal dialysis

* Except KRN1493 administered as control drug-2.

### 7.2.2 Restricted concomitant medications and therapies

1) In the event of any use of the following restricted concomitant medications and foods, changes in drug type and dosage and administration will be prohibited from the date of screening until the stipulated final assessments. In addition, patients not using restricted concomitant medications will not be permitted to start any new drugs during this period.

• Active vitamin D preparations or derivatives (calcitriol, maxacalcitol, falecalcitriol, alfacalcidol, and eldecalcitol)

• P binders and Ca preparations (Ca preparations such as precipitated calcium carbonate, sevelamer hydrochloride, lanthanum carbonate, bixalomer, aluminum preparations, niceritrol, colestimide, cholestyramine, ferric citrate hydrate, etc.), and other food products with phosphate binding activity (calcium acetate, eggshell Ca, electricity-treated oyster shell powder, etc.)

2) Changes in dialysis conditions (dialysate Ca level, dialyzer, prescribed dialysis time, and prescribed number of dialysis sessions per week) will be prohibited from the date of screening until the stipulated final assessments.

### 7.2.3 Concomitant medications to be used with care

When concomitantly using the following medications and food in a subject allocated to the KRN1493 25 mg group, provide an interval to the extent possible and carefully observe the subject (see Attachment 6 in the protocol [separate volume]).

• Azole antifungal drugs: itraconazole, etc.

• Macrolide antibiotics: erythromycin, clarithromycin, etc.

• Amiodarone hydrochloride

• Grapefruit (juice)

• Tricyclic antidepressants: amitriptyline hydrochloride, imipramine hydrochloride, etc.

• Butyrophenone antipsychotics: haloperidol, etc.

• Flecainide acetate

• Vinblastine sulfate

• Adrenocortical hormones: cortisone, prednisolone, dexamethasone, etc.

• Digitoxin

• Diazepam

# 8 Study Enrollment

## 8.1 Procedure for Subject Enrollment

The investigator or subinvestigator will explain the study to subjects who meet the inclusion criteria and do not meet the exclusion criteria, and obtain written consent for participation in the study. The investigator or subinvestigator will screen the subjects after obtaining their written consent and notify the 7580-005 Enrollment Center by facsimile using a “KHK7580 Phase 2 Clinical Study: Case Report Form” (see Attachment 3 in the protocol [separate volume]). Enrollment will be conducted from the date on which the confirmation results of all inclusion and exclusion criteria are determined (after screening result assessment) until before treatment with the investigational product on Day 1.

### 8.1.1 Assignment of subject ID code

The investigator or subinvestigator will assign a subject ID code to a subject from whom the written consent for study participation has been obtained. The subject ID code will be assigned based on the following rules.

Subject ID code: 7580-005-XX-YY

XX: Investigative site number (see Attachment 1 in the protocol [separate volume])

YY: Serial number of subjects from whom the consent for study participation has been obtained at the concerned investigative site.

Example) When informed consent is obtained for the first time at ⚫⚫ Hospital (Investigative site number: 01), subject ID code will be 7580-005-01-01.

The investigator or subinvestigator will record the subject ID code in the screening roster when assigning a subject ID code.

## 8.2 Enrollment Procedures

Study subjects will be enrolled according to the following procedures.

1) The investigator or subinvestigator will conduct the screening according to “[9](#_9_Observation,_Examination,) Observation, Examination, Test Items and Implementation Schedule” after obtaining informed consent. Retest may be repeated twice for the inclusion criteria 4), 5) and exclusion criterion 7).

2) The investigator or subinvestigator will confirm all the inclusion and exclusion criteria, and prepare “Case Registration Form for Phase II Study of KHK7580” (see Attachment 3 in the protocol [separate volume]) for all the subjects from whom informed consent was obtained irrespective of the applicability of the criteria, and send it via facsimile to 7580-005 Registration Center. Enrollment will be carried out after the date of determining the result of all inclusion and exclusion criteria (after finding out the screening results) and before administration of the investigational product on Day 1. However, when a subject withdraws consent or is found to be ineligible after obtaining the informed consent, “Case Registration Form for Phase II Study of KHK7580” may be prepared at that time and sent to 7580-005 Registration Center via facsimile.

3) 7580-005 Registration Center will check the eligibility of subjects, and send “Registration Confirmation Form for Phase II Clinical Study of KHK7580” to the investigator or subinvestigator via facsimile when eligibility is confirmed. In the case the contents of registration are unclear, 7580-005 Registration Center (or the sponsor who was contacted) may make inquiries to the investigator, subinvestigator, or the Sponsor. A subject who does not meet the inclusion criteria and/or meet the exclusion criteria will be specified as ineligible in the “Ineligibility Conformation Form for Phase II Clinical Study of KHK7580,” which will be sent to the investigator or subinvestigator via facsimile. The investigator or subinvestigator will input the reason for ineligibility to the electronic case report form.

4) The investigator or subinvestigator will record the enrolled subject in a screening roster and store it.

The enrolment procedure is summarized below.

Investigator or subinvestigator

7580-005 Registration Center

➀ Selection of subjects

Checking subject demographics.

➁ Obtaining informed consent and assigning subject ID code

Explain the clinical study, obtain written. informed consent, and assign subject ID code.

➂ Screening

Confirm eligibility by conducting screening.

➃ Registration

“Case Registration Form” will be sent to 7580-005 Registration Center via facsimile before administration of the investigational product on Day 1.

➅ Checking registration

Prescribe the instructed IP by checking the “Registration Confirmation Form.”

➄ Checking eligibility and feasibility of registration, and allocation of the subjects

When a subject is judged eligible after checking eligibility, IP will be allocated, and “Registration Confirmation Form” containing the drug number and necessary items will be sent to the investigator or subinvestigator via facsimile.

When a subject is judged ineligible, “Ineligibility Confirmation Form” will be sent to the investigator or subinvestigator via facsimile.

## 8.3 Contact Information for Registration

| Contact information for registration  Name: 7580-005 Registration Center  Facsimile: 0120-727-770  Phone: 0120-017-081  Office hours: Monday to Saturday from 9:00 to 18:00 (except for Sundays, holidays, and year end to new year between 12/30 and 1/3). |
| --- |

# 9 Observation, Examination, Test Items and Implementation Schedule

## 9.1 Implementation Schedule

All examinations stipulated during the investigational product administration period will be performed prior to investigational product administration and prior to the start of dialysis, except for 1 hour and 3 hours after administration on the day of starting administration (Day 1). Except for screening and the ophthalmologic examination and carotid ultrasonography stipulated on Day 22 and Day 29, all tests will be performed prior to the start of dialysis, but this restriction will not apply to assessment upon discontinuation.

The following range will be permitted for hospital visits.

• Day 3, 5, 8, 10, 12, 15, 17, 19, 22, 29: ± 1 day

Assessments upon discontinuation will be performed as soon as possible after the subject discontinued the study.

Table 9.1-1 Observation, examination, and test schedule


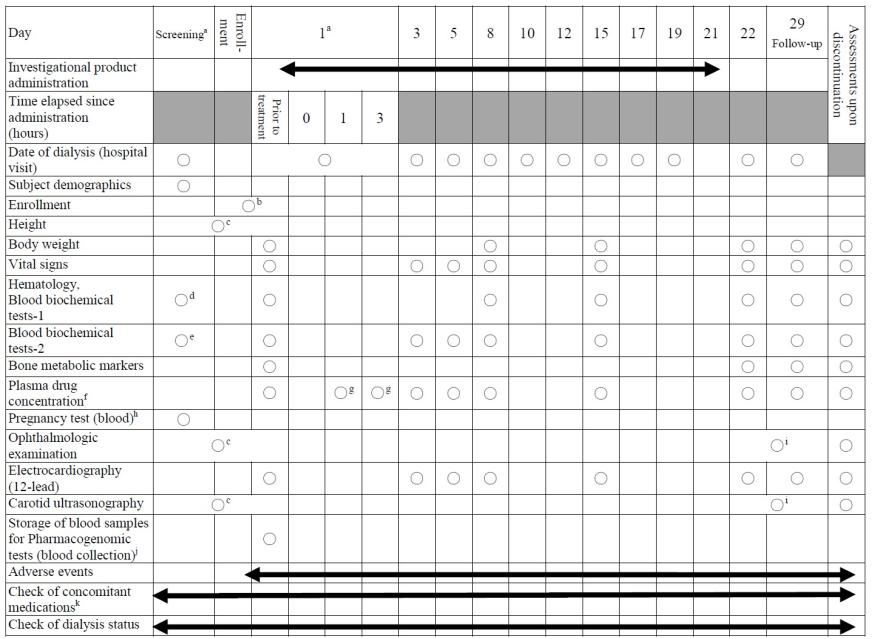


a: The date of dialysis after the longest interval between dialysis sessions will be used.

b: Performed from the day of completion of eligibility confirmation for all of the inclusion and exclusion criteria until pre-dose of investigational product on Day 1.

c: Performed from the time of acquisition of consent until pre-dose of investigational product on Day 1.

d: Only AST and ALT will be used. If the test value meets exclusion criterion 7), up to two retests will be permitted.

e: Only intact PTH, corrected serum Ca, Ca, and Alb will be used. If the values do not correspond to inclusion criteria 4) and 5), up to two retests will be permitted.

f: Blood will be only collected from subjects assigned to the KHK7580 0.5, 1, 2 mg, or placebo group.

g: Food intake will be prohibited from investigational product administration until the completion of blood sampling 3 hours after the administration.

h: Females only. However, no test will be required if at least 12 months have passed since the subject’s last menstruation in the absence of any other medical cause.

i: Performed within 14 days after Day 22.

j: Blood will be only collected from subjects assigned to the KHK7580 0.5, 1, 2 mg, or placebo group and who have consented to the storage and use of blood samples.

k: Prohibited concomitant medications and restricted concomitant medications will be checked from the date of screening, and other concomitant medications will be checked from Day 1.

## 9.2 Subject Demographic Information

The following items will be surveyed:

Acquisition date of written consent, date of birth, sex, concurrent diseases, medical history, primary disease, year and month of dialysis initiation, history of PTx and year and month of PTx, history of parathyroid intervention and year and month of parathyroid intervention, dialysis conditions (dialysate Ca level, dialysis status in routine clinical practice, dialyzer, dry weight, prescribed blood flow, prescribed dialysate flow rate, dialysis efficiency*), status of use of cinacalcet hydrochloride or other calcimimetics, current smoking habit, and other items related to the inclusion and exclusion criteria

Timing of implementation: At screening.

* Data obtained on the day of screening or the latest data before screening will be used (data prior to acquisition of consent can also be used).

## 9.3 Observation and Examination Items and Timing of Implementation

### 9.3.1 Exposure Investigation

The administration date and administration status of the investigational product will be investigated.

Timing of implementation:

From the date of starting investigational product administration (Day 1) to the date of completion of investigational product administration (Day 21).

### 9.3.2 Dialysis Status Investigation

The date and start time of dialysis will be investigated. The details and reason for any changes in dialysis conditions will also to be investigated.

Timing of implementation:

From the date of starting investigational product administration (Day 1) to the date of completion of follow-up observation (Day 29).

## 9.4 Test Parameters and Timing of Implementation

### 9.4.1 Laboratory Tests

Laboratory tests will be performed according to the specified test schedule to check for any variation in laboratory values since investigational product administration. All laboratory values will be determined by a central laboratory, and measurements will be reported to the Sponsor.

#### 9.4.1.1 Hematology

Parameters:

WBC, differential white blood cell count, RBC, Hb, Ht, PLT

Timing of implementation:

• Day 1 (prior to investigational product administration)

• Day 8, 15^*^ (prior to investigational product administration and prior to start of dialysis)

• Day 22, 29^*^ (prior to start of dialysis)

• Discontinuation

* When the central laboratory is closed, the tests of Day 8 and 15 may be conducted on Day 5 and 12, respectively. The tests of Day 29 may be conducted within 7 days.

#### 9.4.1.2 Blood biochemical tests-1

Parameters:

T-Cho, TG, TP, BUN, UA, Cr, T-Bil, AST, ALT, γ-GTP, LDH, ALP, CK, ChE, Na, K, Cl, Mg, calcitonin

Timing of implementation:

• At screening^*1^ (prior to start of dialysis)

• Day 1 (prior to investigational product administration)

• Day 8, 15^*2^ (prior to investigational product administration and prior to start of dialysis)

• Day 22, 29^*2^ (prior to start of dialysis)

• Discontinuation

*1: Only for AST and ALT.

*2: When the central laboratory is closed, the tests of Day 8 and 15 may be conducted on Day 5 and 12, respectively. The tests of Day 29 may be conducted within 7 days.

#### 9.4.1.3 Blood biochemical tests-2

Parameters:

Corrected serum Ca^*1^, Ca, Alb, P, intact PTH, whole PTH, ionized calcium, intact FGF23

Timing of implementation:

• At screening^*1^ (prior to start of dialysis)

• Day 1 (prior to investigational product administration)

• Day 3, 5, 8, 15 (prior to investigational product administration and prior to start of dialysis)

• Day 22, 29 (prior to start of dialysis)

• Discontinuation

*1: If the serum Alb level is <4.0 g/dL, the serum Ca level will be corrected in accordance with the following formula. If the serum Alb level is ≥4.0 g/dL, the measured serum Ca level will be treated as the corrected serum Ca level.
Corrected serum Ca level (mg/dL) = Measured serum Ca level (mg/dL) +4-serum Alb value (g/dL)

*2: only for intact PTH, serum corrected calcium, Ca and Alb levels.

#### 9.4.1.4 Bone metabolic markers

Parameters:

BAP, TRACP-5b, total P1NP, osteoprotegerin

Timing of implementation:

• Day 1 (prior to investigational product administration)

• Day 22, 29^*^ (prior to start of dialysis)

• Discontinuation

* When the central laboratory is closed, the tests of Day 29 may be conducted within 7 days.

### 9.4.2 Vital Signs and other tests

#### 9.4.2.1 Vital sign

Parameters:

Blood pressure (seated), pulse rate (seated), body temperature (axillary)

Timing of implementation:

• Day 1 (prior to investigational product administration)

• Day 3, 5, 8, 15 (prior to investigational product administration and prior to start of dialysis)

• Day 22, 29 (prior to start of dialysis)

• Discontinuation

#### 9.4.2.2 Height

Parameter:

Height

Timing of implementation:

To be measured between the date of obtaining informed consent and investigational product administration on Day 1.

#### 9.4.2.3 Body weight

Parameter:

Body weight

Timing of implementation:

• Day 1 (prior to investigational product administration)

• Day 8, 15 (prior to investigational product administration and prior to start of dialysis)

• Day 22, 29 (prior to start of dialysis)

• Discontinuation

#### 9.4.2.4 Electrocardiography

##### 9.4.2.4.1 12-Lead Electrocardiography

Subjects will be examined for electrocardiographic findings. Findings will be assessed for clinical significance.

Parameter:

Resting 12-lead electrocardiography

Timing of implementation:

• Day 1 (prior to investigational product administration)

• Day 3, 5, 8, 15 (prior to investigational product administration and prior to start of dialysis)

• Day 22, 29 (prior to start of dialysis)

• Discontinuation

##### 9.4.2.4.2 Analysis of Electrocardiographic Data

The electrocardiographic data obtained by the method described above will be dispatched to BioClinica, Inc. using an electronic transmission system. The following electrocardiographic measurements will be calculated at BioClinica, Inc. using the electrocardiographic data dispatched. BioClinica, Inc. will report the analysis results to the Sponsor.

Timings of measurement of ECG:

• Day 1 (prior to investigational product administration)

• Day 3, 5, 8, 15 (prior to investigational product administration and prior to start of dialysis)

• Day 22, 29 (prior to start of dialysis)

• Discontinuation

Electrocardiographic measurements:
Heart rate, RR interval, PR interval, QRS interval, QT interval, QTc (QTcF, QTcB) interval

#### 9.4.2.5 Ophthalmologic examination

Subjects were examined for any findings. Findings will be assessed for clinical significance.

Parameter:

Ophthalmologic examination using a slit lamp

Timing of implementation:

• Between the day of obtaining informed consent and investigational product administration on Day 1.

• Day 22^*^

• Discontinuation

*: It is allowed to conduct the tests of Day 22 within 14 days.

#### 9.4.2.6 Carotid ultrasonography

Parameters:

Longest diameter of parathyroid gland (3 dimensions) and blood flow*^1^

Timing of implementation:

• Between the day of obtaining informed consent and investigational product administration on Day 1.

• Day 22^*2^

• Discontinuation

*1: This will be performed only where available. The test method and calculation method are described in a separate operating procedure.

*2: The tests of Day 22 may be conducted within 14 days.

#### 9.4.2.7 Pregnancy Test

Parameter:

Pregnancy test (blood)

Timing of implementation:

• At screening.

* Females only. However, no test will be required if at least 12 months have passed since the subject’s last menstruation in the absence of any other medical cause.
Pregnancy tests will be performed by a central laboratory.

## 9.5 Drug Level Measurements

### 9.5.1 Plasma KHK7580 level

Plasma KHK7580 level will be only measured in the KHK7580 0.5 mg, 1mg, and 2 mg groups. However, blood will be also collected in the placebo group. Use of samples remaining after plasma drug level measurements will be permitted for analysis of KHK7580 metabolites outside of the study plan. In addition, use of drug level data obtained during the study in combination with data obtained from other studies will be permitted for population pharmacokinetic analysis.

#### 9.5.1.1 Blood Sampling and Treatment of Samples

Blood Sampling and Treatment of Samples:

Two mL of venous blood will be collected in a vacuum blood sampling tube containing EDTA-2K and promptly mixed by inversion. The plasma obtained by centrifugation will be divided into two equal fractions and dispensed into two sample tubes. The plasma samples will be frozen immediately after being dispensed and stored frozen at −20°C or below until the start of measurement. The time of blood sampling, whether or not the patient had a meal within 2 hours prior to investigational product administration on Day 1, and the date and time of administration of the investigational product immediately prior to blood sampling will be entered in the electronic case report form.

#### 9.5.1.2 Timing of blood sampling

Blood sampling time point (acceptable range):

• Day 1 (prior to investigational product administration) [From rising until immediately prior to administration]

• Day 1 (1 hour after administration, 3 hours after administration) [± 15 min in each case]

• Day 3, 5, 8, 15 (prior to investigational product administration and prior to start of dialysis)

• Day 22, 29 (prior to start of dialysis)

• Discontinuation

### 9.5.2 Measurement methods and testing protocol

Plasma KHK7580 levels will be measured by LC/MS/MS by the Pharmaceutical Business Division, Sumika Chemical Analysis Service, Ltd. Prior to measurement, the testing manager in the Pharmaceutical Business Division, Pharmaceutical Analysis Osaka Laboratory, Sumika Chemical Analysis Service, Ltd. will prepare a testing protocol for the level measurements. Plasma KHK7580 level will be only measured in the KHK7580 0.5 mg, 1mg, and 2 mg groups.

### 9.5.3 Plasma sample transport method

The Drug Development Service Segment, LSI Medience Corporation will collect the samples for drug level measurements that have been sampled at the investigative sites. LSI Medience Corporation will send the frozen samples to the Pharmaceutical Business Division, Pharmaceutical Analysis Osaka Laboratory, Sumika Chemical Analysis Service, Ltd. Note that the investigative sites, LSI Medience Corporation, Sumika Chemical Analysis Service, Ltd., and the Sponsor each will retain a sample transfer record and related information.

### 9.5.4 Measurement result reporting

Sumika Chemical Analysis Service will prepare a final report on the measurement results and submit it to the Sponsor.

## 9.6 Storage and Use of Blood Samples for Pharmacogenomic Tests

### 9.6.1 Purpose of storage of blood samples for pharmacogenomic tests

To exploratively investigate individual differences in response to KHK7580 (efficacy, adverse reactions, pharmacokinetics, pharmacodynamics, etc.) and its relationship with variation of DNA or RNA property (e.g., polymorphism).

### 9.6.2 Subjects whose blood samples for pharmacogenomic tests are stored

Of the subjects who consented to participate in this clinical study as well as to the storage and use of blood samples for pharmacogenomic tests in Section [9.6.1](#_9.6.1_Purpose_of), those who are allocated to KHK7580 0.5 mg, 1 mg, 2 mg or placebo group.

The date of obtaining informed consent will be inputted into the electronic case report form.

### 9.6.3 Methods of storage of blood samples for pharmacogenomic tests

#### 9.6.3.1 Timing of sampling

Blood sampling time point: Day 1 (prior to investigational product administration)

#### 9.6.3.2 Methods for sampling

A blood collection tube containing EDTA-2Na will be used for collecting 2 mL of blood via venous puncture, mixed by inverting, transferred to a sample tube, and stored frozen at −20°C or below.

#### 9.6.3.3 Storage of blood samples

Blood samples will be stored by double anonymization using a linkable anonymization number that is different from the subject ID code used in this study.

### 9.6.4 Procedures for withdrawal of informed consent

When a subject withdraws informed consent for storage of blood samples, the investigative site will promptly inform Kyowa Hakko Kirin Co., Ltd. Then, Kyowa Hakko Kirin Co., Ltd. will instruct LSI Medience Corporation to dispose of the blood samples of the concerned subject. LSI Medience Corporation will dispose of the blood samples and submit a report of disposal to Kyowa Hakko Kirin Co., Ltd. Kyowa Hakko Kirin Co., Ltd. will separately appoint a banking manager according to the procedures specified by Kyowa Hakko Kirin Co., Ltd. (“Procedures for a clinical study using pharmacogenomics”).

### 9.6.5 Timing of starting pharmacogenomic test using a stored sample and the method

Pharmacogenomic tests using the stored samples will be conducted only when the Sponsor acknowledges the usefulness of exploration of variations of DNA or RNA property (such as polymorphism) related to response to KHK7580. The genes to be tested will be those possibly related to the response to KHK7580, but they cannot be identified at present.

When specific details of this pharmacogenomic tests are determined, the plan for genomic/genetic analysis will be separately prepared according to the procedures specified by Kyowa Hakko Kirin Co., Ltd. (“Procedures for a clinical study using pharmacogenomics”), and reviewed and approved by the Ethics Committee established by Kyowa Hakko Kirin Co., Ltd. before implementation.

### 9.6.6 Disclosure of the results of pharmacogenomic tests to subjects

Even when a certain outcome is obtained by genomic/genetic researches using the stored samples, the result is exploratory or of an initial stage and its scientific reliability such as its precision and certainty has not been fully confirmed. Given that a subject might be disadvantaged by disclosure of information for which scientific assessment has not been established, obtained information (results of pharmacogenomic tests, etc.) will not be disclosed to subjects.

## 9.7 Adverse Events

### 9.7.1 Definition of Adverse Events and Adverse Reactions

An adverse event is any undesirable medical occurrence in a subject treated with an investigational product. More specifically, it is any undesirable or unintended sign (including any abnormal variation in laboratory values), symptom, or disease that occurs after administration of an investigational product, regardless of any relationship to the investigational product. An adverse reaction is an adverse event for which the causal relationship to the investigational product is assessed as “related” or “unknown.”

Results of laboratory parameters will be checked against the normal range for any deviations (abnormal values), and any abnormal test results will be compared with those obtained before the start of investigational product administration to determine whether such variation is clinically significant (abnormal variation). However, variations in intact PTH level, whole PTH level, corrected serum Ca level, ionized Ca level, and corrected serum Ca-P product will only be treated as abnormal variation if deemed medically problematic by the investigator or subinvestigator in view of the pharmacological effects of the investigational product. Variations in bone metabolic markers and intact FGF23 level will be excluded from the assessment of adverse events.

The diagnosis of any abnormal changes in signs, symptoms, and laboratory values associated with a disease (diagnosis) will be treated as an adverse event. However, any atypical or extremely severe signs or symptoms of the disease will be treated as adverse events according to the individual sign or symptom in the same way as for the diagnosis.

#### 9.7.1.1 Definition of serious adverse even

“Serious adverse events” involve the following circumstances. Hospitalization for testing and pre-planned hospitalization will be excluded.

1) Death

2) Events that may lead to death

3) Events requiring admission to a hospital or clinic, or prolonged hospitalization for treatment

4) Disability

5) Events that may lead to disability

6) Events that are as serious as the events in 1) to 5)

7) Congenital diseases or abnormalities in offspring

#### 9.7.1.2 Definition of other significant adverse events

Other significant adverse events are all non-serious adverse events that resulted in discontinuation of the study.

### 9.7.2 Assessments

The following items will be entered into the electronic case report form.

Adverse events will be followed up until recovery to condition prior to investigational product administration, or until the investigator or subinvestigator determines that no further follow-up is necessary based on the subject’s condition in terms of such factors as symptoms, findings, and laboratory values. As a guide, adverse events pertaining to “blood calcium decreased” will be followed up until the corrected serum Ca level recovers to ≥8.4 mg/dL. Treatment of the adverse event during follow-up will be entered in the electronic case report form.

1) Adverse event

2) Date of onset

3) Severity

• Mild (signs or symptoms are observed but do not interfere with daily activities)

• Moderate (discomfort is observed to interfere with daily activities or affect the subject’s clinical condition)

• Severe (daily activities are not possible or a major effect is observed on the subject’s clinical condition)

4) Serious event

• Serious: Events defined in [9.7.1.1](#_9.7.1.1_Definition_of)

• Non-serious: Not corresponding to events defined in [9.7.1.1](#_9.7.1.1_Definition_of)

5) Action taken with investigational product

• Not changed/not applicable

• Discontinuation

6) Other actions taken: Any action taken other than that with investigational product

• None

• Yes (If “Yes,” include details)

7) Outcome

• Recovered/Resolved

• Recovering/Resolving

• Not recovered

• Recovered/Resolved with sequelae

• Death

• Unknown

8) Date of outcome

9) Causal relationship to investigational product
Causal relationships to study treatment will be classified into the 3 categories below. In the case of “Not related,” the assessment rationale will be entered in the electronic case report form.

• Not related

• Unknown

• Related

## 9.8 Number and Amount of Blood Draw

Total number of blood draw: 10 times

Total amount of blood draw: 157 mL*

* When screening is repeated twice or more, the amount of blood drawn at the second test onward will not be included.

Details are shown in [Table 9.8-1](#_Hlk495681498). In consideration of the safety of subjects, number and amount of blood drawn may be increased when additional/follow-up tests are conducted.

Table 9.8-1 Details of amount of blood draw

| Day | Screening | 1 |  |  | 3 | 5 | 8 | 10 | 12 | 15 | 17 | 19 | 21 | 22 | 29 |
| --- | --- | --- | --- | --- | --- | --- | --- | --- | --- | --- | --- | --- | --- | --- | --- |
| Time elapsed since administration (hours) |  | Prior to treatment | 1 | 3 |  |  |  |  |  |  |  |  |  |  |  |
| Hematology |  | 2 |  |  |  |  | 2 |  |  | 2 |  |  |  | 2 | 2 |
| Blood biochemical tests-1^a^ Blood biochemical tests-2^b^ Pregnancy test (blood) ^c^ | 5 | 14 |  |  | 11 | 11 | 17 |  |  | 17 |  |  |  | 17 | 17 |
| Bone metabolic markers |  | 6 |  |  |  |  |  |  |  |  |  |  |  | 6 | 6 |
| Serum KHK7580 level^d^ |  | 2 | 2 | 2 | 2 | 2 | 2 |  |  | 2 |  |  |  | 2 | 2 |
| Storage of blood samples for Pharmacogenomic tests |  | 2 |  |  |  |  |  |  |  |  |  |  |  |  |  |
| Amount of blood drawn (mL) | 5 | 26 | 2 | 2 | 13 | 13 | 21 |  |  | 21 |  |  |  | 27 | 27 |

a: Only AST and ALT levels by screening.

b: Only intact PTH, corrected serum Ca, Ca and Alb levels by screening.

c: Females only. However, no test will be required if at least 12 months have passed since the subject’s last menstruation in the absence of any other medical cause.

d: Blood will be only collected from subjects assigned to the KHK7580 0.5, 1, 2 mg, or placebo group.

e: Blood will be only collected from subjects assigned to the KHK7580 0.5, 1, 2 mg, or placebo group and who have consented to the storage and use of blood samples.

# 10 Management of Subjects

## 10.1 Notification to Other Hospitals and Departments

The investigator or subinvestigator will check with subjects about whether they are scheduled to receive medical care or medications at any other department or hospital before the start of investigational product administration. If a subject is receiving medical care or medications at another department or hospital, the investigator will notify the treating physician that the subject will participate in the clinical study. The investigator will identify the name of any medication received by the subject other than those prescribed by the investigator as well as how it was used. If a subject has received medical care or medications at any other department/hospital during the study, the investigator will take similar steps.

## 10.2 Drug Administration Guidance and Survey

The investigator or subinvestigator will prescribe the investigational product to the subjects. Subjects will receive explanation and guidance on the dosage and administration and mode of use of the investigational product upon prescription. The subjects will be instructed to bring unused and used investigational product (including empty PTP sheets) with them on their next hospital visit. If a subject is found to be non-compliant to study treatment, they will be given thorough instructions once again. In case of overdose, in particular, the investigator or subinvestigator will take proper action based on safety considerations for the subject and determine whether the subject should continue to participate in the study.

## 10.3 Guidance for Subjects

The investigator, subinvestigator or study collaborators will provide the following guidance to subjects.

### 10.3.1 Hospital visits

Subjects will be screened after providing written consent.

The following range will be permitted for hospital visits.

• Day 3, 5, 8, 10, 12, 15, 17, 19, 22, 29: ± 1 day

Assessments upon discontinuation will be performed as soon as possible after the subject discontinued the study.

### 10.3.2 Food and drink

The investigator or subinvestigator will instruct subjects to fast from investigational product administration on Day 1 until the completion of blood sampling 3 hours after the administration.

The investigator or subinvestigator will instruct subjects to avoid intake of caffeine (including in drinks), alcoholic beverages, grapefruit (including in drinks), and St. John’s wort during the investigational product administration period. The investigators will instruct subjects to comply throughout the study with any dietary therapy prescribed before the study.

### 10.3.3 Contraception

The investigator or subinvestigator will instruct women of child-bearing potential to use an appropriate method of contraception from the time of consent until 12 weeks after the final administration of the investigational product, and instructed men who have reproductive capability to do so from the investigational product administration date until 12 weeks after the final administration of the investigational product. Appropriate methods of contraception are any two of the following methods: condom, oral contraceptives, intrauterine contraceptive device, pessary, spermicide, cervical cap, or contraceptive sponge. Abstinence was also permitted. The investigator or subinvestigator will provide a full explanation of the risks of pregnancy and appropriate methods of contraception to the subjects.

# 11 Report of Adverse Events

## 11.1 Adverse Events to be Reported

### 11.1.1 Definition of serious adverse events

See Section “[9.7.1.1 Definition of Serious Adverse Events.](#_9.7.1.1_Definition_of)”

### 11.1.2 Actions to take upon the occurrence of an adverse event and follow-up investigation

#### 11.1.2.1 Actions for subjects

The investigator or subinvestigator will take proper measures as necessary to ensure the safety of subjects such as the supply of appropriate therapy or discontinuation of a study when an adverse event occurs.

#### 11.1.2.2 Report to related parties

##### 11.1.2.2.1 In the case of serious adverse events

1) The investigator or subinvestigator will immediately (within 24 hours of knowing the occurrence of the concerned event) report the occurrence of a serious adverse event to the Emergency Safety Information Reception Center or the Sponsor via oral communication, telephone, e-mail, or facsimile irrespective of the causal relationship with the administration of the investigational product (Attachment 4 in the protocol [separate volume] “Report on serious adverse event (initial report)” or the form specified by the concerned investigative site). The investigator will submit a report on the serious adverse event (detailed report) to the Sponsor and director of the investigative site within 7 days of knowing about the occurrence of the concerned event. In doing so, a serious and unforeseeable adverse drug reaction will be specified to the director of the investigative site.

2) The investigator will supply additional information to the Sponsor, director of the investigative site, and IRB when requested.

3) The director of the investigative site will consult IRB concerning the appropriateness of continuing the clinical study at the concerned site.

| Emergency contact information  Emergency Safety Information Reception Center  Bell Medical Solutions, Inc.  Address: Tokyu Bldg. East No. 3, 2-16-8 Minami-Ikebukuro, Toshima-ku, Tokyo 171-0022  Phone: 0120-49-2934 Facsimile: 0120-49-2935  Office hours: 24 hours (throughout the year) |
| --- |

##### 11.1.2.2.2 Non-serious adverse event

Upon the occurrence of an adverse event that requires special attention, the investigator or subinvestigator will promptly report details and actions taken to the Sponsor. Details of other adverse events will be reported to the Sponsor as appropriate.

#### 11.1.2.3 Follow-up of adverse events

Adverse events will be followed up until recovery to the baseline state, or the investigator or subinvestigator judges that observation of the course is not necessary based on the symptoms, findings, laboratory data, etc. An adverse event related to “blood calcium decreased” will be followed up until the corrected serum Ca level becomes ≥8.4 mg/dL as a guide.

The information from the time of administration of the investigational product up to Day 29 will be inputted in the electronic case report form.

#### 11.1.2.4 Handling of pregnancy

Subjects should be instructed that the pregnancy of a female subject or a partner of a male subject should be promptly reported to the investigator or subinvestigator. The investigator or subinvestigator will take proper measures for ensuring safety such as discontinuation of the study as necessary, and follow up the event until observation of the course is judged unnecessary.

# 12 Discontinuation Criteria and Procedures

## 12.1 Discontinuation of a Relevant Subject

### 12.1.1 Discontinuation criteria

If any of the following criteria is met after enrollment, the study will be discontinued for the subject in question.

1) The subject is found to be ineligible because enrollment criteria are not met.

2) The subject asked to discontinue the study.

3) It is found not to be feasible to conduct necessary observations and tests because of the subject’s private reasons.

4) The investigator or subinvestigator determines that the subject should be discontinued from the study because of the occurrence of an adverse event.

5) Corrected serum Ca level decreased to ≤7.5 mg/dL.
If deemed necessary, the investigator or subinvestigator is to give appropriate treatment such as intravenous infusion of a Ca preparation as required in the event of discontinuation for this reason.

6) Other situations where the investigator or subinvestigator determines that the subject should be discontinued from the study.

### 12.1.2 Discontinuation Procedures

If a subject discontinues the study because of safety concerns such as adverse events, the investigator or subinvestigator will take appropriate action for the subject. In addition, the investigator or subinvestigator will confirm safety in each discontinued subject and conduct examinations promptly upon discontinuation.

Any subject who does not return to the investigative site after receiving study treatment will be followed up to the extent possible, in a way that protects the subject’s human rights, and the predetermined assessment will be made.

The investigator or subinvestigator identifies the date of discontinuation and the reason for the discontinuation and records the information in the electronic case report form.

## 12.2 Discontinuation or Suspension at the Investigative Site

When the clinical study at the investigative site is discontinued or suspended for reasons such as there is a suspicion of safety regarding the investigational product, the investigator will promptly report it to the director of the investigative site in writing, and provide a detailed written explanation concerning study discontinuation or suspension. The director of the investigative site will immediately report study discontinuation or suspension and the reason in writing to the Sponsor and IRB.

The clinical study at the concerned investigative site will be discontinued in the following cases, and appropriate measures will be taken as described above.

1) When protocol amendment is instructed by the director of the investigative site based on the opinions of the IRB consulted by the director of the investigative site, but the Sponsor does not approve it.

2) When the IRB that is consulted by the director of the investigative site judges that the study should not be continued, and the director of the investigative site instructs study discontinuation.

3) When a serious or continuous violation of the GCP, this protocol, or the study contract occurs at the investigative site.

## 12.3 Discontinuation or Suspension of the Entire Study

When discontinuing or suspending the entire study in the course of a clinical study, the Sponsor will promptly notify all directors of the investigative sites and regulatory authority together with the detailed reason. When the director of the investigative site receives such a notice, he/she will promptly inform the investigator and IRB in writing together with the reason. When the study is discontinued or suspended, the investigator will promptly inform the subjects, and take necessary action such as supplying appropriate medical care.

# 13 Endpoints

## 13.1 Efficacy

### 13.1.1 Primary endpoint

• Intact PTH level change rate at the end of investigational product administration

### 13.1.2 Secondary endpoints

• Number and percentage of subjects achieving a decrease in intact PTH level of ≥30% (rate of change ≤-30%) at the end of investigational product administration

• Number and percentage of subjects achieving an intact PTH level of ≤240 pg/mL at the end of investigational product administration.

• At the time of each test and at the end of administration of the investigational product, Intact PTH level, whole PTH level, corrected serum Ca level, ionized Ca level, serum P level, intact FGF23 level, and corrected serum Ca-P product

### 13.1.3 Exploratory endpoints

• Bone metabolic markers at the end of investigational product administration and at the end of follow-up

• Parathyroid gland (volume and blood flow) at the end of investigational product administration

## 13.2 Safety endpoints

• Adverse events

• Lowest corrected serum Ca level from the start of investigational product administration until the end of follow-up

• Laboratory values

• Vital signs

• 12-Lead electrocardiography

• Ophthalmologic examination

## 13.3 Pharmacokinetic endpoint

• Plasma KHK7580 level

# 14 Statistical Analysis

## 14.1 Statistical Methods

The major efficacy and safety endpoints and their analytical methods are as follows. As a general rule, categorical data are summarized using frequencies and percentages, and continuous data are summarized using descriptive statistics consisting of the number of subjects, mean, standard deviation, minimum, median, and maximum.

### 14.1.1 Efficacy analysis

#### 14.1.1.1 Primary endpoint

Regarding the rate of change in intact PTH level at the end of investigational product administration from pre-dose of investigational product on Day 1, the dose response profile will be investigated using seven contrast patterns for mean values in the KHK7580 0.5 mg group, 1 mg group, 2 mg group, and placebo group ([Table 14.1.1.1-1](#OLE_LINK1)). Resampling (permutation: number of extractions: 30,000, random seed: 7580005) will be used to adjust the multiplicity of the test. The significance level is a two-tailed p value of 5%. This is the principal analysis used for the study.

Table 14.1.1.1-1 Dose Response Profile (Contrast Pattern)

| Contrast pattern | Placebo group | KHK7580  0.5 mg group | KHK7580  1 mg group | KHK7580  2 mg group |
| --- | --- | --- | --- | --- |
| Monotonic decrease | 3 | 1 | -1 | -3 |
| Decrease from 1 mg | 3 | 3 | -1 | -5 |
| Decrease from 2 mg | 1 | 1 | 1 | -3 |
| Monotonic decrease (but 0.5 mg and 1 mg are roughly similar) | 1 | 0 | 0 | -1 |
| Monotonic decrease (but 1 mg and 2 mg are roughly similar) | 5 | 1 | -3 | -3 |
| Monotonic decrease (but 0.5 mg, 1 mg, and 2 mg are roughly similar) | 3 | -1 | -1 | -1 |
| Monotonic decrease (but placebo and 0.5 mg, and 1 mg and 2 mg are roughly similar) | 1 | 1 | -1 | -1 |

Regarding the rate of change in intact PTH level at the end of investigational product administration from pre-dose of investigational product on Day 1, the mean and 95% confidence interval for each group will be calculated by analysis of variance with treatment group as the factor. Descriptive statistics for each treatment group will be also shown. In addition, mean difference from the placebo group and 95% confidence interval will be calculated for the KHK7580 0.5 mg, 1 mg, and 2 mg groups and the KRN1493 25 mg group. Further, mean difference from the KRN1493 25 mg group and 95% confidence interval will be calculated for the KHK7580 0.5 mg, 1 mg, and 2 mg groups.

#### 14.1.1.2 Secondary Endpoints

The number and percentage of subjects who achieved the following and the exact 95% confidence interval for the percentage will be calculated for each treatment group.

• Achievement of a decrease in intact PTH level of ≥30% (rate of change from prior to investigational product administration on Day 1: ≤-30%) at the end of investigational product administration

• Achievement of an intact PTH level of ≤240 pg/mL at the end of investigational product administration

Descriptive statistics by timing of test and at the end of investigational product administration are shown for the following items by treatment group.

• Intact PTH level

• Whole PTH level

• Corrected serum Ca level

• Ionized Ca level

• Serum P level

• Intact FGF23 Level

• Corrected serum Ca-P product

### 14.1.2 Analysis of Safety

All adverse events that occurred after the start of investigational product administration will be examined, and incidence rates will be summarized by treatment group according to the adverse event, whether or not a causal relationship could be ruled out, and the incidence rate by details. Incidence rate summarization by details will be performed by MedDRA/J SOC and PT. Summarization by severity will be also performed in a similar fashion. The mean and 95% confidence interval for each treatment group will be calculated for the following items by analysis of variance with treatment group as the factor. Descriptive statistics for each treatment group will be also shown.

• Lowest corrected serum Ca level from the start of investigational product administration until the end of follow-up

Descriptive statistics for laboratory values and vital sign measurements will be calculated for each item and each treatment group, and line graphs will be prepared. For ophthalmologic examination, a shift table before and after administration will be prepared. Descriptive statistics for electrocardiographic measurements will be calculated for each item and each treatment group, and line graphs will be prepared. For QTcF and QTcB, the number and percentage of subjects falling under each of the following categories will be shown for each treatment group.

• Test value after the start of administration (msec): <450, ≥450 and <480, ≥480 and <500, ≥500

• Change after the start of administration* (msec): <30, ≥30 and <60, ≥60

* Change = Test value at each assessment point - baseline test value

## 14.2 Target number of subjects

150 subjects (30 subjects in each group) as subjects for investigational product administration

## 14.3 Significance Level Used

The significance level in the primary endpoint is a two-tailed p value of 5%. Also, if exploratory test methods are used, the guideline two-tailed p value is 5%.

## 14.4 Study Discontinuation Criteria

No statistically-based study discontinuation criteria are stipulated.

## 14.5 Handling of Missing, Unused, and Abnormal Data

Data for the analyses shown in Section [14.1](#_14.1_Statistical_Methods) will be handled as shown below. If data that are inconsistent with this are obtained, or handling needed to be reviewed for each subject because of an unexpected event at the start of the study initiation, a decision on handling will be taken before the database lock.

1) If any data are missing at the end of investigational product administration during summarization for the primary endpoint and secondary endpoints, data in the data obtained from after investigational product administration until the end of investigational product administration will be supplemented by Last Observation Carried Forward (LOCF) using data from the final time point as the investigational product administration end time.

2) Binary response variables such as the secondary endpoint “Achievement of an intact PTH level of ≤240 pg/mL at the end of investigational product administration” will be handled as non-achieving subjects if the investigational product administration end time is missing.

## 14.6 Development of Statistical Analysis Plan and Procedure for Reporting Deviations from the Original Analysis Plan

The details of the final statistical analysis plan, including the analyses described in Section [14.1](#_14.1_Statistical_Methods), will be finalized as the analysis plan prior to finalization of the data. Major changes in the statistical plan will be described in the clinical study report.

## 14.7 Selection of Subjects Included in Analysis Sets

The following populations will be defined as analysis sets. Decisions on whether to include individual subjects in analysis sets will be reached before the database lock.

### 14.7.1 Full analysis set (FAS)

The FAS is the set of enrolled subjects excluding those meeting any of the following criteria. The primary endpoint for efficacy will be established to confirm the stability of the main analysis.

• Subjects who have not been randomized

• Subjects who has never been treated with the investigational product

• Subjects whose intact PTH level prior to the start of investigational product administration is not available, or for whom no intact PTH level is available after the start of investigational product administration

### 14.7.2 Per protocol set (PPS)

The PPS is the set of subjects in the FAS excluding those meeting any of the following criteria, and is used for the principal analysis of efficacy.

• Subjects who do not meet selection criteria or meet exclusion criteria that may affect the efficacy evaluation.

• Subjects with a drug compliance of <70% from the start of investigational product administration until the end of administration
Drug compliance (%) = 100 x Number of tablets administered/Stipulated number of tablets (21 tablets)

• Subjects with major protocol violations that may affect the efficacy evaluation.

### 14.7.3 Safety analysis set

The safety analysis set is the set of enrolled subjects excluding subjects who meet the following criterion.

• Subjects who have never been treated with the investigational product

### 14.7.4 Pharmacokinetic analysis set

The “pharmacokinetic analysis set” is the set of enrolled subjects excluding subjects who meet any of the following criteria.

• Subjects who have never been treated with KHK7580 0.5 mg, 1 mg, 2 mg, or placebo.

• Subjects for whom no measurements of plasma KHK7580 level are available.

# 15 Ethics

## 15.1 IRB

### 15.1.1 Review of feasibility of study implementation

The protocol, contents of information for subjects/informed consent form, and feasibility of study implementation will be reviewed from the viewpoints of ethics, science, and medical validity, and approved by the institutional review boards (IRB) consulted by the directors of the investigative sites prior to implementation of this study.

### 15.1.2 Continued review

The investigator will submit a summary of an ongoing clinical study in writing at least once a year or upon request by the IRB to the director of the investigative site to receive continued review by the IRB.

## 15.2 Selection of a Prospective Subject and Assurance of Safety

When selecting a prospective subject, the investigator or subinvestigator will carefully review the eligibility of subjects from the viewpoint of protection of human rights such as possible disadvantages by not participating in a clinical study (socially vulnerable persons).

The investigator or subinvestigator will judge subject enrollment based on the inclusion criteria when enrolling a subject to avoid enrollment of a subject for whom safety cannot be ensured.

The investigator and subinvestigator will ensure there is a method of emergency contact with the subject to grasp the health of the subject at all times during the study period, and try to collect and disseminate safety information possibly related to the investigational product. In the case an adverse event occurs, the safety of the subject should be ensured by providing appropriate medical care to the subject, and discontinuing administration of the investigational product when necessary.

## 15.3 Protection of Personal Information and Privacy of Subjects

When creating an electronic case report form, the investigator or subinvestigator will use the subject identification code for identifying each subject to protect the personal information of subjects.

A person belonging to an organization involved in a clinical study of the Sponsor should not disclose secrets of a subject obtained in the course of duties without a justifiable reason. Also, genetic information should be more carefully and appropriately handled by double-anonymization in consideration of its nature because blood samples are stored for pharmacogenomic tests in this study.

## 15.4 Timing and Method of Obtaining Informed Consent

### 15.4.1 Information for subjects and informed consent

#### 15.4.1.1 Information for subjects and informed consent

The investigator or subinvestigator will give a satisfactory explanation on the contents of the clinical study to a possibly eligible subject of this study prior to participation in this study based on the separately specified information for Subjects/Informed Consent Form. The trial collaborator may provide a supplementary explanation on this study at that time. After providing ample time for the subject to think about participation in this study, the investigator or subinvestigator will obtain voluntary written informed consent (separately prepared informed consent form) for study participation from the subject by the time of screening.

The investigator or subinvestigator will also explain the storage and use of blood samples based on the Information for Subjects/Informed Consent Form for storage and use of blood samples for pharmacogenomic tests (“Storage and Use of Blood Samples for Pharmacogenomic Tests”) to a subject who consents to study participation. The investigator or subinvestigator will obtain voluntary written informed consent for storage and use of blood samples after providing ample time for the subject to think about it.

The investigator or subinvestigator who provided information and the subject will write their name/affix their seal or sign and respectively date in the Informed Consent Form. When a trial collaborator gives a supplementary explanation, the trial collaborator will also write their name/affix their seal or sign and date in the Informed Consent Form.

When a subject cannot sign due to dysgraphia, oral consent will be obtained from the subject, and written consent will be obtained from his/her proxy consenter. In such a case, the date of obtaining consent from the proxy consenter, relationship, and the reason for obtaining consent from the proxy consenter (dysgraphia), and the date of obtaining oral consent from the subject will be recorded in the Informed Consent Form.

### 15.4.2 Storage of informed consent form

The Informed Consent Form will be prepared in triplicate unless a specific arrangement has been made by the investigative site. The investigator or subinvestigator will hand the subject a subject’s copy of the Informed Consent Form and Information for Subjects. The investigator or subinvestigator will submit a copy of the Informed Consent Form for the investigative site to the department such as the clinical trial secretariat determined by the investigative site, and store the investigator’s copy of the Informed Consent Form together with medical records.

## 15.5 Information for Subjects/Informed Consent Form

The Information for Subjects/Informed Consent Form should include the following information.

1) That the clinical study is conducted for the purpose of research

2) Study objective

3) Name, title, and contact information of the investigator

4) Study method

5) Expected beneficial effects of investigational product on the subject’s mental and physical health (or the absence of expected benefits if no such benefits), and injuries or inconveniences to the subject

6) The alternative procedure(s) or course(s) of treatment

7) Expected duration of the subject’s participation in the study

8) That the subject may withdraw from the study at any time

9) That the subject may refuse or withdraw from the study without penalty or loss of benefits to which the subject is otherwise entitled

10) That the clinical research associate(s), the auditor(s), and the IRB of the investigative site, etc. will be granted direct access to the source documents, without violating the confidentiality of the subject

11) That the subject’s identity will remain confidential

12) The person(s) at the investigative site to contact in the event of study-related health injury

13) That in the event of study-related health injury, necessary treatment will be given to the subject

14) The compensation available to the subject in the event of study-related injury

15) The types of IRBs that will investigate and review the propriety of the study and others, issues to be investigated and reviewed at each IRB, and other matters related to the IRBs involved in the study

16) Other necessary matters related to the study

Neither of the following should be included in the Information for Subjects/Informed Consent Form:

1) Words and phrases that expressly or impliedly, make potential subjects waive their rights

2) Words and phrases that expressly or impliedly, discharge the Sponsor, investigative site, investigator, or subinvestigator from any liability that they may have, or reduce such liability.

The Information for Subjects/Informed Consent Form for “Storage and Use of Blood Samples for Pharmacogenomic Tests” must contain the following items.

1) The objectives of storage and use of blood samples

2) The relationship to the study

3) The target subjects

4) The fact that consent to the storage and use of blood samples is voluntary, and that refusal will have no effect on participation in the study

5) Timing and method of provision of blood samples

6) The start timing and method of the pharmacogenomic tests

7) The timing and method of storage and disposal of blood samples

8) Measures for the protection of personal information

9) The advantages, disadvantages and burdens associated with this consent

10) Withdrawal of consent

• The fact that consent can be withdrawn at any time

• The fact that samples will be disposed of promptly upon withdrawal of consent, but pharmacogenomic test data obtained up to the withdrawal of consent will not be disposed of

• The fact that withdrawal of consent will have no effect on participation in the study

11) The fact that source data including pharmacogenomic test results can be viewed by monitors, editors, institutional review boards, and the regulatory agency

12) The fact that test results will not be disclosed

13) Publication of test results

14) Rights arising from research and development

## 15.6 Supply of Information to Subjects, Revision of Information for Subjects/Informed Consent Form and Reacquisition of Consent

When new information that might affect the intention of a subject to continue to participate in the study (such as information on variations of the subject’s laboratory data) is obtained, the investigator or subinvestigator will immediately explain it to a subject participating in the study, confirm his/her intention to continue to participate in the study, and record the explanation given, date of explanation, and name of the person who confirmed the subject’s intention, and the intention in the medical record.

When revision of the Information for Subjects/Informed Consent Form is judged necessary concerning the explanation, the investigator will immediately revise the Information for Subjects/Informed Consent Form, and obtain the approval of the IRB. Then, the investigator or subinvestigator will newly explain to the subject using the revised Information for Subjects/Informed Consent Form, and obtain written consent again from the subject to continue to participate in the study according to the same procedures as those for the initial acquisition of informed consent.

# 16 Monetary Payment and Compensation for Health Injury and Liability

## 16.1 Study-related expenses

The sponsor will pay expenses for all tests and imaging diagnosis, drugs and injections having the planned indication or similar indication of the concerned investigational product to be administered during the period of investigational product administration based on the Special or Specified Medical Care Coverage for the clinical study.

## 16.2 Expenses for reducing burden of subjects associated with study participation

The sponsor will pay expenses for reducing burden of a subject associated with study participation based on the rules of the investigative site to a subject via the investigative site.

## 16.3 Compensation for health injury and liability

1) In the case any study-related health injury occurs to a subject, the investigator and subinvestigator will take necessary measures such as treatment, and the Sponsor will pay the amount of self-pay of the subject for medical expenses required for treatment after excluding health insurance benefits for the treatment upon request by the subject.

2) In the case any study-related health injury occurs to a subject and a dispute arises or might possibly arise with a subject later on, the investigative site will immediately report it to the Sponsor, and the investigative site and the Sponsor will cooperate for its settlement.

3) In the case any study-related health injury occurs to a subject and liability arises later on, the Sponsor will pay the compensation paid by the investigative site and the expenses required for settlement except for the case where the investigative site is responsible.

4) In the case any study-related health injury occurs to a subject and liability arises later on, the Sponsor will pay liability.

5) The liability in the preceding paragraph will be conducted according to the Relief System for Sufferers from Adverse Drug Reactions.

# 17 Compliance with the Protocol and Deviation/Modification and Revision

## 17.1 Compliance with the Protocol

The investigator and subinvestigator will comply with the protocol that was agreed on between the investigator and the Sponsor, and approved in writing by the IRB consulted by the director of the concerned investigative site.

## 17.2 Protocol Deviation or Modification

The investigator or subinvestigator should not make a protocol deviation or modification without prior written agreement between the investigator and the Sponsor, and written approval of IRB based on prior review. In the case any protocol deviation or modification is made, the investigator or subinvestigator will record all deviations irrespective of reasons.

The investigator or subinvestigator may make a protocol deviation or modification without prior written agreement with the Sponsor, and the prior approval of IRB in medically necessary case for avoiding emergent risk to a subject. In such a case, the investigator will prepare a document for recording the details of deviation or modification and reasons, and immediately submit it to the Sponsor and director of the investigative site. The investigator will also submit the document to IRB via the director of the investigative site to obtain approval, and the written approval of the director of the investigative site and the agreement of the Sponsor.

When revision of the protocol is judged appropriate based on the contents and reasons for deviation or modification, the investigator will submit a draft as soon as possible to the Sponsor and director of the investigative site and to IRB via the director of the investigative site to obtain approval, and obtain the written approval of the director of the investigative site and the agreement of the Sponsor.

## 17.3 Protocol Amendments

If the Sponsor has amended the protocol, the Sponsor will fully inform the investigator using the amended version of the protocol or a written description of the amendment to obtain their agreement. The investigators will conduct the study according to the amended protocol after receiving the written approval of the IRB based on its prior review. However, this does not apply to amendments that involved only administrative issues (e.g., changes in affiliation, job title, address, or telephone number).

# 18 Precautions for Creation of Electronic Case Report Form

## 18.1 Creation of Electronic Case Report Form and Reporting

This study will use the electronic data collection (EDC) system for collection of data. The EDC system has the functions for the investigative site such as inputting data in an electronic case report form, inspecting inputted data, replying to an inquiry from the Sponsor on the inputted data, and creating electronic signatures. Inputted data are encrypted and transmitted to the EDC server via the Internet. This EDC system was confirmed in advance by the Sponsor to comply with the requirements of “The Ministerial Ordinance for Good Clinical Practice (Ordinance No. 28 of the Ministry of Health and Welfare dated March 27, 1997),” the ordinance for partial revision, and “Use of Electronic Records and Electronic Signatures in Submission for Approvals, Licenses of Medical Products (MHLW/PFSB Notification No. 0401022 dated April 1, 2005).”

The investigator will prepare an electronic case report form for each subject, check the accuracy and integrity of all the data, and create an electronic signature on the EDC system. When the electronic case report form was created by the subinvestigator or trial collaborator, the investigator will check the contents to confirm that there is no problem prior to creating an electronic signature. The electronic case report form will be created according to the “Guide for changing or correcting electronic case report forms” provided by the Sponsor.

The subject data stored on the EDC server will be handled as the original electronic case report form in this study. However, when the electronic case report form is transferred from the EDC server to a medium that cannot be overwritten (DVD, etc.), the electronic case report form in the medium will be handled as the original.

The sponsor will provide the electronic case report form and a copy of the history of changes or corrections to the investigative site.

## 18.2 Change or Correction of Electronic Case Report Form

The investigator, subinvestigator, or trial collaborator will comply with the “Guide for changing or correcting electronic case report forms” provided by the Sponsor when making a change or correction to the data inputted in the electronic case report form. The history of a change or correction to the electronic case report form will be automatically created by the EDC system.

## 18.3 Items Inputted in the Electronic Case Report Form That May be Handled as the Source Material (source data)

The following items may be directly inputted in the electronic case report form, and the contents may be handled as the source material (source data). When a comparable description has been recorded in the medical chart, the contents will be handled as the source material (source data).

1) Adverse event name, severity, seriousness, action, date of outcome, outcome, causal relationship with the investigational product and comments

2) Reason for using a concomitant drug, comments on treatment incompliance

3) Date of discontinuation, reason for discontinuation and comments

4) Special items other than the above, and the comments of the investigator or subinvestigator

# 19 Direct access to source materials

The investigator, subinvestigator and the investigative site that conducted this study are required to make available all the study-related records such as the source materials for direct access at the time of monitoring and audit by the Sponsor, and inspection by the IRB and regulatory authority.

# 20 Quality Control and Quality Assurance of Clinical Study

The Sponsor will check through monitoring and audit to determine whether the study is implemented and data are created/recorded/reported in compliance with the protocol and the Ordinance of Ministry of Health and Welfare No. 28 dated March 27, 1997 “The Ministerial Ordinance on Good Clinical Practice for Drugs” (GCP Ordinance).

Data will be controlled and assured in compliance with the standard operating procedures (SOP) for study implementation and audit procedures created by the Sponsor.

The Monitor will check the implementation of the study in compliance with the GCP Ordinance, this protocol and SOP for management of the investigational product, and consistency between the data inputted in the electronic case report form and source materials. Details of specific procedures will be explained in the separately prepared monitoring plan or alternative procedures.

# 21 Study Period

From July 2014 to June 2015

# 22 Study Completion

The investigator will report the study completion and summary of study results to the director of the investigative site after completion of the protocol-specified administration and observation in all subjects at the relevant investigative site.

The director of the investigative site will promptly inform study completion to IRB and the Sponsor in writing, and report the summary of study results based on the report submitted by the investigator.

# 23 Storage of Records

## 23.1 Storage at IRB

**1) Materials to be stored**

The founder of IRB will store the SOP, roster of IRB members (including the qualifications of each member), a list of occupations (job names) and affiliation of the members, submitted documents, minutes of meetings and summary, and records such as letters.

**2) Storage period**

The founder of the IRB will store the materials up to either of the following dates that occurs later. However, when the Sponsor requires a longer storage period, the duration and method of storage will be discussed with the Sponsor. When storage becomes no longer necessary, the Sponsor will inform it to the founder of IRB via the director of the investigative site.

• The date of obtaining marketing approval for the concerned investigational product (when development is discontinued, 3 years from the date of decision of development discontinuation)

• Three years from the date of study discontinuation or completion

## 23.2 Storage at the investigative site

**1) Materials to be stored**

The director of the investigative site will appoint storage managers for each record, and store the documents or records related to the study to be archived at the investigative site.

**2) Storage period**

The director of the investigative sites stores materials up to either of the following dates that occurs later. However, when the Sponsor requires a longer storage period, the duration and method of storage will be discussed with the Sponsor. When storage becomes no longer necessary, the Sponsor will inform it to the director of the investigative site.

• The date of obtaining marketing approval for the concerned investigational product (when development is discontinued, 3 years from the date of decision of development discontinuation)

• Three years from the date of study discontinuation or completion

## 23.3 Storage by the Investigator

The investigator will store study-related documents or records according to the instructions of the director of the investigative site.

## 23.4 Storage by the Sponsor

**1) Materials to be stored**

The Sponsor will store the study-related documents or records to be archived.

**2) Storage period**

The Sponsor will store the study-related documents or records up to any of the following dates that occurs the latest.

• Five years from the date of obtaining marketing approval for the concerned investigational product (when development is discontinued, 3 years from the date of decision of development discontinuation), or the date of completion of reexamination for a drug that is subjected to reexamination after approval according to the stipulations of the Pharmaceutical Affairs Law and the period up to completion of the reexamination exceeds 5 years.

• Three years from the date of study discontinuation or completion

## 23.5 Storage of Source Materials Related to Measurement of Plasma KHK7580 Level

The source materials related to the measurement of the plasma KHK7580 level will be stored up to any of the following dates that occurs later.

• Five years from the date of obtaining the marketing approval of the concerned investigational product (3 years from the date of receiving a notification of development discontinuation in case of development discontinuation), or the date of completion of reexamination for a drug that is subjected to reexamination after approval according to the stipulations of the Pharmaceutical Affairs Law and the period up to completion of the reexamination exceeds 5 years.

• Three years from the date of study discontinuation or completion

Storage site: Archives, Sumika Chemical Analysis Service, Ltd.
3-1-135 Kasuga Denaka, Konohana-ku, Osaka-shi, Osaka, Japan

## 23.6 Storage of Source Materials Related to Laboratory Test, etc.

The source materials related to laboratory tests should be stored until either of the following dates that occurs later.

• Five years from the date of obtaining marketing approval of the concerned investigational product (3 years from the date of receiving notification of the development of discontinuation in the case of the development discontinuation), or the date of completion of reexamination for a drug that is subjected to reexamination after approval according to the stipulations of the Pharmaceutical Affairs Law and the period up to completion of reexamination exceeds 5 years.

• Three years from the date of study discontinuation or completion

## 23.7 Storage of Biological Samples

1) The samples collected for hematology tests will be stored and disposed of after measurement according to the procedures of LSI Medience Corporation. The samples collected for blood biochemical test-1 will be stored and disposed of according to the procedures of LSI Medience Corporation.

2) The plasma samples for pharmacokinetic analysis will be stored at Sumika Chemical Analysis Service, Ltd. until completion of the clinical study report, and subsequent handling will be separately discussed.

3) The samples collected for blood biochemical test-2 and bone metabolism markers will be stored at LSI Medience Corporation until completion of the test result report, and subsequent handling will be separately discussed.

## 23.8 Storage of Blood Samples for Use in Pharmacogenomic Tests

The samples obtained from a subject who consented to storage and use of blood samples for pharmacogenomic tests will be stored until either of 15 years after the date of completion of the specified tests for the last subject, the time when pharmacogenomic tests are judged unnecessary, or the time of withdrawal of consent on storage and use of blood samples by a subject, whichever comes the earliest, and will be disposed of after taking action for preventing the use by others for other research, etc.

# 24 Publication of Study Results

When publishing the results obtained in this study at academic society meetings or medical journals, etc., the investigator and subinvestigator will obtain the prior approval of the Sponsor.

# 25 Rationale

## 25.1 Rationale for Establishing Study Design

This study is designed as a placebo-controlled, parallel-group, dose response study to investigate the efficacy and safety of 3 doses of KHK7580 (0.5 mg, 1 mg, and 2 mg) in SHPT patients receiving hemodialysis. It is also designed as a double-blind study to restrict the impact of the information of the allocated investigational product on efficacy endpoints. The primary endpoint of this study is investigation of the dose response profile of KHK7580 group, but KRN1493 25 mg group is determined as an open-label group for the purpose of confirming study sensitivity and study conduct within an appropriate dose range, to compare efficacy and safety between placebo group and KHK7580 group.

## 25.2 Rationale for Establishing Inclusion Criteria

1) This criterion is necessary for the study to comply with the GCP Ordinance.

2) The legally established age of individual consent for the study is 20 years, and subjects are to be under 75 years from feasibility point of view.

3) This criterion is established because patient symptoms are unstable immediately after dialysis initiation and could potentially affect the evaluation of the safety of KHK7580.

4) The criterion of an intact PTH level ≥240 pg/mL is established because the "Clinical Practice Guideline for CKD-MBD^3)”^ recommends that intact PTH level in SHPT patients be managed within the range of 60 pg/mL to 240 pg/mL.

5) The results of clinical studies of KHK7580 in healthy adults and SHPT patients receiving hemodialysis and a pharmacology study in partially nephrectomized rats suggest that serum Ca levels decrease in association with administration of KHK7580. The criterion of a corrected serum Ca level ≥ 8.4 mg/dL, which is the lower limit of the target range, is established in consideration of the safety of subjects and because the "Clinical Practice Guideline for CKD-MBD^3)^" recommends Ca level in SHPT patients be managed within the range of 8.4 to 10.0 mg/dL.

## 25.3 Rationale for Establishing Exclusion Criteria

1 to 5) These criteria are established to properly evaluate the efficacy, safety, and pharmacokinetics of KHK7580.

6 to 8, and 10 to 12)
 These criteria are established to ensure subject safety and to properly evaluate the safety of KHK7580.

9) This criterion is established in consideration of the safety of subjects and their offspring because the reproductive safety of KHK7580 has not been established.

13-14) These criteria are established to ensure subject safety and to properly evaluate the efficacy, safety, and pharmacokinetics of KHK7580.

15) This criterion is established because hyperparathyroidism primary is not a target disease.

16) This criterion is established to exclude subjects who are determined by the investigator or subinvestigator to be ineligible for participation in the study for a reason other than those above.

## 25.4 Rationale for Establishing Dose, Method and Duration of Administration

A comparison was made of outcomes in a Phase 1/2 clinical study of KHK7580 performed in Japan (7580-003 Study) and a Phase 2 clinical study of KRN1493 performed in Japan (KRN1493/03-A06 Study)^6)^. The results suggested that 1 mg of KHK7580 is equivalent to a clinical dose (25 mg) of KRN1493 in terms of the rate of change in intact PTH level. As discussed in the ICH E4 Guideline, evaluation of dose response is an indispensable part of drug development, and a KHK7580 doses of 0.5 mg and 2 mg are therefore established at a factor of 2 around the 1 mg dose expected to be equivalent to the 25 mg starting dose of KRN1493 to obtain information on the starting dose for KHK7580. Regarding the method and duration of administration, the KRN1493/03-A06 study found that oral administration once a day for 3 weeks produced a significant rate of change in intact PTH level in the KRN1493 25 mg group.

## 25.5 Rationale for Establishing Prohibited Concomitant Medications and Therapies

Prohibited concomitant medications and therapies are established in order to properly evaluate the efficacy and safety of KHK7580.

## 25.6 Rationale for Establishing Restricted Concomitant Medications and Therapies

1) These criteria are established in order to properly evaluate the efficacy and safety of KHK7580. Because of the similarity of indications, changes in dosage and administration are restricted throughout the study.

2) These criteria are established in order to properly evaluate the efficacy and safety of KHK7580.

## 25.7 Rationale for Establishing Measurement of Plasma KHK7580 Level

Two time points of 1 and 3 hours after investigational product administration on Day 1 are established, since these times are deemed to enable broad fluctuations in plasma KHK7580 level to be understood in view of the fluctuations in plasma KHK7580 level in healthy adults and SHPT patients receiving hemodialysis obtained from Phase 1 clinical studies of KHK7580 (7580-001 Study, 7580-002 Study) and a Phase 1/2 clinical study (7580-003 Study) performed in Japan. In addition, measurements are to be performed on the dialysis date from Day 1 until Day 8, and on the dialysis date after the longest interval between dialysis sessions from Day 8 until the stipulated final assessments to enable fluctuations in plasma drug level after repeated administration to be understood.

## 25.8 Rationale for Establishing Instructions to Subjects

Subjects are instructed to avoid the intake of caffeine and alcohol during treatment with the investigational product because of their possible effect on drug metabolism in the liver and on laboratory data such as transaminase level. Also, the intake of grapefruit (including drinks containing grapefruit) or drinks containing St. John’s wart is to be avoided during treatment with the investigational product because the contained ingredient inhibits or induces CYP3A4/5 activity and thereby possibly affects the pharmacokinetics of KHK7580.

## 25.9 Rationale for Establishing Test Items

The intact PTH level, whole PTH level, corrected serum Ca level, Ca level, Alb level, ionized Ca level, P, intact FGF23 level and the product of corrected serum Ca and P level are established to investigate the efficacy of KHK7580. The lowest corrected serum Ca level is established to investigate the safety of KHK7580.

Bone metabolism markers are established as the exploratory endpoints to investigate the impact on the bone, and carotid ultrasonography to investigate the effect on the parathyroid volume and blood flow.

## 25.10 Rationale for Establishing Discontinuation Criteria

1) This criterion is established to ensure that subjects who should not have been included in the study would discontinue the study as soon as possible.

2) This criterion is established to protect human rights according to the Declaration of Helsinki.

3) This criterion is established to ensure that subjects who are unable to undergo required observations would discontinue the study as soon as possible.

4) This criterion is established in consideration of subject safety.

5) This criterion is established to ensure subject safety.

6) This is established to ensure that subjects who, in the opinion of the investigator or subinvestigator, should not continue to participate in the study for of reason other than criteria mentioned above would discontinue the study as soon as possible.

## 25.11 Rationale for Establishing Efficacy Endpoints

For treatment of SHPT, the dose of drugs such as cinacalcet hydrochloride is generally adjusted in consideration of reduction of intact PTH level as well as corrected serum Ca and serum P levels. The percentage change in intact PTH level is established as the primary efficacy endpoint in this study to investigate the dose response profile of KHK7580.

The number and percentage of subjects who achieved ≥30% reduction of intact PTH level (percentage of change ≤−30%) are established as the secondary efficacy endpoint in reference to the phase II clinical study (KRN1493/03-A06 Study)^6)^ and the phase III clinical study (KRN1493/04-A10 Study)^7)^ of KRN1493, an analog, and in consideration of changes in intact PTH level. It is recommended in the “Clinical Practice Guideline for CKD-MBD”^3)^ to control the intact PTH level in SHPT patients between ≥60 pg/mL and ≤240 pg/mL, the number and percentage of subjects who achieved the intact PTH level of ≤240 pg/mL are established. Also, the serum P level and the product of corrected serum Ca and P levels are added to the secondary efficacy endpoints because an increase in the product of corrected serum Ca and P levels is considered a risk factor of ectopic calcification in patients receiving hemodialysis, and appropriate control of serum P and Ca levels is recommended in the “Clinical Practice Guideline for CKD-MBD”^3)^. Further, intact FGF23 level is added to the secondary efficacy endpoints because of its relationship with intact PTH and serum P levels. A bone metabolism marker is also established as an exploratory endpoint to investigate the impact on bones.

## 25.12 Rationale for Establishing Target Number of Subjects

The following rates of change in intact PTH level are estimated at the end of administration in the placebo group and KHK7580 0.5 mg, 1 mg, and 2 mg groups with reference to outcomes in a Phase 1/2 clinical study of KHK7580 performed in Japan (7580-003 Study) and a Phase 2 clinical study of KRN1493 performed in Japan (KRN1493/03-A06 Study).

The results of the 7580-003 Study revealed that mean rate of change in intact PTH level at the end of repeated administration of 1 mg and 4 mg once a day for 14 days were −13.42% and −32.57%, respectively, and the standard deviations were 20.45% and 28.76%. In addition, the results from the KRN1493/03-A06 PPS revealed that mean rate of change in intact PTH level at the end of administration in the placebo group was 0.87% and the standard deviation was 22.47%.

On the basis of the above results, rate of change in intact PTH level at the end of administration in the present study is estimated as shown in [Table 25.12-1](#OLE_LINK2). The respective rates of change of −6.71% and −19.80% in the KHK7580 0.5 mg group and 2 mg group are linearly interpolated from the rate of change of 0% in the placebo group, the results at the end of repeated administration of 1 mg in the 7580-003 Study, and the results at the end of repeated administration of 1 mg and 4 mg in the 7580-003 Study. The standard deviation of 22.47% uses the standard deviation in the placebo group in the KRN1493/03-A06 Study.

Table 25.12-1 Estimated Rate of Change in Intact PTH Level at the End of Administration

| Group | Rate of change (%) | Standard deviation (%) |
| --- | --- | --- |
| Placebo group | 0 | 22.47 |
| KHK7580 0.5 mg group | −6.71 | 22.47 |
| KHK7580 1 mg group | −13.42 | 22.47 |
| KHK7580 2 mg group | −19.80 | 22.47 |

On the basis of the above, a significance level with a two-tailed p value of 5% is used, multiplicity is taken into consideration by resampling (permutation: number of extractions: 30,000, random seed: 7580005, number of simulations: 10,000), and the probability of any of the contrast patterns set being significant is about 90% in a group of 28 subjects. In view of incidences of subjects excluded from the PPS, target subject numbers are set at five groups with 30 subjects in each and 150 subjects in total who are treated with the investigational product. In view of the number of subjects in the KRN1493 25 mg group (28 subjects) who exhibited a significant rate of change in intact PTH level in the KRN1493/03-A06 Study, target subject numbers in the KRN1493 25 mg group are set at 30 subjects in the same way as in other groups for the purpose of comparison and evaluation of efficacy and safety with the KHK7580 groups.

# 26 Study Implementation System

See Attachment 1 in the protocol (separate volume).

# 27 Major Responsibilities of the Investigator

See Attachment 2 in the protocol (separate volume).

# 28 References

1. Tsubakihara Y. Pictorial Guide: An Overview of Regular Dialysis Treatment in Japan (As of December 31, 2012). Tokyo: Committee of Renal Data Registry, the Japanese Society for Dialysis Therapy; 2013. 65p.
2. National Kidney Foundation. K/DOQI clinical practice guidelines for bone metabolism and disease in chronic kidney disease. Am J Kidney Dis. 2003;42(4 Suppl 3): S1-201.
3. The Japanese Society for Dialysis Therapy. Clinical Practice Guideline for CKD-MBD [in Japanese]. Journal of Japanese Society for Dialysis Therapy. 2012;45(4) :301-56.
4. 2012 DOPPS Annual Report [homepage on the Internet]. [cited 2014 Apr 1]. Available from: http://www.dopps.org/annualreport/html/vitdgroup_c_Japan2011.htm
5. Package insert of Regpara^®^ Tablets 25mg/Regpara^®^ Tablets 75mg. Version 6: Kyowa Hakko Kirin Co., Ltd.; 2014.
6. Ishii Y. A randomized, double-blind, group-comparison, dose response study of KRN1493 in patients with secondary hyperparathyroidism receiving hemodialysis (phase II). Clinical Study Report. Kirin Brewery Company, Limited; 2006. Study No.: KRN1493/03-A06.
7. Kanno Y. A placebo-controlled, randomized, double-blind, comparative study of KRN1493 in patients with secondary hyperparathyroidism receiving hemodialysis (phase III). Clinical Study Report. Kirin Brewery Company, Limited; 2006. Study No.: KRN1493/03-A10.
